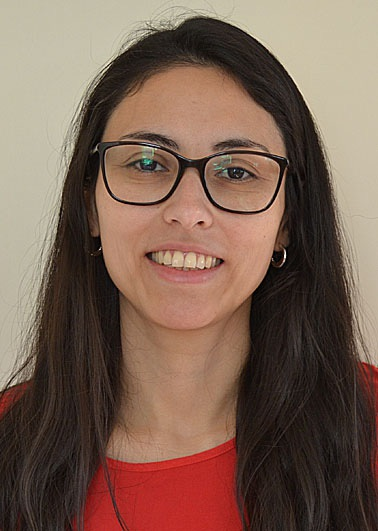# Isoindolinone Synthesis via One‐Pot Type Transition Metal Catalyzed C−C Bond Forming Reactions

**DOI:** 10.1002/chem.202004375

**Published:** 2021-02-02

**Authors:** Risto Savela, Carolina Méndez‐Gálvez

**Affiliations:** ^1^ Johan Gadolin Process Chemistry Centre Laboratory of Molecular Science and Technology Åbo Akademi University Biskopsgatan 8 20500 Turku Finland

**Keywords:** catalysis, heterocycles, isoindolinone, synthesis, transition metals

## Abstract

Isoindolinone structure is an important privileged scaffold found in a large variety of naturally occurring as well as synthetic, biologically and pharmaceutically active compounds. Owing to its crucial role in a number of applications, the synthetic methodologies for accessing this heterocyclic skeleton have received significant attention during the past decade. In general, the synthetic strategies can be divided into two categories: First, direct utilization of phthalimides or phthalimidines as starting materials for the synthesis of isoindolinones; and second, construction of the lactam and/or aromatic rings by different catalytic methods, including C−H activation, cross‐coupling, carbonylation, condensation, addition and formal cycloaddition reactions. Especially in the last mentioned, utilization of transition metal catalysts provides access to a broad range of substituted isoindolinones. Herein, the recent advances (2010–2020) in transition metal catalyzed synthetic methodologies via formation of new C−C bonds for isoindolinones are reviewed.

## Graphical abstract

**Isoindolinone motif can found** in a large variety biologically and pharmaceutically active compounds. Due to the central role in a number of applications, the synthetic methodologies for accessing this heterocyclic skeleton have received significant attention during the past decade. Herein, the recent advances (2010–2020) in transition metal catalyzed synthetic methodologies via formation of new C−C bonds for isoindolinones are reviewed.

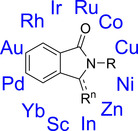

## Introduction

**Figure 1:**
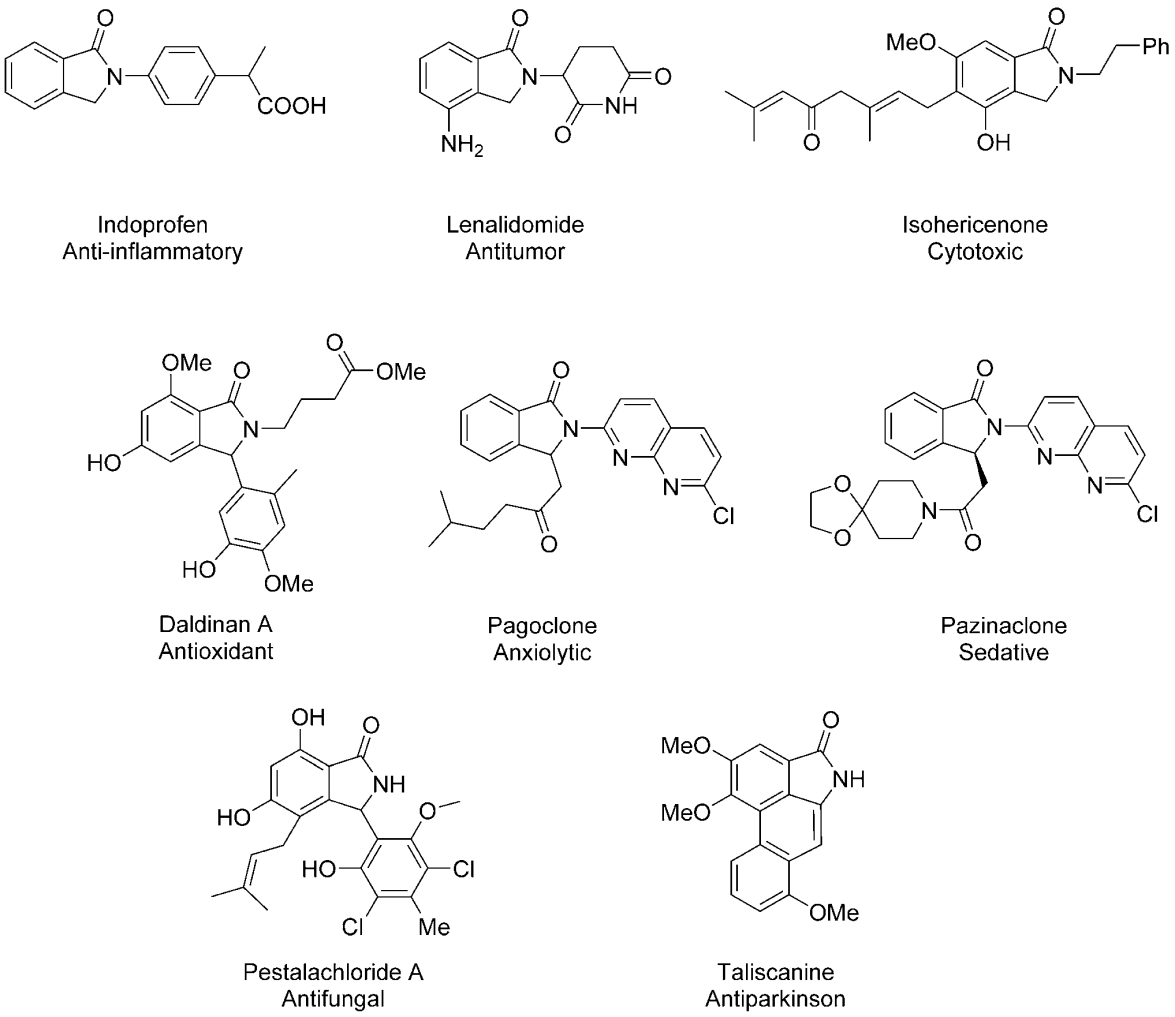
Examples of biologically active isoindolinone derivatives.

Isoindolinone scaffold is a structural motif found in a large variety of naturally occurring, as well as synthetic, biologically and pharmaceutically active compounds, as illustrated in Figure 1. Examples of the biological activities possessed by such compounds include antimicrobial,^[1]^ antioxidant,^[2]^ antifungal,^[3]^ antiparkinsons,^[4]^ anti‐inflammatory,^[5]^ antipsychotic,^[6]^ antihypertensive,^[7]^ anesthetic,^[8]^ vasodilatory,^[9]^ anxiolytic,^[10]^ antiviral,^[11]^ anticancer,^[12]^ selective Na_V_1.7 blocking^[13]^ and survival motor neuron protein production regulating^[14]^ activities. For example, lenalidomide, pagoclone and pazinaclone are clinically utilized pharmaceuticals. Isoindolinone derivatives also serve as important key intermediates for a number of highly useful organic compounds and natural products, but to obtain the prerequisite isoindolinone derivative have often required multistep synthesis strategies.[^1^, ^4^, ^5^, ^6^, ^7^, ^8^, ^9^, ^10^, ^11^, ^12^, ^13^, ^14^, ^15^]

A common strategy has been, and still is, based on functionalization of phthalimides or phthalimidines to synthesize the desired isoindolinone derivative.^[16]^ While this strategy does facilitate the access to diversely decorated isoindolinones, it can require multistep methodologies to achieve the target compound. During the past decade, a variety of catalytic methodologies have been investigated in order to minimize the number of reaction steps and to maximize the direct utilization of commercially available compounds for the synthesis of isoindolinone based structures.^[17]^ Especially transition‐metal catalyzed C−C bond formation with subsequent lactamization have provided simplified access to a large variety of isoindolinone derivatives. These reactions involve the use of C−H activation, carbonylation, cross‐coupling, addition, condensation, formal cycloaddition and annulation based strategies. In this minireview, we highlight the advances made during the past ten years in transition‐metal catalyzed C−C bond forming reactions applying one‐pot type strategies for isoindolinone synthesis.

## C−H Functionalization: Annulation/Cyclization

In general, the covalent bond between carbon and hydrogen (C−H) is considered fairly inert and the activation of this bond for modification has remained as a significant challenge to synthetic chemists. Thus, the C−H bond catalytic activation can be considered as one of the most important areas of modern synthetic chemistry due to its wide potential in various applications in both industrial and academic settings. In recent years, significant improvements in the synthesis of N‐heterocyclic compounds by C−H bond functionalization/activation have been made.^[18]^ A common feature in most of the methods utilizing C−H functionalization strategies for isoindolinone synthesis is the use of N‐substituted benzamides as reactants bearing either activating or directing groups. Most commonly featured groups consist from: *p*‐toluene sulfonate (Ts), methyl sulfonate (Ms), different alkoxides, 8‐quinoline (Q), 2‐pyridine or 2‐pyridine oxide and acetate (OAc) or pivalate (OPiv) moieties. In the following discussion, the different reactions forming isoindolinones via C−H functionalization are classified according to the transition metal used as the catalyst.

### Palladium catalyzed C−H functionalization

#### Pd‐catalyzed olefination/alkylation‐cyclization

**Scheme 1:**
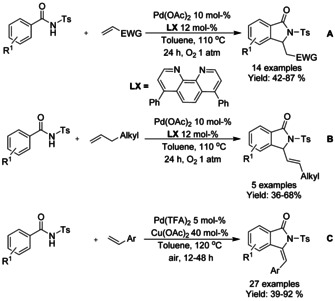
Palladium catalyzed C−H functionalization of tosylated benzamides.

Both Zhu^[19]^ and Youn^[20]^ have described the oxidative synthesis of isoindolinones using tosylated benzamides with a variety on alkene reactants and either palladium(II) acetate and palladium(II) trifluoracetate as catalysts (Scheme 1; Zhu: **A** and **B**; Youn: **C**). These methods utilize pure oxygen atmosphere or air as the oxidant but the method presented by Youn requires copper(II) acetate to oxidize the palladium catalyst as a part of the catalytic cycle. In general, these methodologies provide moderate to high yields for most reactant combinations but significant steric hindrance close to the reaction site seems to lower the yield of the reaction.

**Scheme 2:**
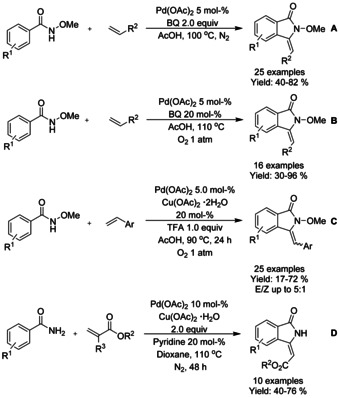
Oxidative C−H functionalization of benzamides with various alkenes.

A number of isoindolinone derivatives were prepared from *N*‐methoxy derivatized benzamides with alkenes by Li,^[21]^ Wrigglesworth,^[22]^ and Hii.^[23]^ (Scheme 2 A, B and C, respectively). Li and co‐workers utilized *p*‐benzoquinone (BQ) as the oxidant and Wrigglesworth and co‐workers a combination of BQ and oxygen. The *ortho*‐substituted benzamides and electron withdrawing substituents in general resulted in significant decrease in the isolated yields. Due to the genotoxic nature of *p*‐benzoquinone, the group of Hii applied a combination of copper(II) acetate/O_2_ as a suitable alternative, yielding 3‐alkylidene isoindolinones in good yields. Here, only the use of trifluoromethyl substituents was reported to drastically decrease the yield. Later, Laha^[24]^ described a similar method using underivatized benzamides with stoichiometric amount of copper(II) acetate as the oxidant and dioxane as solvent instead of acetic acid (Scheme 2 D).

**Scheme 3:**
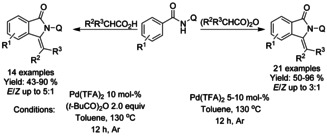
Decarboxylative strategies for synthesis of isoindolinones via palladium catalyzed C−H functionalization.

Carboxylic acid derivatives have also found utility as reactants in palladium catalyzed C−H functionalization of derivatized benzamides. Wie^[25]^ described a redox‐neutral C−H functionalization strategy between 8‐quinoline derivatized benzamides and carboxylic acids or carboxylic acid anhydrides (Scheme 3). In this manner, a broad range of 3‐alkylidene isoindolinones were synthesized in moderate to good yields.

#### Pd‐catalyzed isocyanide insertion‐cyclization

**Scheme 4:**
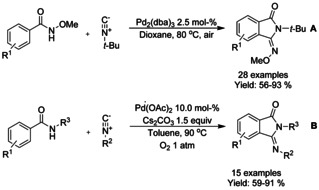
Use of isocyanides in palladium catalyzed C−H functionalization.

Dai and Yu^[26]^ utilized Pd_2_(dba)_3_ as a catalyst to synthesize 3‐imino‐isoindolinones in good yields from *tert*‐butylisocyanides and *N*‐methoxy benzamides using air as the oxidant. The methodology was able to overcome catalyst deactivation commonly associated with heterocycles in C−H activation and produce 3‐imino‐isoindolinones with heterocyclic substituents (Scheme 4 A). Interestingly, an unexpected reversal of the positions of the methoxy and *tert*‐butyl groups takes place with this catalytic system. Also, Wei and Qian^[27]^ utilized isocyanide derivatives in a similar manner but under oxygen atmosphere. Here, both methoxy and pentafluorobenzene based directing/activating groups (R^3^ in Scheme 4 B) could be utilized with either *tert*‐butyl‐ or isopropylisocyanides.

#### Pd‐catalyzed cyclization using acyl radicals

**Scheme 5:**
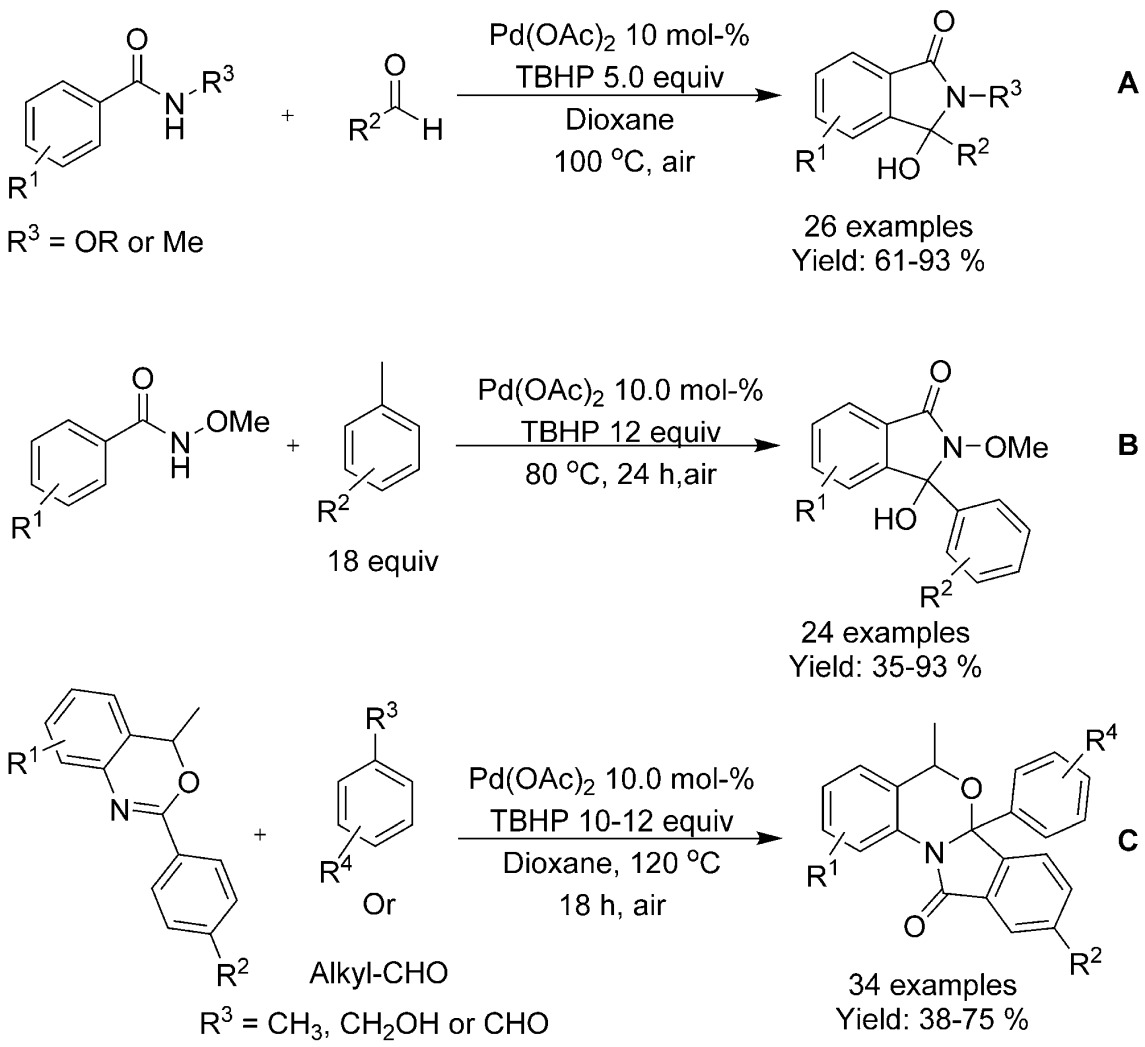
Aldehydes as substrates for the synthesis of isoindolinones via palladium catalyzed C−H activation.

Similarly to alkenes, aldehydes have also been found as useful substrates for isoindolinone synthesis. Zhao and Huang^[28]^ successfully utilized a variety of aldehydes together with N‐derivatized benzamides for synthesis of 3‐hydroxyisoindolinones using aqueous *tert*‐butyl hydroperoxide (TBHP) as an oxidant (Scheme 5 A). The group of Zhang^[29]^ described a similar methodology, but in this context, the aldehydes utilized in the reaction were generated by in situ oxidation of the toluene derivatives (Scheme 5 B). This methodology was further implemented to functionalization of 4*H*‐Benzo[*d*][1,3]oxazin‐4‐ones to fused tetracyclic compounds containing the isoindolinone moiety by Kumar et al.^[30]^ (Scheme 5 C).

**Scheme 6:**
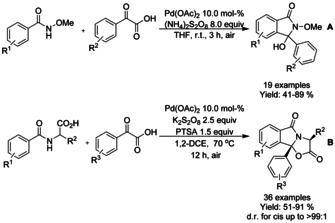
Decarboxylative strategies for synthesis of isoindolinones via palladium catalyzed C−H functionalization.

Both Li^[31]^ and Wang^[32]^ utilized a decarboxylation based methodology for the synthesis of 3‐hydroxy‐isoindolinone and fused tricyclic isoindolinone derivatives, respectively (Scheme 6 A and B). Both methodologies were applied for broad range of substrates and were able to proceed under air utilizing persulfates as oxidants. In general, substituents at *meta*‐position (Scheme 6. R^1^, R^2^ or R^3^) resulted in diminished yields.

### Rhodium catalyzed C−H functionalization

#### Rh‐catalyzed olefination/alkylation‐cyclization

**Scheme 7:**
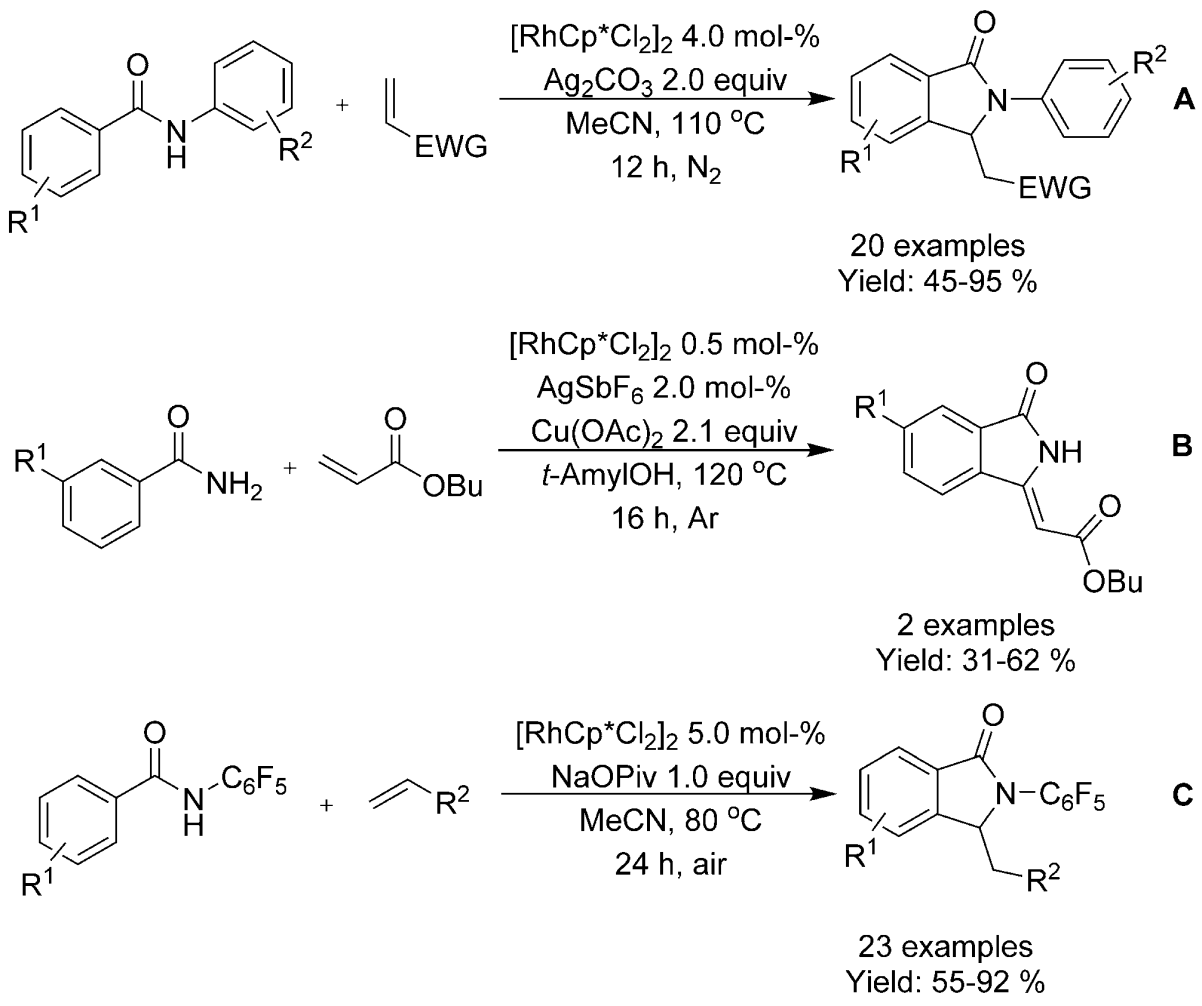
Rh^III^‐catalyzed aryl C−H olefination reactions.

Pentamethylcyclopentadienyl rhodium dichloride dimer ([RhCp*Cl_2_]_2_) has found widespread utility in the synthesis of diversely substituted isoindolinones via the Rh^III^‐catalyzed aryl C−H olefination reactions. Both Li^[33]^ and Glorius^[34]^ described an oxidative C−H olefination using unactivated or underivatized benzamides. Both methods proceed with electron deficient olefins in the presence of a suitable oxidant, such as silver carbonate or copper(II) acetate (Scheme 7; Li: **A** and Glorius: **B**). Instead of stoichiometric oxidants, Lu et al.^[35]^ were able to utilize the oxygen present in air to synthesize 3‐substituted isoindolinones from electron deficient olefins and *N*‐pentafluorobenzene derivatized benzamides (Scheme 7 C).

**Scheme 8:**
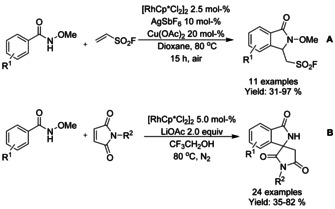
Coupling of methoxy substituted benzamides with alkenes.

Qin^[36]^ used an oxidative coupling strategy to provide access to ethane sulfonylfluoride derivatized isoindolinones from *N*‐methoxy substituted benzamides and ethene sulfonylfluoride (Scheme 8 A). The sulfonyl fluoride group can be readily utilized for example in a sulfur(VI) fluoride exchange reaction, which is a type of click reaction. Jeganmohan^[37]^ conveyed their previous work with cobalt catalyzed isoindolinone synthesis (vide infra) to similar Rh^III^ catalyzed method. Using maleimides as substrates with *N*‐methoxy‐benzamides, 3‐spirosubstituted isoindolinones were produced in a facile manner (Scheme 8 B).

**Scheme 9:**
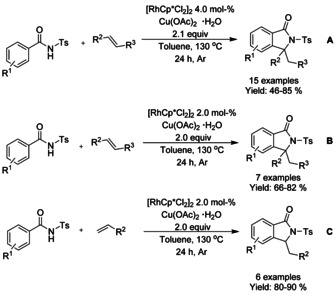
Synthesis of isoindolinones from *N*‐tosyl‐derivatized benzamides.

Tosyl derivatized benzamides were successfully used in C−H olefination^[38a]^ providing a direct route to 3,3‐disubstituted‐isoindolinones (Scheme 9 A). Initially, in addition to the *N*‐tosyl benzamides, only 1,2‐homodisubstituted activated alkenes were employed as reactants (Scheme 9 B, **C**) but later^[38b]^ it was further optimized and expanded to terminal alkenes. The yield of these reactions was mainly dependent of electron withdrawing substituents on the alkene.

**Scheme 10:**
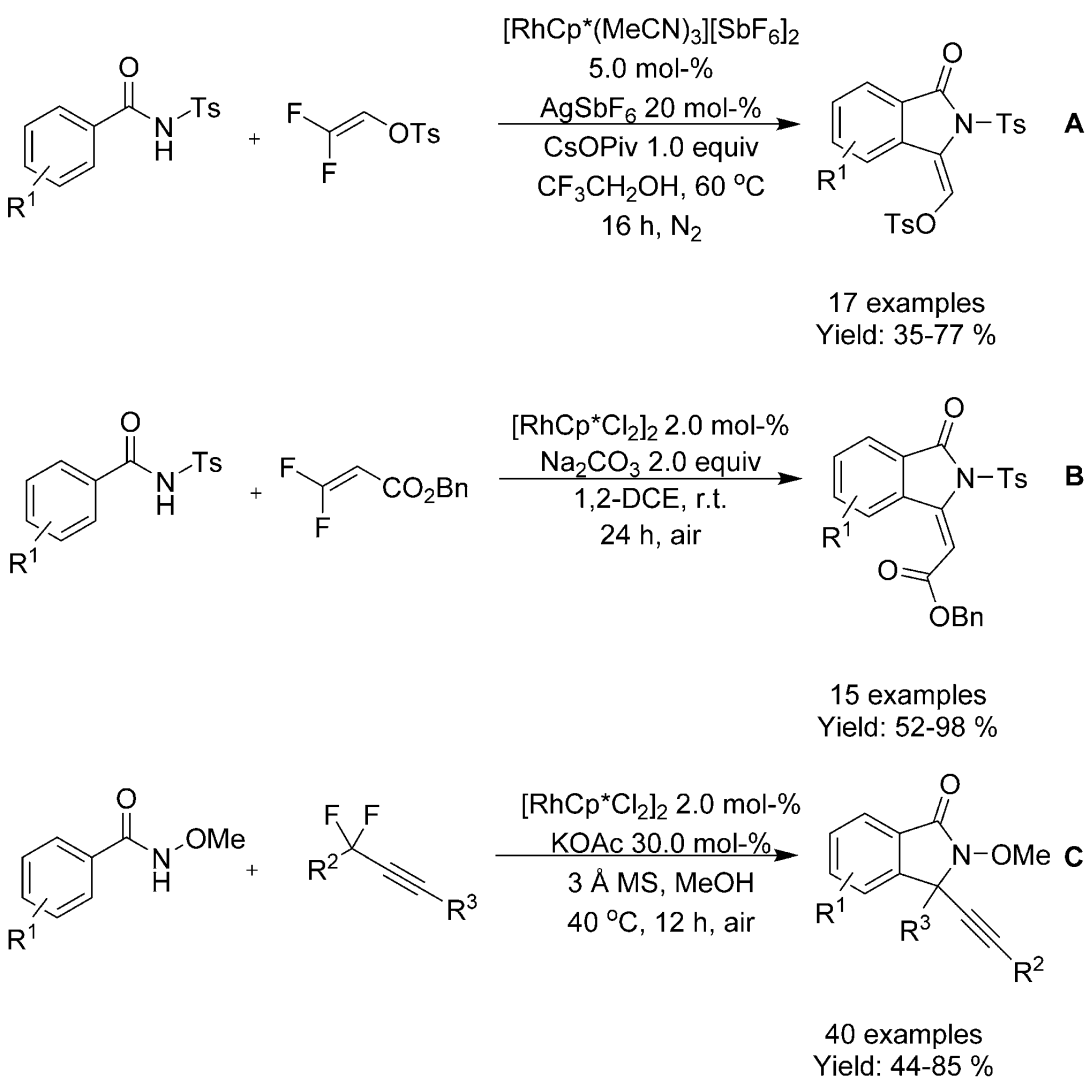
Rhodium catalyzed C−H functionalization combined with C−F cleavage.

The combination of C−H and C−F bond functionalization utilizing redox neutral bimetallic Rh^III^/Ag^I^ relay catalysis was described by Wang (Scheme 10 A).^[39]^ Here, rhodium was used for C−H functionalization and silver for the defluorinative cyclization to afford 3‐alkylidene isoindolinones, which could further be derivatized for example to aristolactam BII. Similarly Feng and Loh^[40]^ established a purely rhodium catalyzed synthesis of 3‐alkylidene isoindolinones from benzyl *gem*‐difluoroacrylate and *N*‐tosyl‐benzamides at room temperature in the presence of an inorganic base and without the need of protective gases (Scheme 10 B). The combination of C−H activation and C−F cleavage was further expanded to g*em*‐difluoromethylene alkynes with *N*‐methoxy‐benzamide substrates.^[41]^ It is noteworthy that a reaction proceeded in the non‐halogenated solvent under air and tolerated a broad range of substrates (Scheme 10 C).

**Scheme 11:**
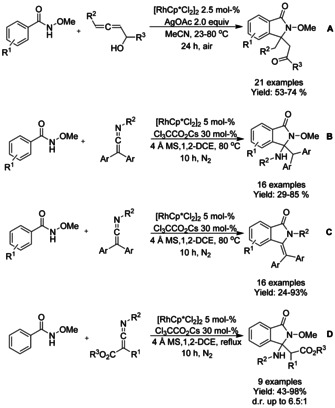
Rhodium catalyzed coupling of α‐allenols and ketimines to *N*‐methoxy‐benzamide derivatives.

Liu and Lu^[42]^ described the coupling of α‐allenols and *N*‐methoxy‐benzamide derivatives in the presence of a rhodium catalyst and silver acetate as the external oxidant, forming 3,3‐disubtituted isoindolinones (Scheme 11 A). In their experiments, it was found that the hydroxyl group, has a significant effect on the chemo‐ and regioselectivity. Similarly, ketimines have been investigated as coupling partners for *N*‐methoxy‐benzamide derivatives. Lu and Wang^[43]^ described C−H bond activation/annulation cascade forming 3‐aminoisoindolinones and 3‐diarylmethyleneisoindolinones depending on the substituents on the benzamide (Scheme 11 B and C). For example, *N*‐methoxy‐1‐naphthamides and benzamides bearing bulky substituents at *ortho*‐position would lead to 3‐diarylmethyleneisoindolinones. Later the diastereoselectivity of this cascade to 3‐aminoisoindolinones was also investigated (Scheme 11 D).^[44]^

**Scheme 12:**
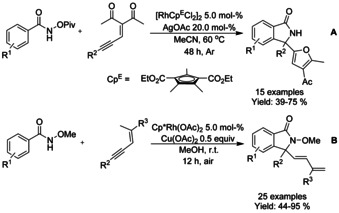
Enyne derivatives in the synthesis on isoindolinones.

Similarly to allene derivatives, enynes have been successfully coupled with benzohydroxamic acid or *N*‐methoxy‐benzamide derivatives forming 3,3‐disubstituted isoindolinones. Chang^[45]^ discovered that replacing two methyl groups from Cp* ligand for carboxylic acid ethyl ester would significantly tune the catalyst towards formal [4+1] annulation (Scheme 12 A). By treating *N*‐pivaloyloxy benzamide with conjugated enynones, a number of isoindolinones could be synthesized with furan substituents on the position C3. An oxidative annulation between enynes and *N*‐methoxybenzamides was described by Li (Scheme 12 B).^[46]^ Here, combination of copper(II) acetate and air was employed as oxidants in methanol at room temperature forming a variety of 3,3‐disubstituted isoindolinones at good to excellent yields.

**Scheme 13:**
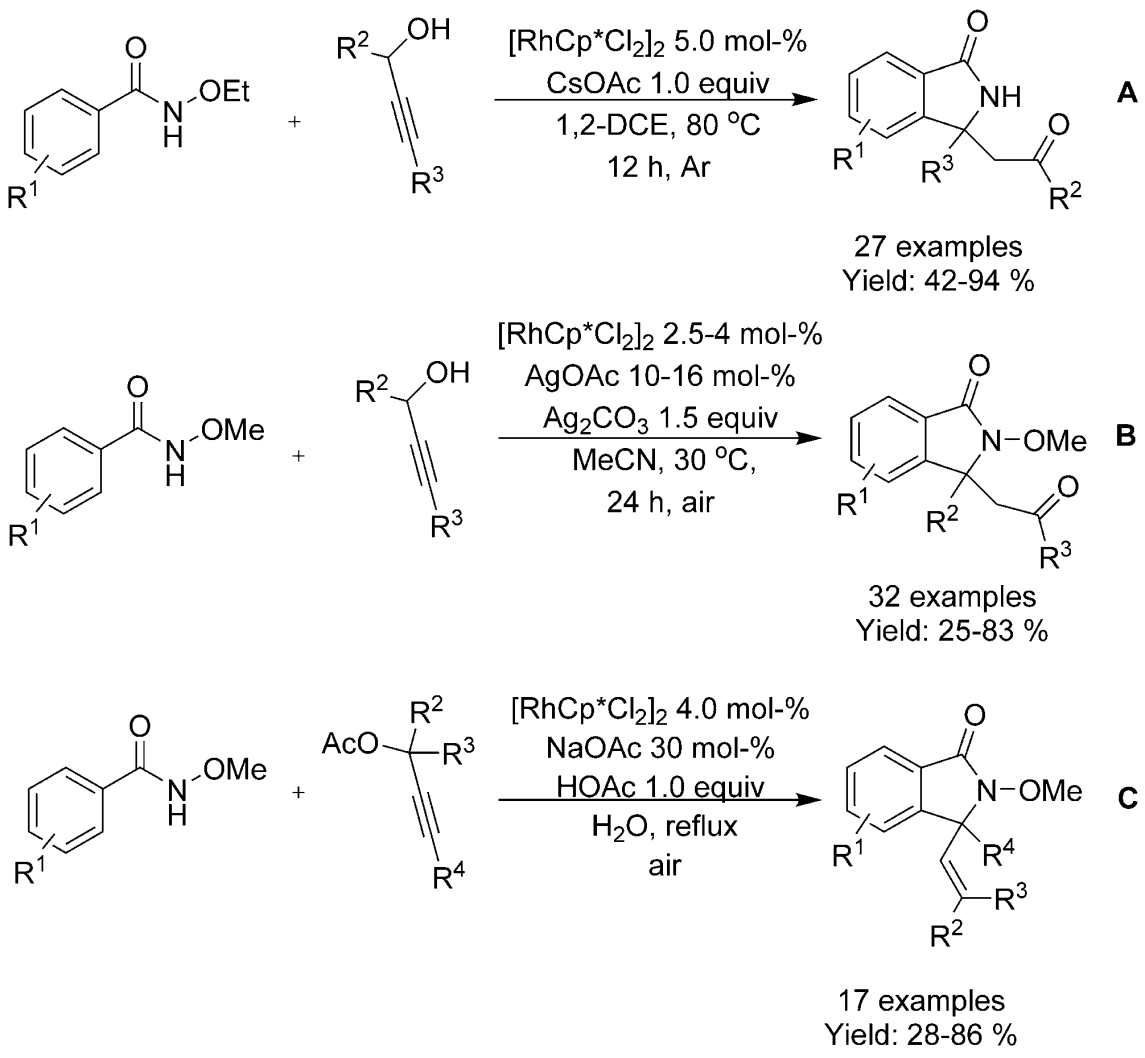
Propargyl alcohols and propargyl acetates as C_1_ synthons.

Propargyl alcohols and propargyl acetates have found utility as C_1_ synthons in the preparation of isoindolinone derivatives. Liu^[47]^ described the coupling of propargyl alcohol derivatives and *N*‐ethoxy benzamides with cesium acetate in 1,2‐dichloroethane at elevated temperatures under argon leading to a broad variety of isoindolinones (Scheme 13 A). The ethoxy group utilized as directing group was also removed during the course of the reaction limiting the need for subsequent synthetic operations. Li^[48]^ was able to synthesize similar compounds under air at close to ambient conditions in the presence of silver acetate and silver carbonate (Scheme 13 B). Ma^[49]^ presented an approach to isoindolinone synthesis utilizing propargyl acetates as C_1_ synthons in aqueous media in the presence of sodium acetate and acetic acid at reflux temperature. Based on experimental evidence, it was proposed that the reaction proceeds via allene intermediate formation followed by cyclization, whereas in the case of propargyl alcohol reactants the reaction would proceed via π‐allylic intermediate.

**Scheme 14:**
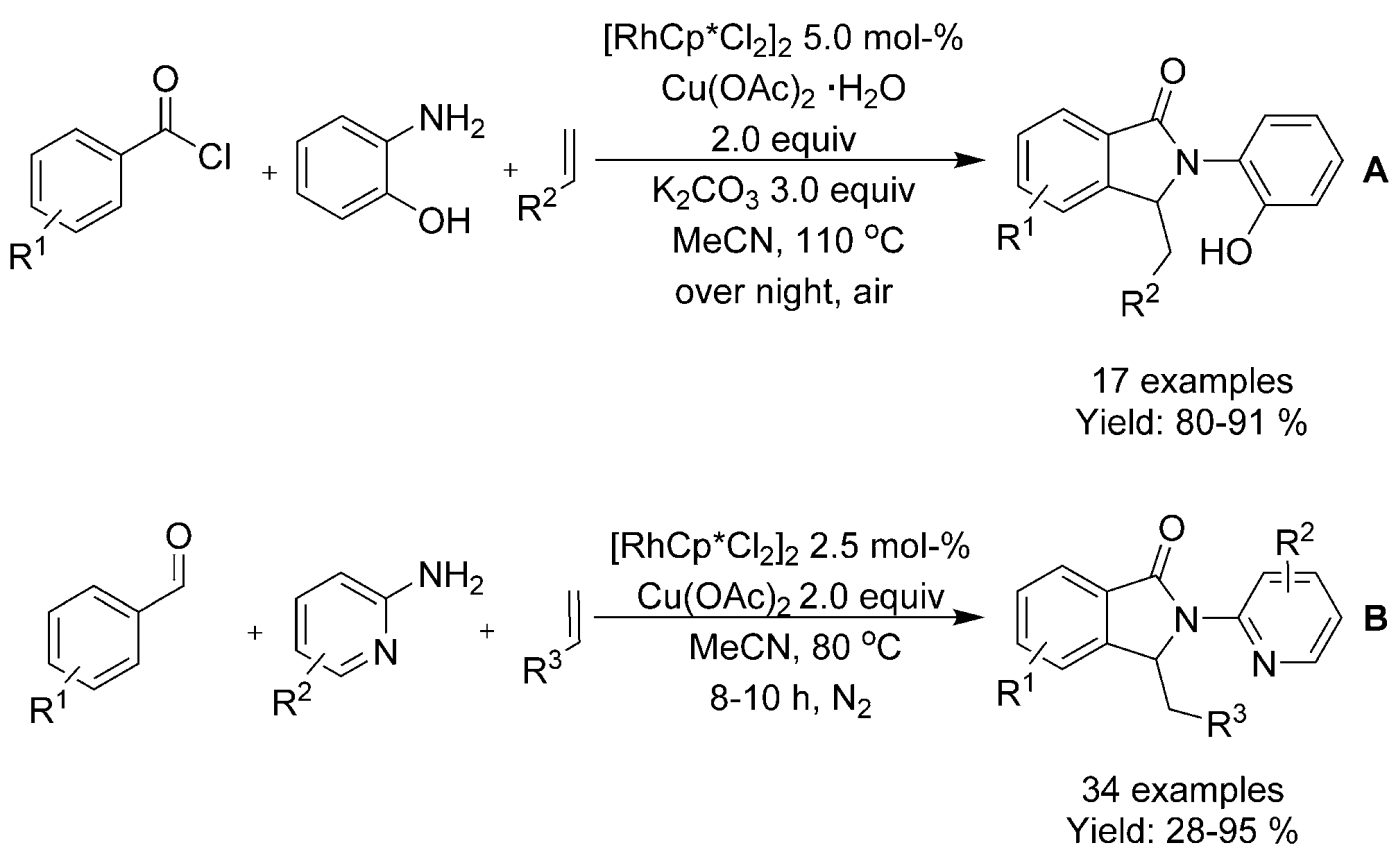
Rhodium catalyzed three‐component synthesis of isoindolinone derivatives.

Rh^III^‐catalyzed three‐component synthesis of isoindolinone frameworks was achieved by both Wang et al.^[50]^ and Zhang et al.^[51]^ Wang used a combination of benzoyl chloride derivative, 2‐aminophenol and electron deficient alkene derivatives to synthesize a variety of substituted isoindolinones in a single step under air (Scheme 14 A). In comparison, Zhang utilized an aromatic aldehyde, 2‐aminopyridines and similarly activated olefins under nitrogen atmosphere, and was able to synthesize pagoclone and pazinaclone in a single step, albeit in 15 % and 20 % isolated yields, respectively (Scheme 14 B). These reactions can be operated using commercially available reactants without the need of significant prior synthetic work. In both cases the amine reactant is selected to function as a directing group for the oxidative rhodium catalyzed C−H activation reaction.

**Scheme 15:**
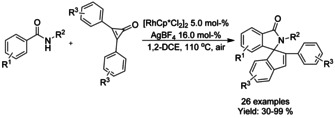
Ketones in rhodium catalyzed synthesis of isoindolinones by C−H bond activation.

Yuan et al.^[52]^ reported a redox‐neutral Rh^III^ catalyzed double C−H bond functionalization cascade to produce 3‐spiro substituted isoindolinones from 2,3‐diarylcyclopropenones and benzamide derivatives (Scheme 15). In this manner, two new C−C bonds and one new C−N bond were formed with the indene substituent at C3 position of the isoindolinone skeleton. While this reaction does require elevated temperature and halogenated solvent, it does not need protection from air.

#### Rh‐catalyzed isocyanide insertion‐cyclization

**Scheme 16:**
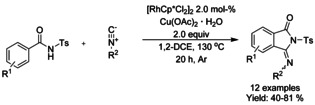
Synthesis of isoindolinones from *N*‐tosyl‐derivatized benzamides and isocyanides.

Zhu reported an oxidative rhodium catalyzed *ortho*‐C−H activation annulation cascade with isocyanides, producing 3‐iminoisoindolinones (Scheme 16).^[53]^ In their work, the main challenges, in terms of yield, arose from fluoro and trifluoromethyl substituted benzamide derivatives.

#### Rh‐catalyzed cyclization using carbene reagents

**Scheme 17:**
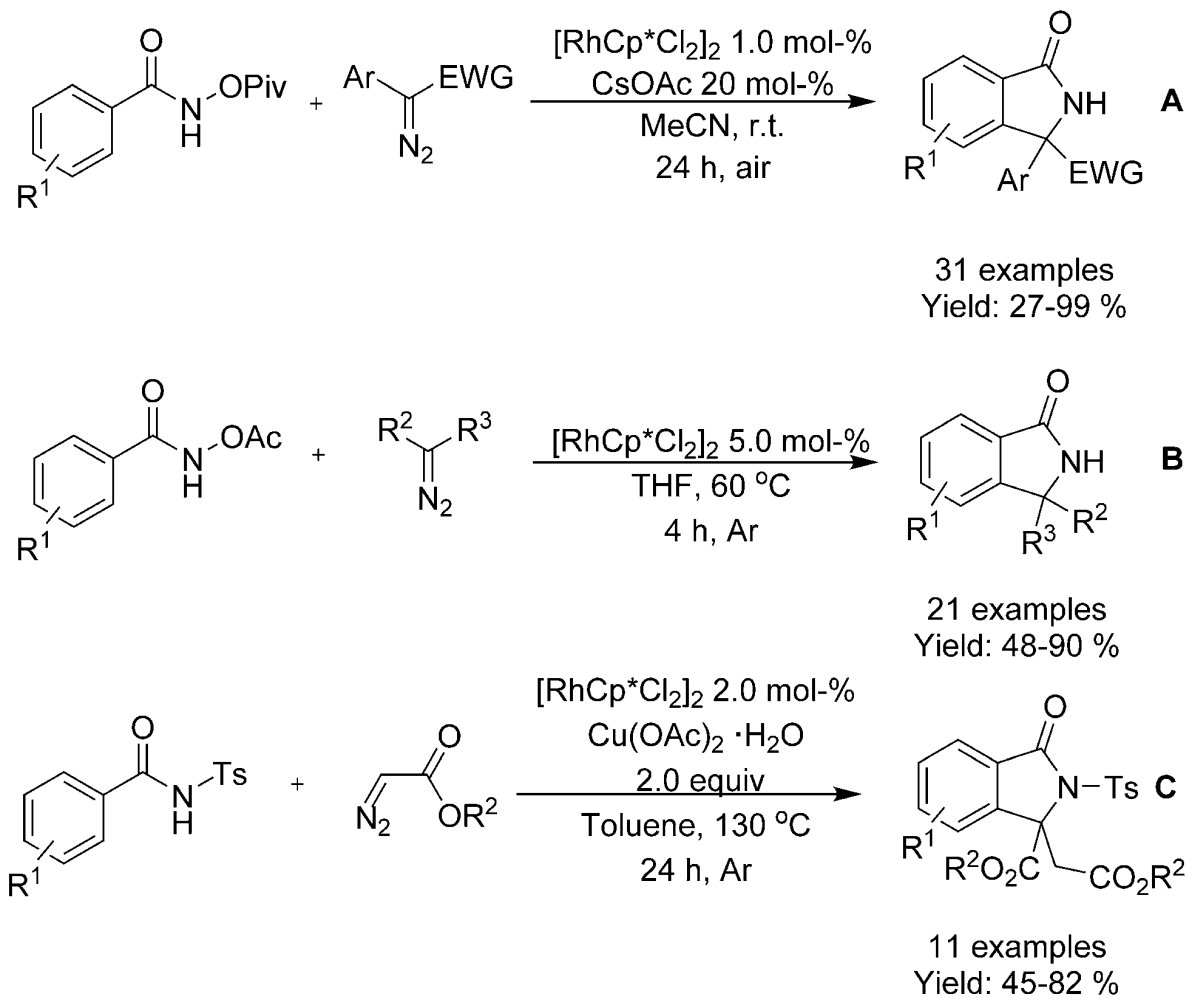
Coupling of diazo compounds with benzohydroxamic acid derivatives and tosylated benzamides.

The coupling of diazo compounds to benzohydroxamic acid derivatives in Rh^III^‐catalyzed formal [4+1] cycloaddition proceeding via C−H bond activation forming isoindolinones was demonstrated by both Rovis^[54]^ and Yu.^[55]^ Rovis utilized *O*‐pivaloyl benzohydroxamic acids with methyl α‐aryldiazoacetates or 1‐aryl‐2,2,2‐trifluoro diazoethanes as carbenoid coupling partners, whereas Yu focused on *O*‐acetyl benzohydroxamic acids with diazomalonates (Scheme 17 A and B, respectively). The method reported by Rovis proceeded in the presence of substoichiometric amount of base under air while Yu did not need additional base but utilized higher temperature under argon atmosphere. In addition of olefination of tosylated benzamides Zhu^[38b]^ extended the their methodology to encompass diazoacetate reactants instead of alkenes (Scheme 17 C). The yield of these reactions was mainly dependent of electron withdrawing substituents on the alkenes or of steric hindrances present in diazoacetate substrates.

**Scheme 18:**
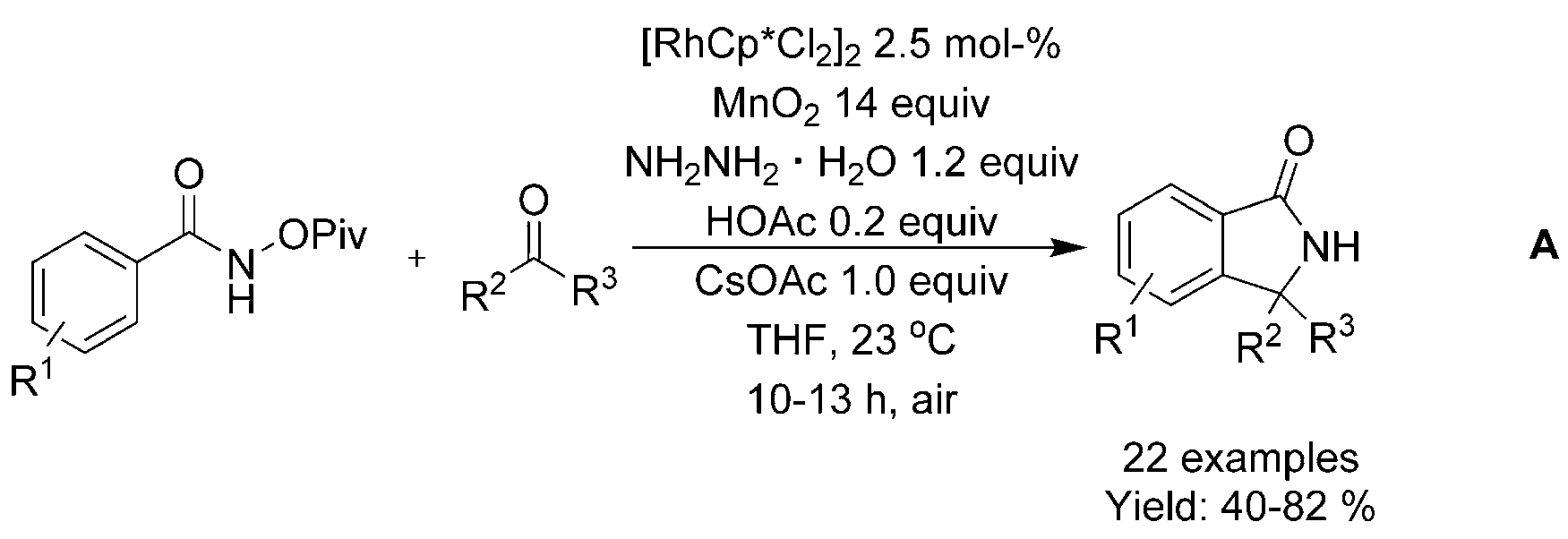
Ketones in rhodium catalyzed synthesis of isoindolinones by C−H bond activation.

Cui^[56]^ reported the synthesis of isoindolinones from benzohydroxamic acids and diazo derivatives, generated in situ from ketones in presence of use of hydrazine and manganese oxide (Scheme 18). While in this manner the need to handle and prepare possibly hazardous diazo compounds is limited, the methodology still utilizes highly toxic hydrazine as well as super stoichiometric amount of manganese oxide.

#### Rh‐catalyzed asymmetric synthesis of isoindolinones

**Scheme 19:**
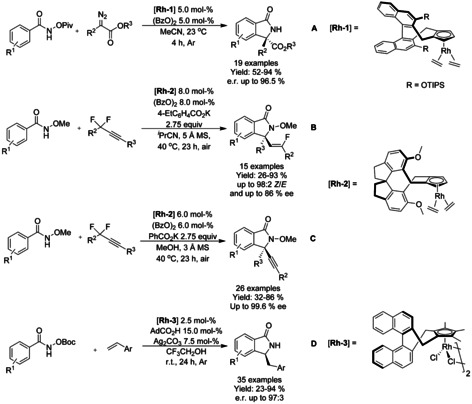
Asymmetric synthesis on isoindolinones via rhodium catalyzed C−H functionalization.

Since the seminal work on asymmetric arylation of *N*‐tosylarylimines by Xu and Lin in 2007,^[57]^ only a few examples of asymmetric synthesis of isoindolinones by transition metal catalyzed transformations have been reported. In case of asymmetric rhodium catalyzed C−H functionalization reactions, all of the catalysts described share a common feature of bulky axially chiral substituent, such as 1,1’‐binaphtyl or 1,1’‐spirobiindane derivative, at the Cp ring. Cramer^[58]^ was the first to utilize these types of ligands for rhodium catalyzed asymmetric synthesis of isoindolinone via C−H functionalization reaction. The reactions proceeded under mild conditions and allowed to achieve high enantioselectivities without the need of superstoichiometric amount of additives (Scheme 19 A). This methodology was based on previous work of Rovis^[54]^ utilizing donor/acceptor diazo compounds with *O*‐pivaloyl benzhydroxamic acids. Based on the work of Loh,^[41]^ Wang^[59]^ reported a solvent‐dependent asymmetric synthesis of alkynyl and monofluoroalkenyl isoindolinones (Scheme 19 B and C). In isobutyronitrile, the monofluoroalkenyl isoindolinones were formed in moderate to good enantioselectivity with predominance of the *Z* isomer, whereas in methanol the alkynyl derivatives were formed with very high enantioselectivity. You^[60]^ described the first enantioselective [4+1] annulation reaction between *N*‐(*tert*‐butoxycarbonyl)benzamides and alkenes via rhodium catalyzed C−H activation using chiral dimeric rhodium precatalysts (Scheme 19 D). The reaction proceeds under mild conditions and is fully atom economic, requiring only minimal amounts of additives, although the reaction was performed under argon atmosphere and trifluoroethanol as the solvent. The methodology was applied to a broad range of benzamide and styrene derivatives with the only major limitations arising from *ortho*‐substituted styrene derivatives.

### Ruthenium catalyzed C−H functionalization

#### Ru‐catalyzed olefination/alkylation‐cyclization

**Scheme 20:**
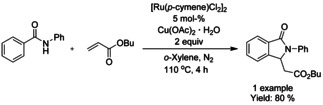
Coupling of benzanilide with butyl acrylate by means of ruthenium catalyzed C−H functionalization.

Similarly to rhodium, a single ruthenium precatalyst, dichloro(*p*‐cymene)ruthenium(II) dimer, has mainly dominated the C−H functionalization reactions. One of the earliest examples was described by Hashimoto et al.^[61]^ coupling benzanilide with butyl acrylate in 80 % yield in the presence of dichloro(*p*‐cymene)ruthenium(II) dimer and copper(II) acetate hydrate as oxidant, illustrated in Scheme 20.

**Scheme 21:**
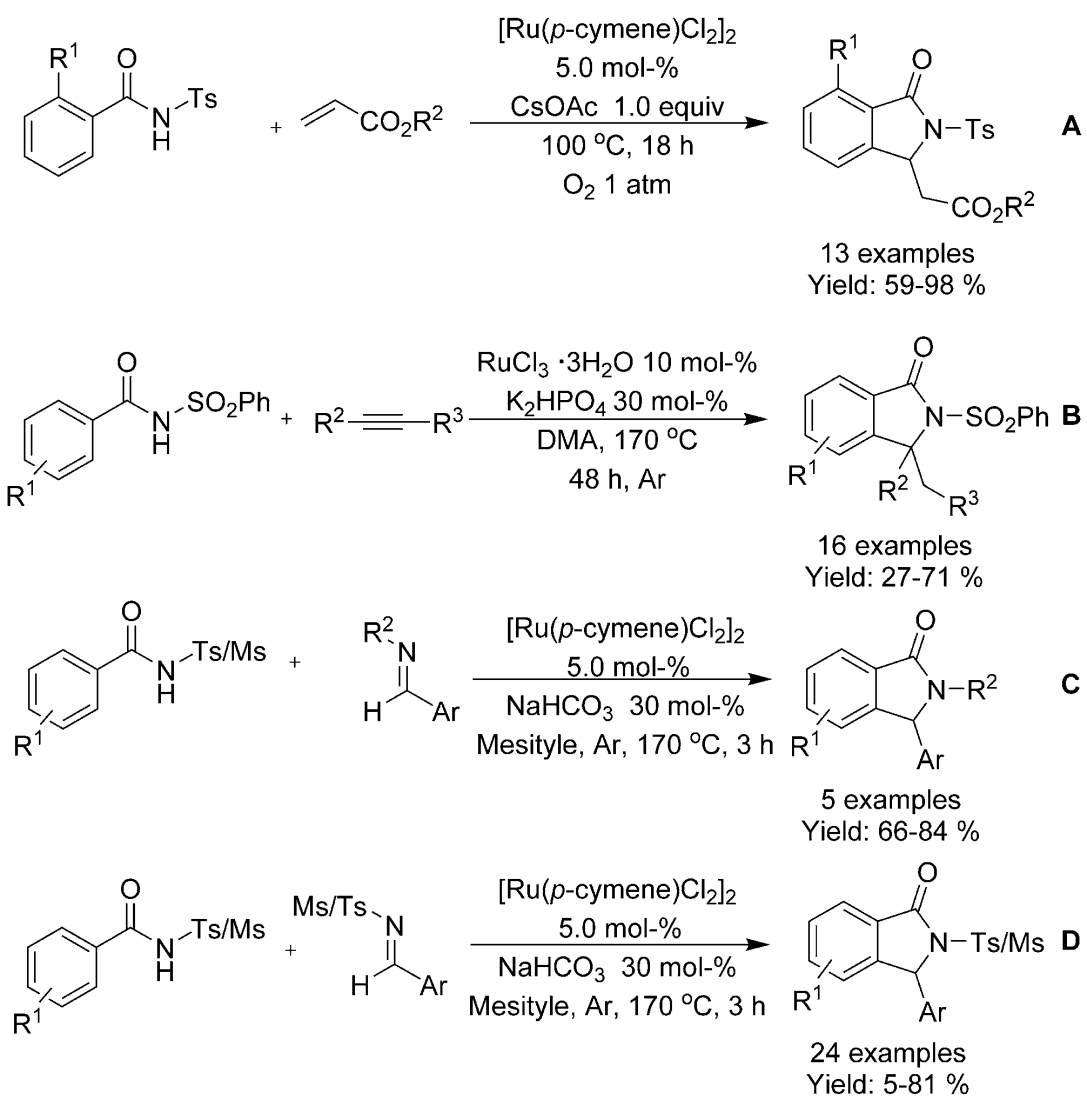
N‐Sulfonated benzamides as substrates in isoindolinone synthesis by means of ruthenium catalyzed C−H functionalization.

N‐Sulfonated benzamides have been successfully utilized as substrates in the ruthenium catalyzed C−H functionalization reactions for the synthesis of 3‐substituted isoindolinones. Ackermann^[62]^ described an oxidative C−H coupling with subsequent aza‐Michael reaction between *ortho*‐substituted *N*‐tosyl‐benzamides and a variety of acrylates (Scheme 21 A). Miura et al.^[63]^ utilized a similar approach to C−H functionalization applied to internal alkynes and N‐sulfonated aromatic amides, followed by base catalyzed intramolecular cyclization (Scheme 21 B). Later the methodology was extended to aldimines^[64]^ as well (Scheme 21 C and D).

**Scheme 22:**
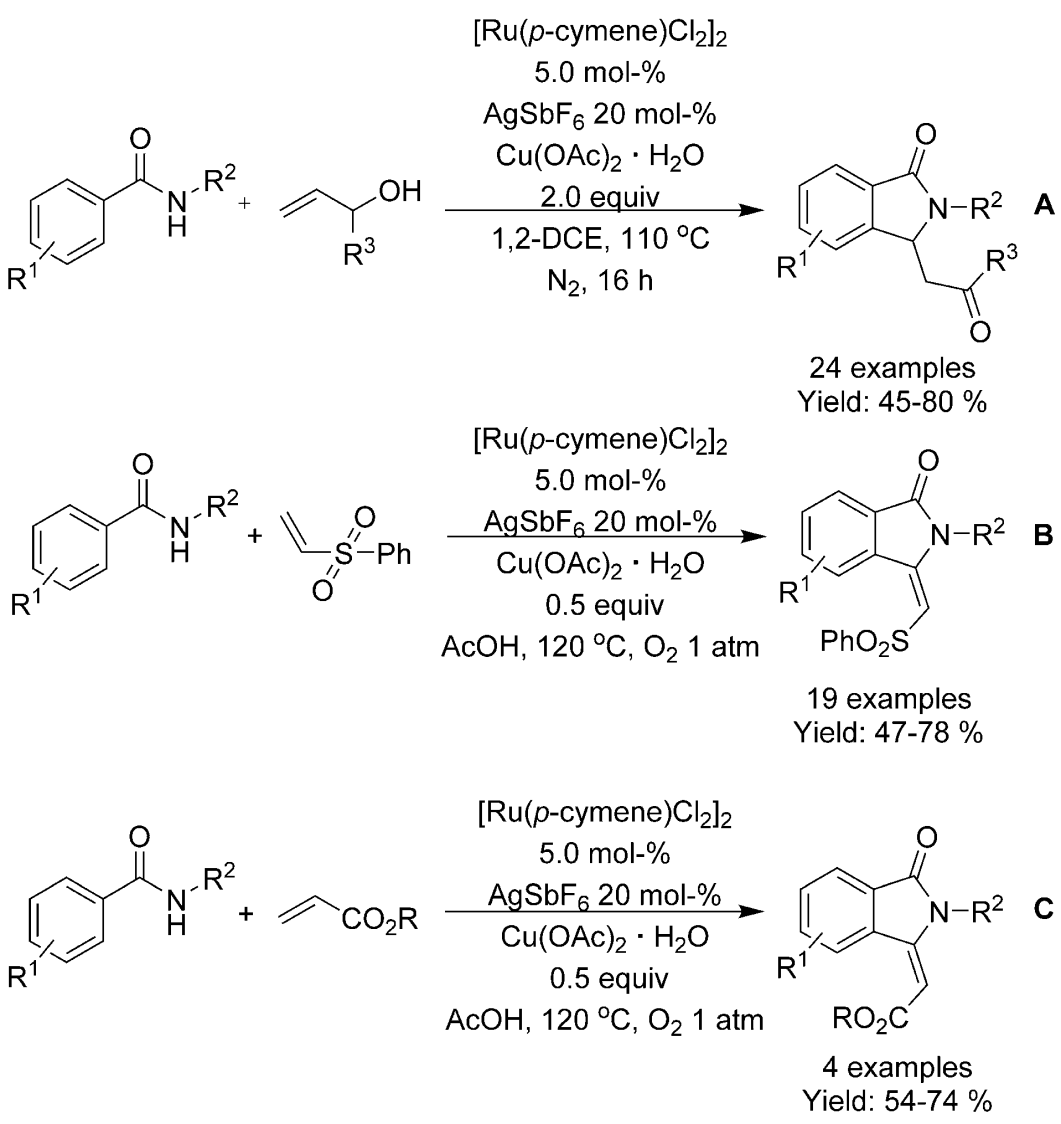
Ruthenium catalyzed coupling of benzamides and olefins to 3‐substituted isoindolinones.

Jeganmohan^[65]^ described the use of allylic alcohols and *N*‐alkyl/aryl benzamides in oxidative C−H coupling/cyclization cascade. N‐Benzylated benzamides proved to be the best substrates under the conditions utilized, providing a broad range of 3‐subtituted isoindolinones (Scheme 22 A). Based on experimental investigations, it was proposed that the allylic alcohols would most likely dehydrogenatively convert into α,β‐unsaturated enones prior to the C−H functionalization and cyclization cascade. Few years later the methodology was expanded to coupling of N‐alkylated benzamides with phenyl vinyl sulfone or acrylate derivatives, producing 3‐alkylidene isoindolinones (Scheme 22 B and C).^[66]^ Furthermore, it was demonstrated that the 3‐(phenylsulfonyl)methylene derivatized isoindolinones could be converted into aristolactams in a single step in good, isolated yields.

**Scheme 23:**
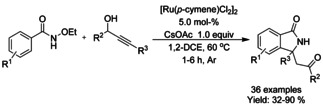
Coupling of propargyl alcohol derivatives with *N*‐ethoxy benzamides.

Similarly to their work with rhodium,^[47]^ Liu^[67]^ reported a ruthenium catalyzed formal [4+1] annulation of propargyl alcohol derivatives with *N*‐ethoxy benzamides by means of C−H functionalization reaction (Scheme 23). In comparison to the rhodium catalyzed method the overall isolated yields are very close to each other and the reaction with the ruthenium catalyst is operated at slightly lower temperature, not to mention the price difference between rhodium and ruthenium complexes. Similar to the work on rhodium, it was proposed that the reaction proceeds via π‐allylic ruthenacycle intermediate.

**Scheme 24:**
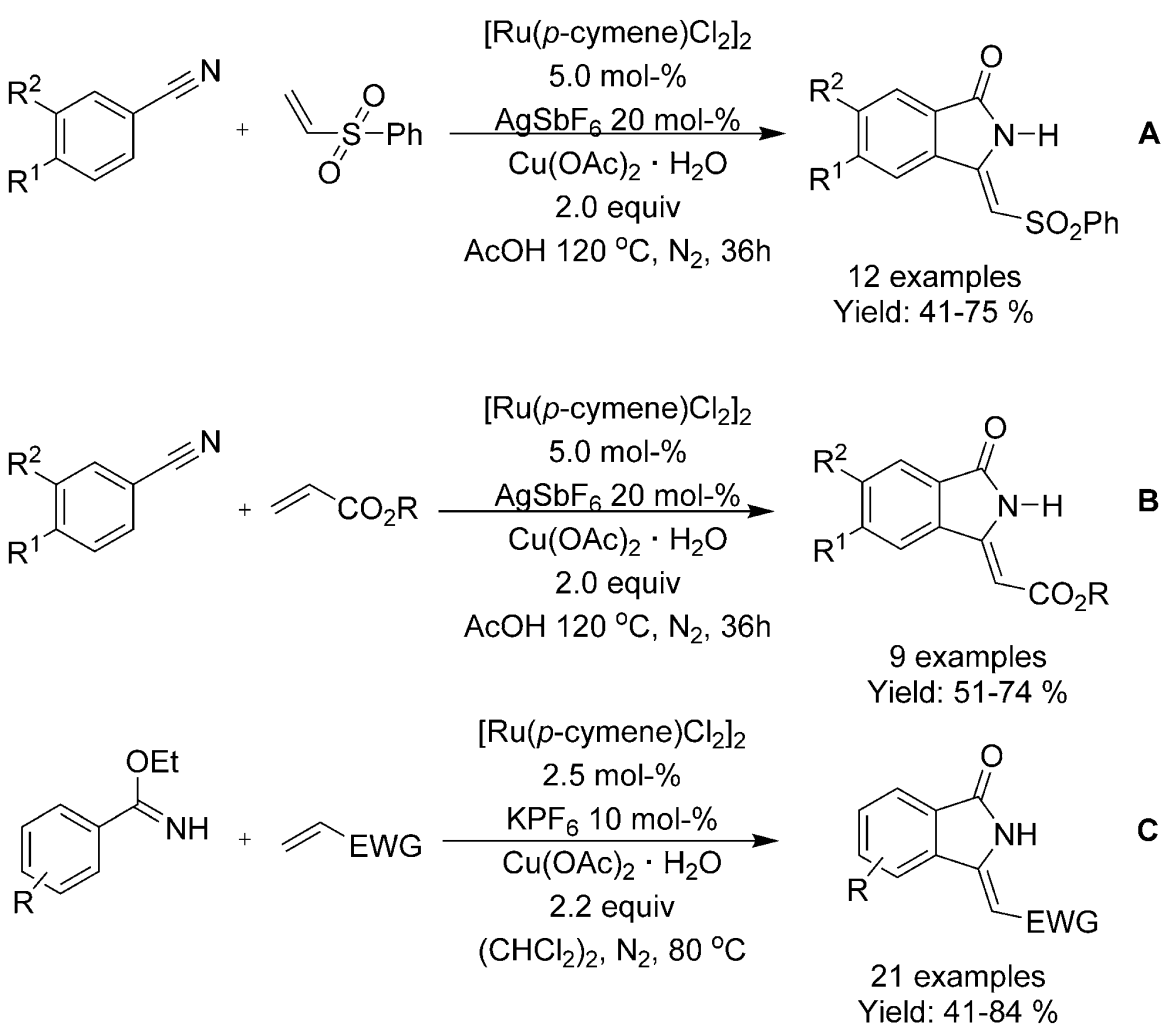
Reagents with unsaturated carbon nitrogen bonds utilized in isoindolinone synthesis.

Jeganmohan^[68]^ demonstrated the utility of dichloro(*p*‐cymene)ruthenium(II) dimer as precatalyst for the cyclization of benzonitriles with phenyl vinyl sulfone or acrylate derivatives producing 3‐alkylidene isoindolinones with very high *Z* selectivity (Scheme 24 A and B). In these reactions, the benzonitrile is oxidized in situ to benzamide by the copper(II) acetate followed by the C−H functionalization and subsequent cyclization. Liu^[69]^ described the use of imidates and electron deficient terminal olefins in the synthesis of various 3‐methyleneisoindolin‐1‐ones (Scheme 24 C). The N−H imidates functioned as a directing group for the oxidative alkenylation/annulation cascade, yielding exclusively the *Z*‐isomer of the 3‐methyleneisoindolin‐1‐one derivatives.

#### Ru‐catalyzed spiroannulation via C−H functionalization

**Scheme 25:**
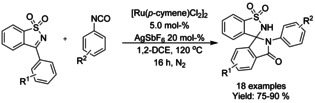
Ru‐catalyzed spiroannulation via C−H functionalization.

The use of 3‐aryl‐*N*‐sulfonyl ketimines and aryl‐isocyanate in spiroannulation to form spiro‐isoindolinone‐benzosultams was established by Subba Reddy (Scheme 25).^[70]^ The methodology produced a variety of spiro‐isoindolinone‐benzosultams in good yields without the excessive need of additives. Based on previous publications, the reaction was proposed to proceed via initial formation of benzamide derivative by C−H functionalization followed by the cyclization.

### Nickel catalyzed C−H functionalization

#### Ni‐catalyzed olefination‐cyclization

**Scheme 26:**
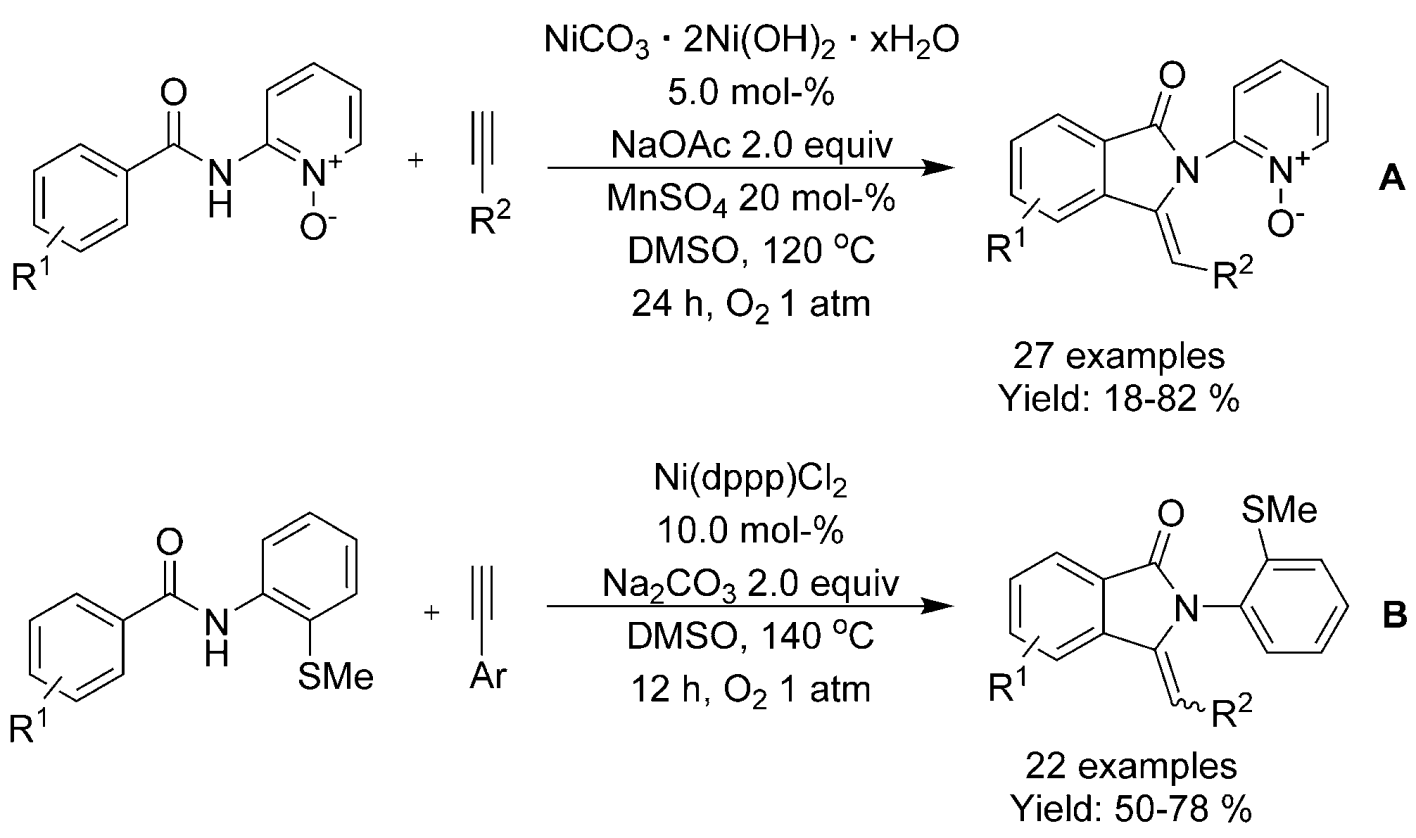
Nickel catalyzed alkenylation/annulation cascade terminal alkenes and benzamides with pendant directing groups.

Both Zheng et al.^[71]^ and Zhang^[72]^ described a nickel catalyzed oxidative C−H alkynylation/annulation cascade with terminal alkynes under oxygen atmosphere yielding 3‐alkylidene isoindolinones in mostly *Z*‐selective manner (Scheme 26 A and B, respectively). The *Z*/*E* selectivity was mainly attributed to the steric hindrance formed by the substituent in both substrates. In both cases, the reaction mechanism is proposed to proceed in identical manner, by initial C−H alkynylation of the aromatic ring, followed by intramolecular annulation to form the isoindolinone.

**Scheme 27:**
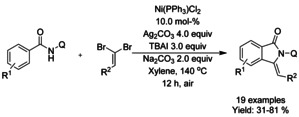
C−H activation and intramolecular annulation cascade between geminal dihalo olefins and *N*‐(quinolin‐8‐yl) benzamides.

Chen^[73]^ used 8‐quinoline as a directing group for nickel(II)/silver(I)‐catalyzed C−H activation and intramolecular annulation cascade (Scheme 27). Geminal dibromoalkenes were utilized to from bromoalkynes in situ with the help of sodium carbonate followed by alkynylation via nickel catalyst. Subsequent cyclization was mediated by silver carbonate and *tert*‐butylammonium iodide forming a number of 3‐alkylidene isoindolinones in moderate to good yields.

#### Ni‐catalyzed isocyanide insertion‐cyclization

**Scheme 28:**
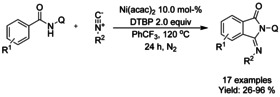
Nickel catalyzed oxidative synthesis of 3‐iminoisoindolinones.

Nickel catalyzed oxidative insertion of isocyanides to *N*‐(quinolin‐8‐yl) benzamides forming 3‐iminoisoindolinones was reported by Lei (Scheme 28).^[74]^ Nickel(II) bis(acetylacetonate) was utilized as the catalyst and di‐*tert*‐butyl peroxide as the oxidant in trifluorotoluene at elevated temperature under nitrogen atmosphere, generating a broad variety of substituted 3‐iminoisoindolinones in poor to good yields. Only iodo‐derivatized benzamides were reported to afford poor yields.

### Copper catalyzed C−H functionalization

#### Cu‐catalyzed olefination/alkylation‐cyclization

**Scheme 29:**
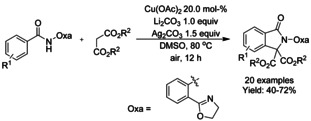
Copper(II) acetate catalyzed aromatic C−H bond coupling to malonates.

Wang et al.^[75]^ described a copper(II) acetate catalyzed aromatic C−H bond coupling of a number of oxazoline protected benzamides and malonates with a subsequent intramolecular ring closure to form 3,3‐disubstituted isoindolinones under air (Scheme 29). The methodology was found to be very specific to malonates as the use of 3‐oxobutanoate afforded 1‐oxo‐1*H*‐isochromene derivative.

**Scheme 30:**
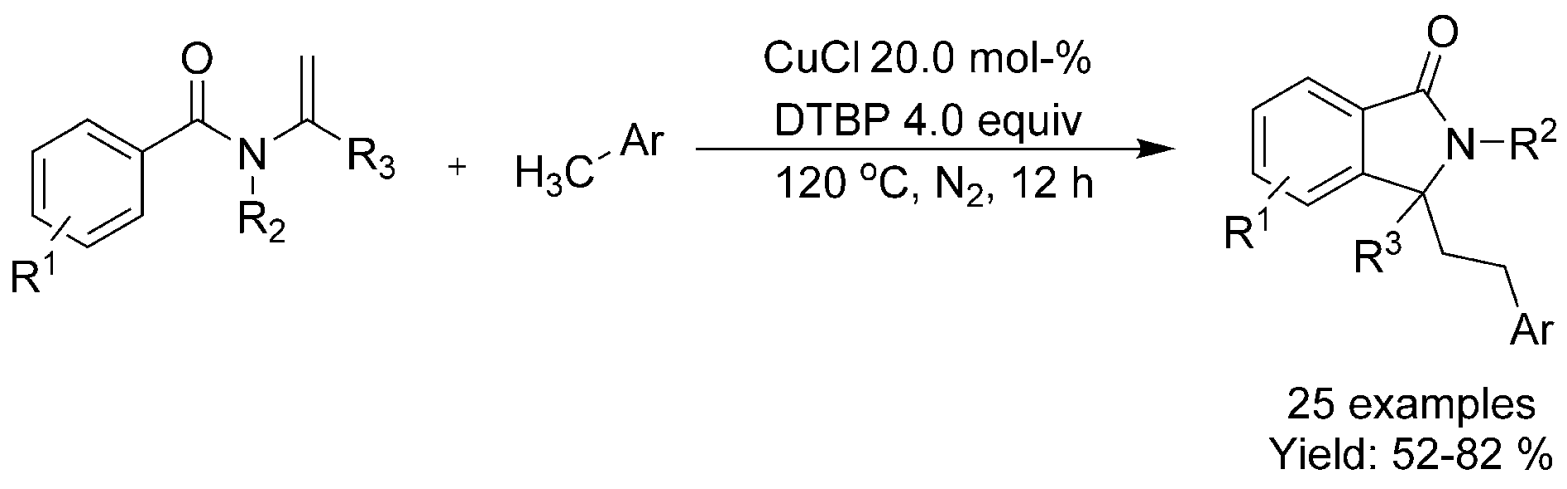
Copper(I) catalyzed radical benzylation and cyclization of tertiary enamides.

Yu^[76]^ established a copper(I) catalyzed radical benzylation and cyclization of tertiary enamides generating a variety 3,3‐disubstituted isoindolinone derivatives in moderate to good yields. In this manner, two new C−C bonds are generated in successive fashion (Scheme 30). While the detailed reaction mechanism is not yet fully clarified, it was postulated that the catalyst, copper(I) chloride, in combination with an oxidant, di‐*tert*‐butyl peroxide, generates a benzyl radical which will initiate the C−C bond forming cascade together with the utilized tertiary enamides.

#### Cu‐catalyzed isocyanide insertion‐cyclization

**Scheme 31:**
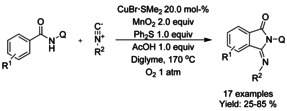
Copper‐catalyzed formal [4+1] cycloaddition of *N*‐(quinolin‐8‐yl) benzamides and isocyanides to 3‐iminoisoindolinones.

Takamatsu et al.^[77]^ developed the first copper‐catalyzed formal [4+1] cycloaddition of *N*‐(quinolin‐8‐yl) benzamides and isocyanides via C−H bond functionalization (Scheme 31). While the reaction is catalyzed by, generally, low‐cost copper(I) bromide dimethyl sulfide complex, a significant amount oxidant (MnO_2_ under oxygen atmosphere) and additive (Ph_2_S) are required for the reaction to proceed as well as reaction temperature of 170 °C.

### Cobalt catalyzed C−H functionalization

#### Co‐catalyzed olefination/alkylation‐cyclization

**Scheme 32:**
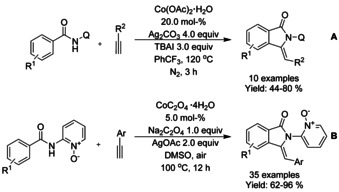
Cobalt catalyzed C−H alkynylation/annulation cascade generating isoindolinones.

Both J. Zhang et al.^[78]^ and L.‐B. Zhang et al.^[79]^ reported very similar cobalt catalyzed C−H functionalization based methods applied to isoindolinone synthesis. J. Zhang investigated a more general method for γ‐lactam synthesis and utilized it to isoindolinones (Scheme 32 A), whereas L.‐B. Zhang strictly focused on isoindolinones (Scheme 32 B). J. Zhang utilized cobalt(II) acetate as a catalyst for *N*‐(quinolin‐8‐yl) benzamides and alkynes, whereas L.‐B. Zhang applied cobalt(II) oxalate for benzamides with *N*‐2‐aminopyridine 1‐oxide auxiliary. Based on experimental evidence, both authors presented a similar reaction mechanism where the terminal alkyne is activated with silver reagent followed by *ortho*‐aromatic‐C−H‐alkynylation and subsequent intramolecular annulation.

**Scheme 33:**
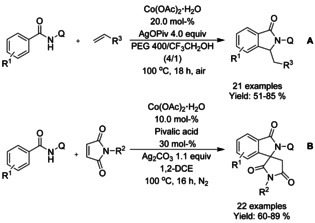
Cobalt(II) acetate catalyzed C−H olefination/annulation cascade.

Ackermann^[80]^ demonstrated the use cobalt(II) acetate in a C−H olefination/annulation cascade with *N*‐(quinolin‐8‐yl) benzamides and acrylates as substrates (Scheme 33 A). Later, Jeganmohan^[81]^ applied somewhat similar reaction conditions for *N*‐(quinolin‐8‐yl) benzamides and maleimides as substrates generating 3‐spirosubstituted isoindolinones (Scheme 33 B). Where Ackermann applied a mixture of PEG 400 and trifluoroethanol as the solvent and silver pivalate as the oxidant, Jeganmohan utilized 1,2‐dichloroethane as the solvent with silver carbonate as the oxidant and a small amount of pivalic acid as additive to improve the reaction yields. Both proposed very similar reaction mechanisms for the initial C−H alkenylation, followed by cyclization either as separate reaction (Ackermann) or as a part of the same catalytic cycle (Jeganmohan).

**Scheme 34:**
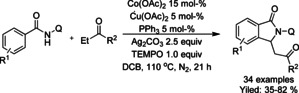
Ketones in cobalt catalyzed C−H functionalization.

Zhao^[82]^ described the use of Co^II^ and Cu^II^ as catalysts for oxidative C−H/N−H functionalization of benzamides with ketones (Scheme 34). Here, the copper(II) catalyst is utilized in dehydrogenation of the ketone with the help of (2,2,6,6‐tetramethylpiperidin‐1‐yl)oxyl (TEMPO) and Ag_2_CO_3_ to form an α,β‐unsaturated ketone, which the is utilized in the cobalt catalyzed C−H functionalization followed by annulation. The methodology was applied to a broad range of *N*‐(quinolin‐8‐yl) benzamide derivatives and a variety of ethyl aryl ketones.

#### Co‐catalyzed cyclization using carbene reagents

**Scheme 35:**
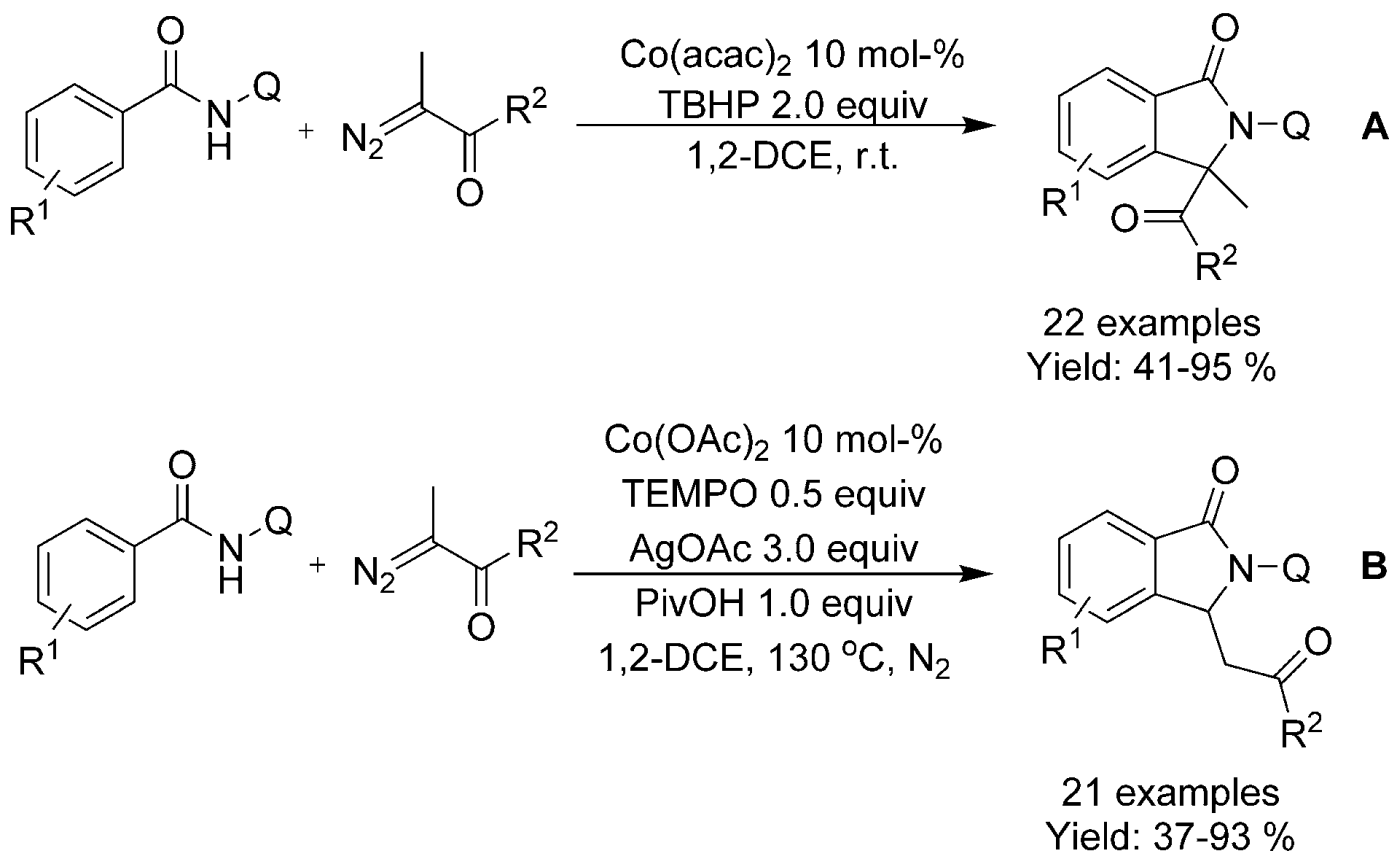
α‐Diazoketones for cobalt catalyzed C−H functionalization.

Li^[83]^ applied α‐diazoketones as substrates for cobalt catalyzed oxidative C−H functionalization cyclization cascade. Depending on the choice of cobalt catalyst and oxidants, the reaction could be directed to form either 3‐mono‐ or 3,3‐disubtituted isoindolinones in moderate to excellent yields (Scheme 35 A and B).

#### Co‐catalyzed isocyanide insertion‐cyclization

**Scheme 36:**
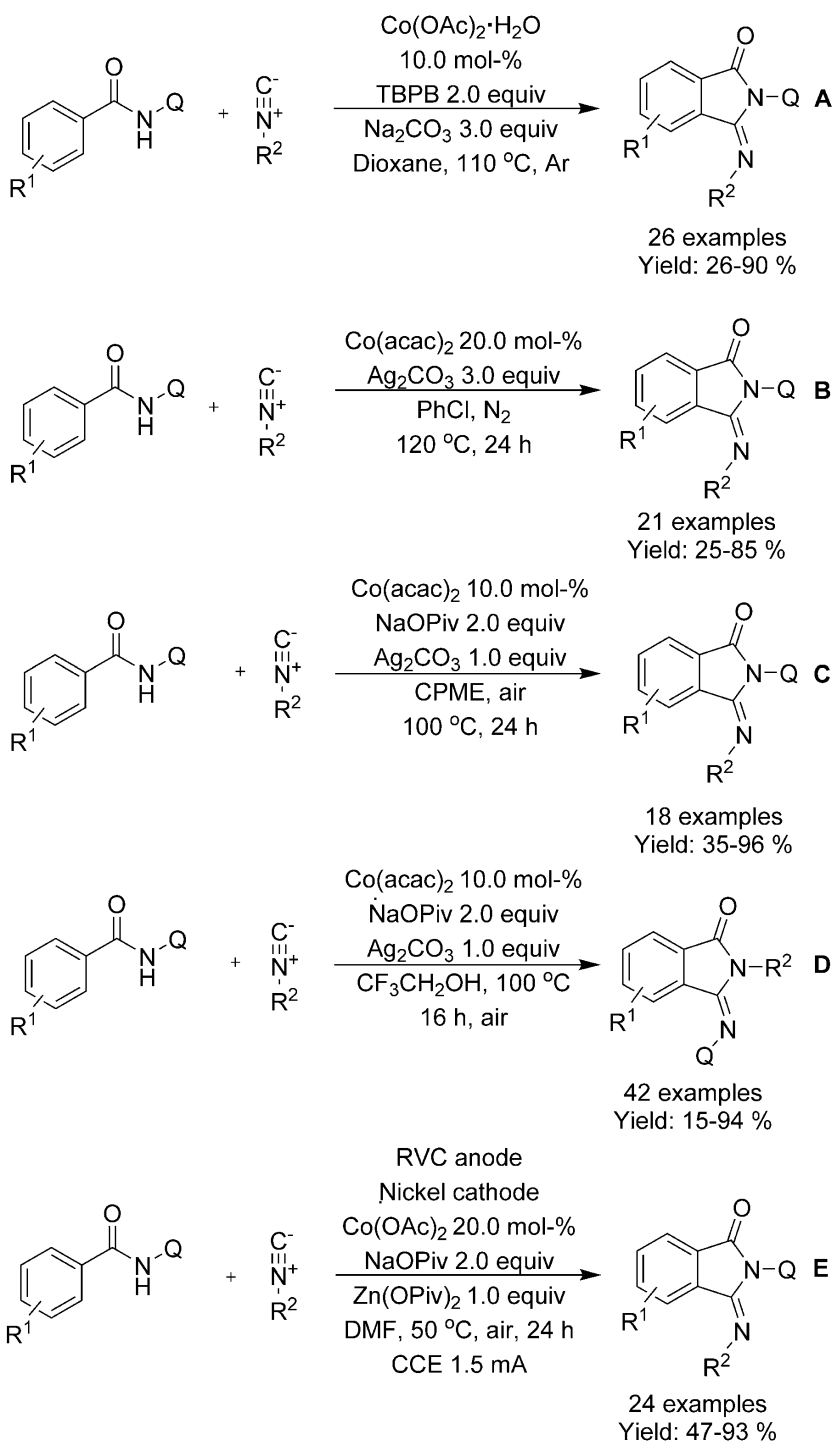
Cobalt catalyzed synthesis of 3‐imino isosindolinones from benzamides and isocyanides.

A variety of cobalt catalyzed methodologies for synthesis of 3‐iminoisosindolinones have been published during the past ten years, with a common feature in the use of 8‐quinolinyl (Q) directing group. Gu et al.^[84]^ described the use of oxidative C−H functionalization/annulation cascade with cobalt(II) acetate as the catalyst and *tert*‐butyl peroxybenzoate as the oxidant (Scheme 36 A), followed by Hao^[85]^ and Sundararaju^[86]^ using cobalt(II) acetyl acetonate and silver salt as the oxidant (Scheme 36 B and C, respectively). Later, Sundararaju^[87]^ reported a general site‐selective cobalt‐catalyzed insertion of isocyanides to form aryl amides through C−H bond activation and alcohol assisted intramolecular *trans*‐amidation with a significantly broad substrate scope (Scheme 36 D). Chen et al.^[88]^ presented a mild cobalt catalyzed C−H functionalization/annulations cascade using electrochemical oxidation in an undivided cell equipped with a reticulated vitreous carbon (RVC) anode and nickel cathode (Scheme 36 E). In their work, electricity was utilized as the oxidant removing the need for stoichiometric oxidants but still stoichiometric amounts of pivalate salts were required as additives. In generalization for all above‐mentioned reactions, benzamides bearing steric bulk near the C−H functionalization site or benzamides with nitro‐substituents are more likely to result in poor yields.

**Scheme 37:**
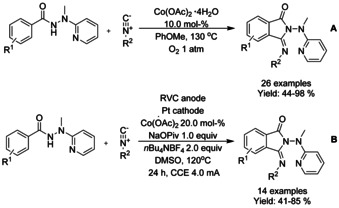
Cobalt catalyzed C−H functionalization/annulation cascade of benzamides with isocyanides.

Similarly to 8‐quinolinyl, 2‐(1‐methylhydrazinyl)pyridine has been utilized as a directing group in cobalt catalyzed C−H functionalization/annulation cascade of benzamides with isocyanides. Zhai^[89]^ described an additive free cobalt‐catalyzed formal [4+1] cycloaddition protocol to 3‐iminoindolinones in anisole at elevated temperature utilizing oxygen as the oxidant (Scheme 37 A). This method proved to be quite robust, efficiently yielding a variety of 3‐iminoisoindolinones mostly in very good, isolated yields. Ackermann^[90]^ also applied the 2‐(1‐methylhydrazinyl)pyridine derivatized benzamides for electrocatalytic oxidative C−H activation/annulation cascade without the need to for external stoichiometric oxidant but still a significantly elevated temperature is required (Scheme 37 B).

## Cross‐Coupling: Annulation/Cyclization

Transition metal catalyzed coupling reactions are among the most efficient methods available for forming carbon−carbon bonds.^[91]^ To date, a variety of effective coupling processes, such as intramolecular Heck or Sonogashira reactions, have allowed the preparation of isoinolinone scaffolds and this field is dominated by palladium‐ and copper‐catalyzed reactions. In this section we discuss the developments in this area, focusing on reactions involving C−C bond formation in cyclization/annulation processes. The following section is categorized by the metal used and subcategorized by the type of isoindolinone derivative obtained as product.

### Palladium‐catalyzed coupling reactions

#### Pd‐catalyzed olefination/alkylation/arylation‐cyclization

**Scheme 38:**
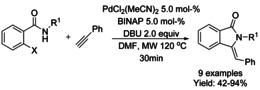
Microwave assisted Pd/BINAP catalyzed reaction by Hellal and Cuny.

In 2011, a microwave assisted Pd/BINAP catalyzed copper‐free Sonogashira coupling 5‐*exo*–*dig* cycloisomerization domino reaction to construct 3‐(phenylmethylene) isoindolinones starting from 2‐halobenzamides and alkynes was reported by Hellal and Cuny. This reaction is compatible with both electron donating and electron withdrawing substituents (Scheme 38).^[92]^

**Scheme 39:**
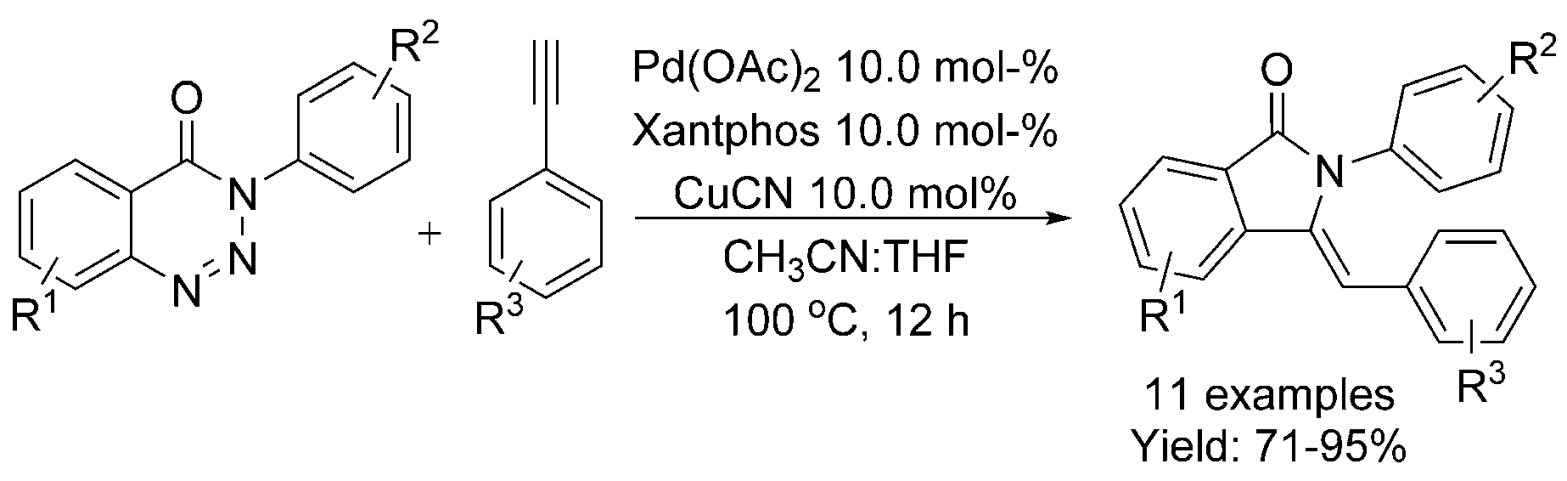
Tandem alkynylation/cyclization reaction to access functionalized (*Z*)‐3‐benzylidene‐isoindolinones.

A highly stereoselective Pd catalyzed strategy to access (*Z*)‐3‐benzylidene‐isoindolinones via denitrogenative tandem alkynylation/cyclization reaction of 1,2,3‐benzotriazinones with aromatic terminal alkynes was described by Mannathan (Scheme 39).^[93]^ This reaction proceeds in the presence of copper cyanide (CuCN) which showed to be essential for the cyclomerization process together with Pd‐catalyst.

**Scheme 40:**
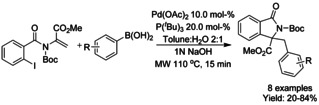
Microwave assisted synthesis of C3‐tetrasubstituted isoindolinones.

In 2018, Mendoza‐Perez and Vazquez described a microwave assisted domino reaction starting from N‐protected iodoaminoacrylates and boronic acids via 5*‐exo–trig* process, followed by Suzuki coupling, to yield isoindolinones bearing quaternary carbon centers (Scheme 40).^[94]^

**Scheme 41:**
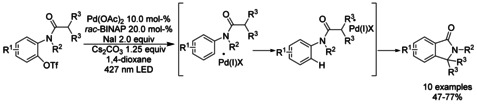
Visible‐light‐induced Pd‐catalyzed C−H arylation of benzamides by Gevorgyan.

Gevorgyan described the synthesis of 3‐substituted isoindolinones involving a visible light induced palladium catalyzed intramolecular C−H arylation of benzamides via fragmentation of C(sp^2^)−O bonds of aryl triflates.^[95]^ The reaction was reported to proceeds via formation of hybrid aryl Pd‐radical intermediates. By using benzamides bearing alkyl, methoxy, halo‐, trifluoromethyl and naphthyl groups, this reaction provides the corresponding isoindolinones in moderate to good yields. Cyclization of cyclohexyl‐ and isobutyl‐derivatized amides was also successful leading to the formation of the products in moderate to good yields (Scheme 41).

**Scheme 42:**
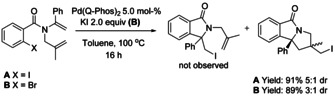
Pd‐catalyzed domino cyclization by Lautens.

In 2011, a Q‐Phos ligated palladium catalyzed domino carbohalogenation reaction forming indoline‐fused isoindolinones containing multiple stereogenic centers was reported by Lautens and co‐workers.^[96]^ Under the reported reaction conditions, the polyunsaturated aryl iodide provides the domino cyclization product in high yield and diastereoselectivity with no formation of monocyclization product. In addition, aryl bromides can be used to form the polycyclic alkyl iodide derivative via halogen exchange domino reaction (Scheme 42).

**Scheme 43:**
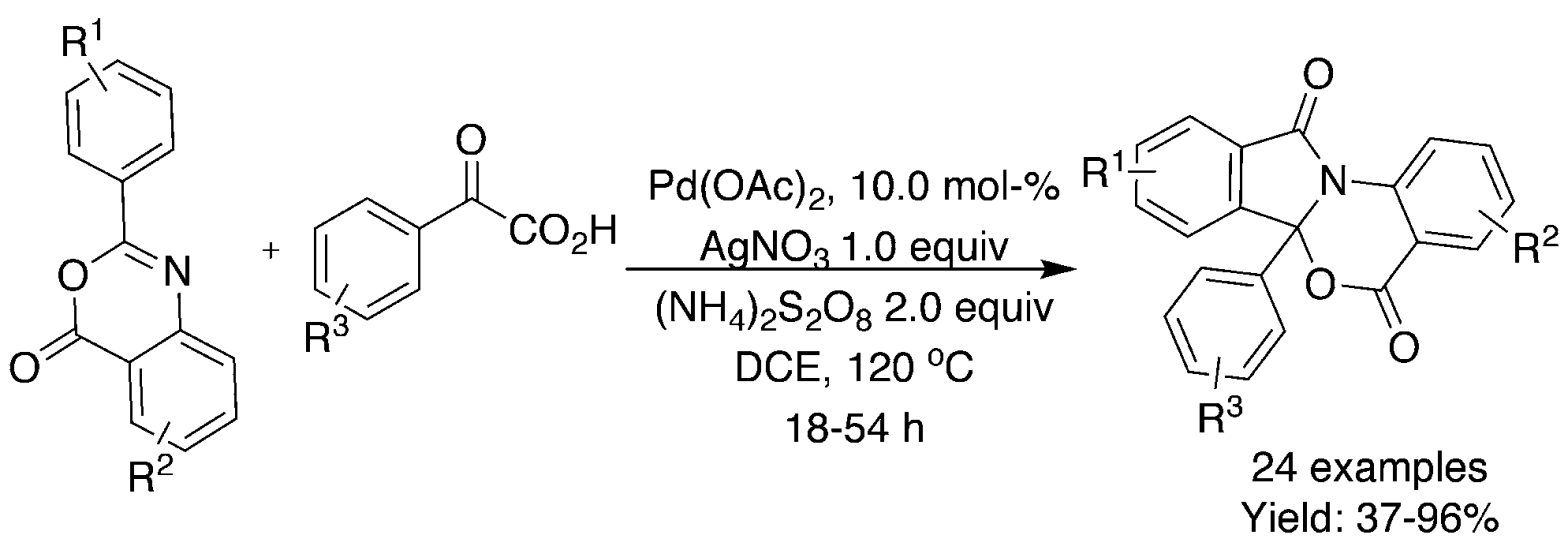
Functionalization of 2‐phenyl‐4*H*‐benzo[*d*][1,3]oxazin‐4‐ones with α‐oxo carboxylic acids.

Singh and co‐workers^[97]^ reported a Pd‐catalyzed decarboxylative free radical approach from various substituted 2‐phenyl‐4*H*‐benzo[*d*][1,3]oxazin‐4‐ones and α‐oxo carboxylic acids in the presence of (NH_4_)S_2_O_8_ as the oxidant and AgNO_3_ as co‐oxidant (Scheme 43). A series of controlled experiments ascertained the formation of an acylated intermediate, which is converted into the targeted product. The reaction was also tested in the presence of the free radical scavenger (2,2,6,6‐tetramethylpiperidin‐1‐yl)oxidanyl (TEMPO) suggesting that the first acylation step follows a radical pathway.

#### Pd‐catalyzed cyclization via dearomatization/Heck reaction

**Scheme 44:**
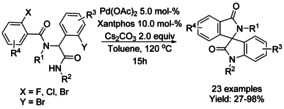
Pd catalyzed cascade cyclization by Van der Eycken.

Vachhani and Van der Eycken reported a strategy to synthesize a broad scope of spiroindolinone‐isoindolinones by Buchwald–Hartwing addition elimination strategy starting from dihalo N‐substituted *N*‐benzamides (Scheme 44).^[98]^ A range of electron‐withdrawing and electron‐donating substituents on the substrates were compatible with these reaction conditions.

**Scheme 45:**
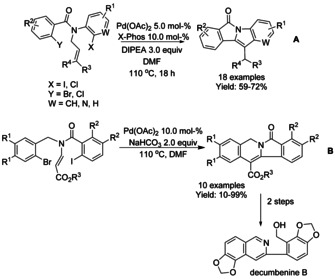
Synthesis of fused isoindolinone rings via Heck reaction.

In 2015, Pal and co‐workers reported a double Heck reaction to yield indolone‐fused isoindolinone rings starting from dihalo *N*‐allyl substituted *N*‐arylbenzamide derivatives.^[99]^ This methodology is based on the reactivity differences of the aromatic halides of the starting amides towards the Pd‐catalyst (Scheme 45 A). The authors proposed that substrates with more reactive halides on the aniline ring (X in Scheme 45 A) than on the aroyl moiety (Y in Scheme 45) is essential for this reaction. One year later, Kim reported a sequential Heck reaction of benzamidoacrylates with Pd(OAc)_2_ and NaHCO_3_ to render an indolizine‐fused isoindolinone ring as part of the strategy for the total synthesis of decumbenine B (Scheme 45 B).^[100]^

**Scheme 46:**
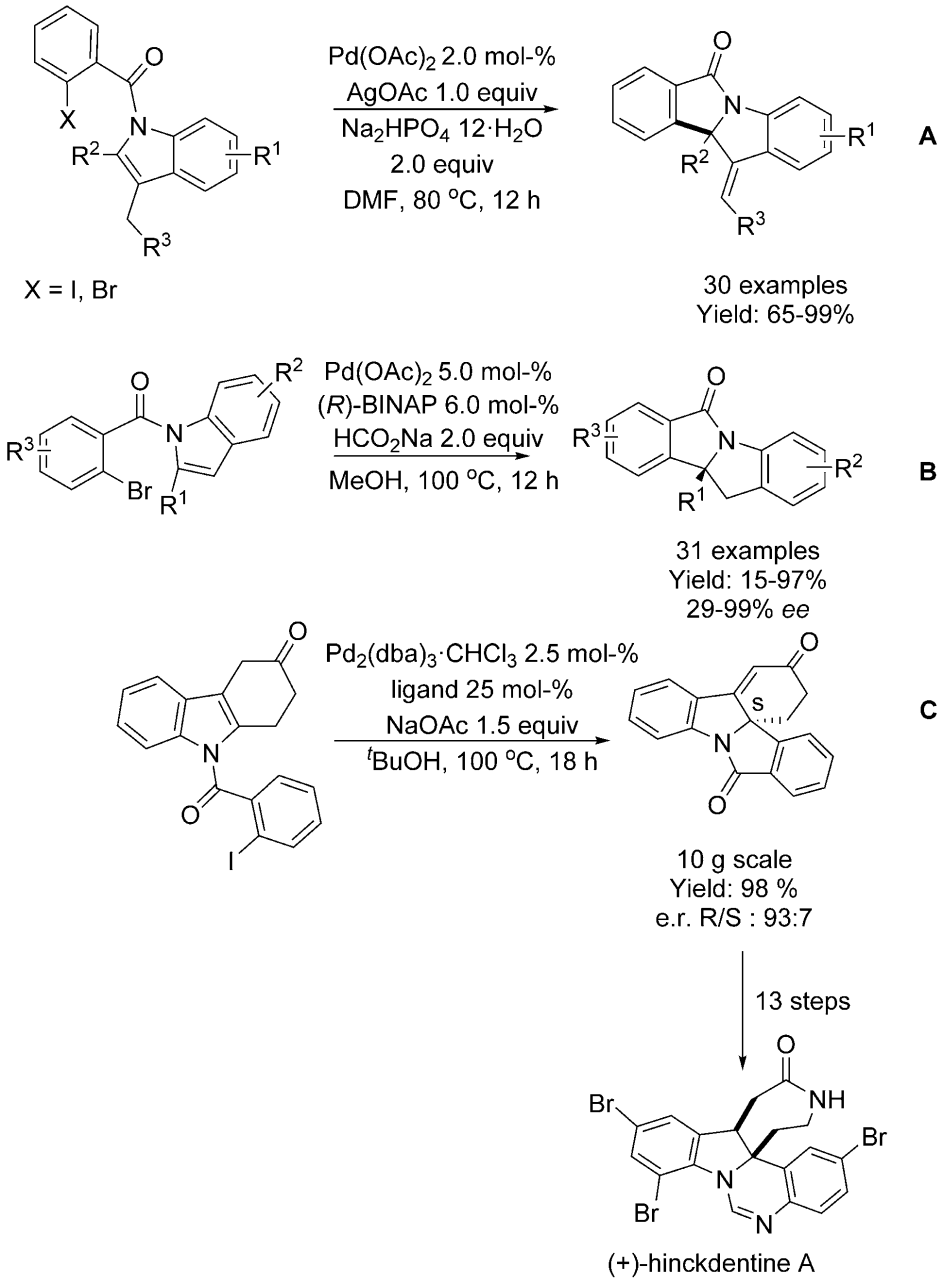
Dearomative Heck type reaction synthesis of polycyclic isoindolinones and intermediate in synthesis of hinckdentine A.

**Scheme 47:**
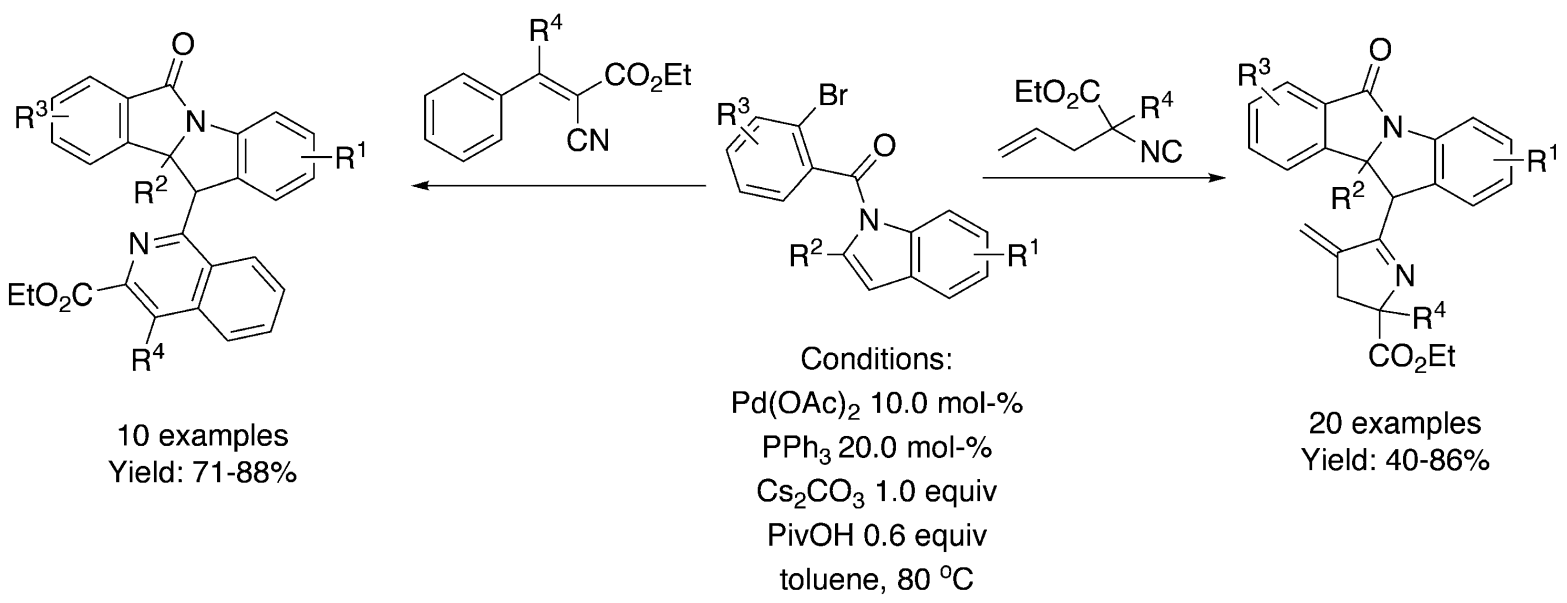
Dearomative aryl/cycloimidoylation of indoles.

Polycyclic isoindolinones can be efficiently synthesized by palladium catalyzed dearomative transformations. Dearomative Heck reactions of indoles have been reported as an efficient method for the construction of indoline‐fused isoindolinone skeleton containing quaternary stereocenters. In 2012, Yao and Wu set a precedence^[101]^ for indole dearomatization methods involving a carbopalladation mechanism, via catalytic intramolecular Heck‐type dearomatization reaction starting from 2,3‐disubstituted indoles (Scheme 46 A). It was shown that steric hindrance at the C2 position of the alkyl substituted indole resulted in lower reaction yields. Jia employed a Pd(OAc)_2_/(*R*)‐BINAP based catalyst system to achieve the first asymmetric indole dearomatization reaction to yield chiral polycyclic isoindolinones with excellent enantioselectivities via reductive Heck reaction using C2‐substituted indoles (Scheme 46 B).^[102]^ Steric effect from aromatic groups at C2‐position in the indole resulted in significantly lower yields. One year later, Kitamura and Fukuyama reported an asymmetric dearomative Heck type cyclization of an *N*‐acyl indole using a palladium catalyst with Feringa's phosphoramide ligand to obtain polycyclic compound bearing the isoindolinone moiety at multigram scale as part of a synthetic strategy for the synthesis of hinckdentine A (Scheme 46 C).^[103]^ Wang, Shang and co‐workers^[104]^ described the first dearomative palladium catalyzed isocyanide insertion reaction for synthesis of indoline‐fused isoindolinones via dearomative aryl/cycloimidoylation of *N‐*(2‐bromobenzoyl)indoles. This methodology was reported to have a wide functional group tolerance (Scheme 47).

**Scheme 48:**
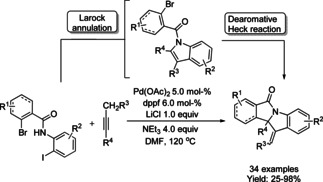
Dearomative arylation of indoles by Jia.

In addition to indole dearomatization transformations, a palladium catalyzed domino Larock annulation/Heck dearomatization reaction to render tetracyclic indoline‐isoindolinones derivatives was investigated.^[105]^ The reaction proceeds via Larock annulation of *N*‐bromobenzoyl *o*‐iodoanilines with alkynes and a subsequent intramolecular dearomative Heck reaction to form three chemical bonds in a single step (Scheme 48). A broad scope of substrates was employed to afford the products in moderate to good yields.

**Scheme 49:**
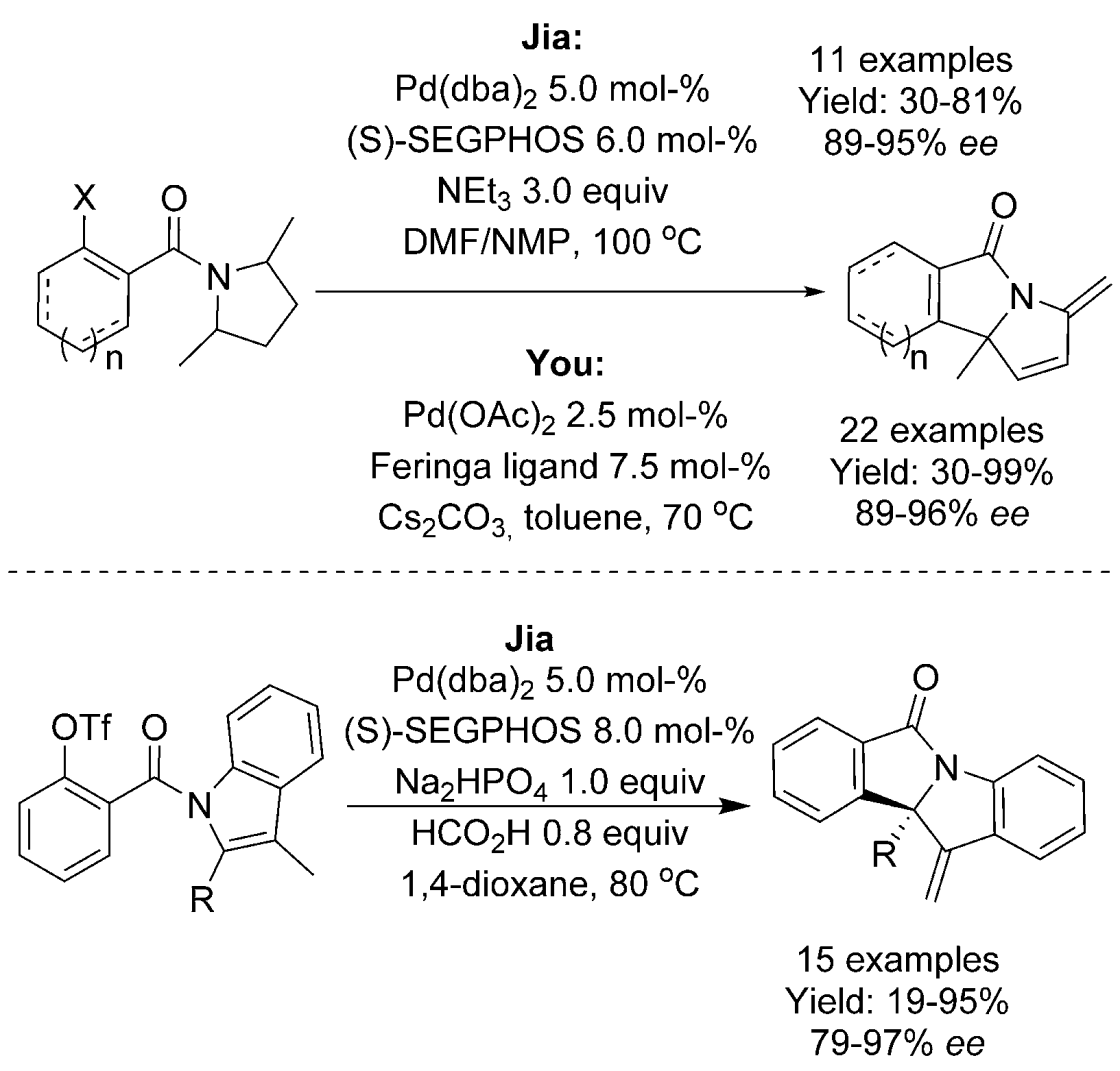
Pyrrole and indole asymmetric dearomatization by You and Jia.

Palladium catalyzed asymmetric intramolecular dearomatizations of pyrroles to yield pyrroline‐fused isoindolinones were described by both Jia^[106]^ and You^[107]^ in 2018 (Scheme 49). Jia reported the use of Pd(dba)_2_ and (*S*)‐SEGPHOS ligand and extended the methodology to the dearomatization of disubstituted indoles using aryl triflate derivatives to obtain the indoline‐isoindolinone products in moderate to high yields and good enantioselectivities. You utilized a Pd(OAc)_2_ as catalysts in collaboration of Feringa's phosphoramide ligand, obtaining similar yields and enantioselectivities as Jia.

**Scheme 50:**
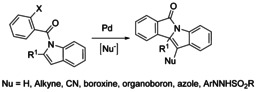
Pd‐catalyzed bisfunctionalization of índoles.

Palladium catalyzed Heck/anion capture sequence reactions represent an important method for 1,2‐difunctionalization of alkenes, which starts with an alkene carbopalladation with subsequent coupling of alkyl‐Pd with nucleophiles. A series of effective examples using this type of domino reactions as a good strategy for the dearomatization of indoles to render bifunctionalized indoline‐fused isoindolinones bearing quaternary carbon centers have been reported. Such methodologies typically involve the use of a range of external nucleophiles including H‐phosphonates,^[108]^ hydrides,^[109]^ alkynes,^[110]^ cyanides,^[111]^ boroxines,^[112]^ organoboron esters,^[113]^ azoles^[114]^ and *N*‐arylsulfonylhydrazones^[115]^ as capturing agents to trap the benzyl‐Pd species derived from the carbopalladation step (Scheme 50).

#### Pd‐catalyzed isocyanide insertion‐cyclization

**Scheme 51:**
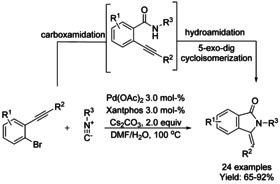
Pd‐catalyzed triple tandem reaction towards isoindolinones by Khan and Pardasani.

Khan and Pardasani reported a one‐pot Pd‐catalyzed isocyanide insertion, imine hydration and 5‐*exo*–*dig* cycloisomerization sequence reaction by using an isocyanide as amide surrogate. In this reaction, an alkylbenzamide is generated in situ followed by cyclization to give the isoindolinone derivative (Scheme 51).^[116]^ The nature of the substituent on the triple bond had a major impact on the reaction. Aromatic groups containing an electron‐withdrawing substituent increased the efficiency of the reaction to produce the isoindolinone by decreasing the electron density at the proximal end of the triple bond. Alkyl and alkenyl substituents failed to produce the cyclized product and, instead, furnished an amide intermediate as the only product. The authors proposed that, in this reaction, the electrophilicity of the alkyne is the driving force of the tandem process and the presence of the Pd catalyst is crucial only in the carboxamidation step.

**Scheme 52:**
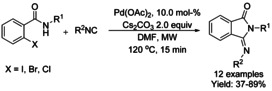
Pd‐catalyzed coupling reaction by Chauhan.

In 2013, Chauhan described a microwave assisted highly stereoselective ligand free reaction protocol using amides and isocyanides as coupling partners to render substituted isoindolinones with high stereoselectivity via isocyanide insertion (Scheme 52). In this reaction a large variety of amides are well tolerated, except for 2‐chloro or 2‐bromo‐substituted amide substrates resulting in lower yields.^[117]^

### Nickel‐catalyzed coupling reactions

#### Ni‐catalyzed cyclization via alkylation

**Scheme 53:**
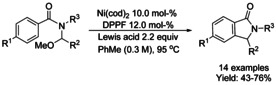
Ni‐catalyzed intramolecular cyclization.

In 2011 Shacklady‐McAtee^[118]^ and co‐workers described an intramolecular cyclization of *N*‐benzoyl aminals mediated by Ni^0^ in the presence of a Lewis acid to render a variety of isoindolinones C3‐substituted in moderate to good yields (Scheme 53). The reaction proceeds in three steps from benzoyl chloride, primary amines and aldehydes via initial formation of iminium ion intermediate with the help of the Lewis acid followed by an α‐amidoalkyl nickel (II) intermediate. An electron‐rich Ni^0^ catalyst is required for the formation of this in intermediate, which is then followed by the cyclization to final product.

#### Ni‐catalyzed cyclization via dearomatization/Heck reaction

**Scheme 54:**
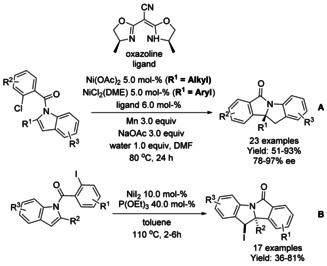
Nickel catalyzed dearomative synthesis of isoindolinones.

In 2017, a Ni‐catalyzed asymmetric Heck type dearomatization reaction starting from C2‐substituted indoles to afford fused isoindolinone‐indolines with high enantioselectivity was reported by Zhou (Scheme 54 A).^[119]^ The authors proposed that this mechanism is distinct from the analogous palladium catalyzed methods.[^101^, ^102^] In this process, a nickel−carbon bond is converted into a C−H bond to release the product via protonation. This investigation constitutes the first example of a nickel‐catalyzed asymmetric reductive Heck cyclization. Recently, Lautens^[120]^ reported a dearomative carboiodination reaction to install reactive secondary benzylic iodides (Scheme 54 B). The authors proposed that this reaction proceeds by the means of a *syn* intramolecular carbonickelation across a 2‐substituted indole with subsequent diastereoretentive reductive elimination of the carbon‐iodine bond to afford fused isoindolinone‐indoline rings. A wide variety of functional groups were well tolerated affording the products in moderate to good yields with excellent diastereoselectivities.

### Copper‐catalyzed coupling reactions

#### Cu‐catalyzed cyclization via olefination/alkylation

**Scheme 55:**
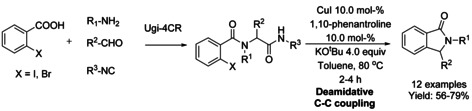
Ugi‐4CR reaction followed by Cu‐catalyzed deamidative C−C coupling by Chahuan.

The use of copper catalyzed coupling reactions for synthesis of isoindolinones from products obtained from Ugi‐4CR reaction was described by Chauhan.^[121]^ The diamides obtained from the Ugi‐4CR reaction were cyclized to isoindolinones via Cu‐catalyzed deamidative C−C coupling. This two‐step sequence allows the synthesis of isoindolinone derivatives using a broad range of 2‐halobenzoic acids, isocyanides, amines and aldehydes (Scheme 55).

**Scheme 56:**
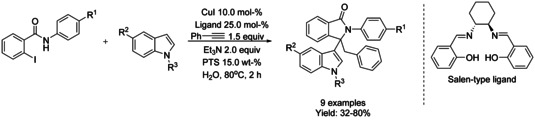
Synthesis of isoindolinones in an aqueous micellar medium by Sen.

Sen^[122]^ reported a one‐pot palladium‐free copper‐mediated domino Sonogashira‐5‐*exo*–*dig*‐cyclization of *o*‐iodo‐*N*‐phenylbenzamides with phenylacetylene followed by regioselective nucleophilic addition of indole using CuI as the catalyst and a salen‐type ligand to obtain a library of indolyl isoindolinones in high yield and in short reaction time in aqueous micellar medium (Scheme 56). Using *o*‐bromo‐*N*‐phenylbenzamides, a lower reaction yield was observed and using the corresponding aryl chlorides the reaction was found to be inefficient.

**Scheme 57:**
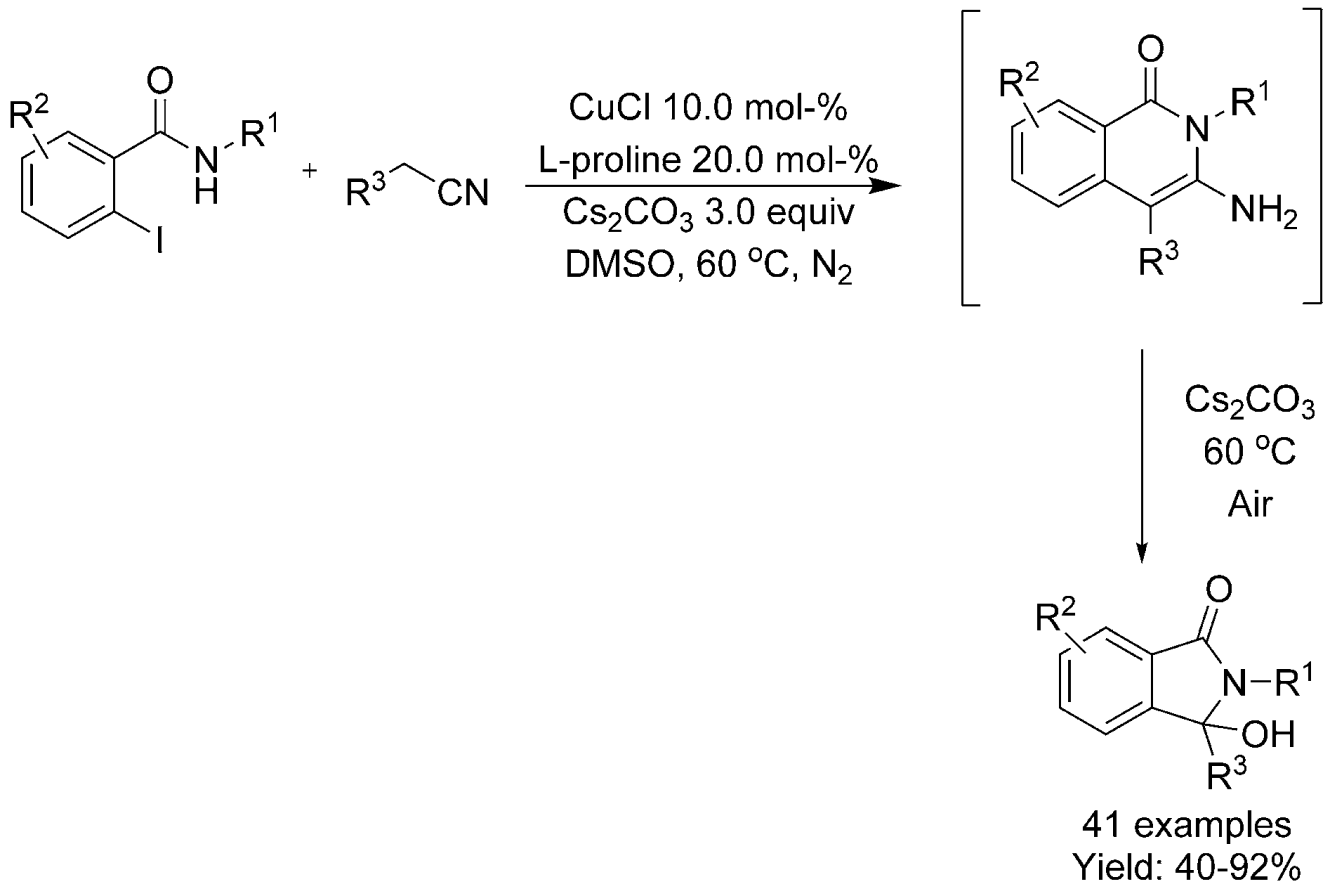
Synthesis of 3‐hydroxyisoindolinones by Yao.

In 2020, Yao reported a one‐pot two‐step sequential carbon degradation/ring contraction reaction for the synthesis of 3‐hydroxyisoindolinones from iodo‐benzamides and various substituted benzyl cyanides as benzoyl synthons in the presence of CuCl and l‐proline as the ligand under nitrogen atmosphere (Scheme 57).^[123]^ The authors described the initial formation of the corresponding substituted aminoisoquinolinone, which showed to be stable after 12 h. Without isolating the isoquinoline derivative, the reaction vessel was opened to air allowing the ring contraction to take place. A broad reaction scope of various 3‐hydroxyisoindolinones was presented in moderate to good yields. The reactions performed using 2‐bromobenzyl cyanide substrates did not require the use of l‐proline as a ligand.

**Scheme 58:**
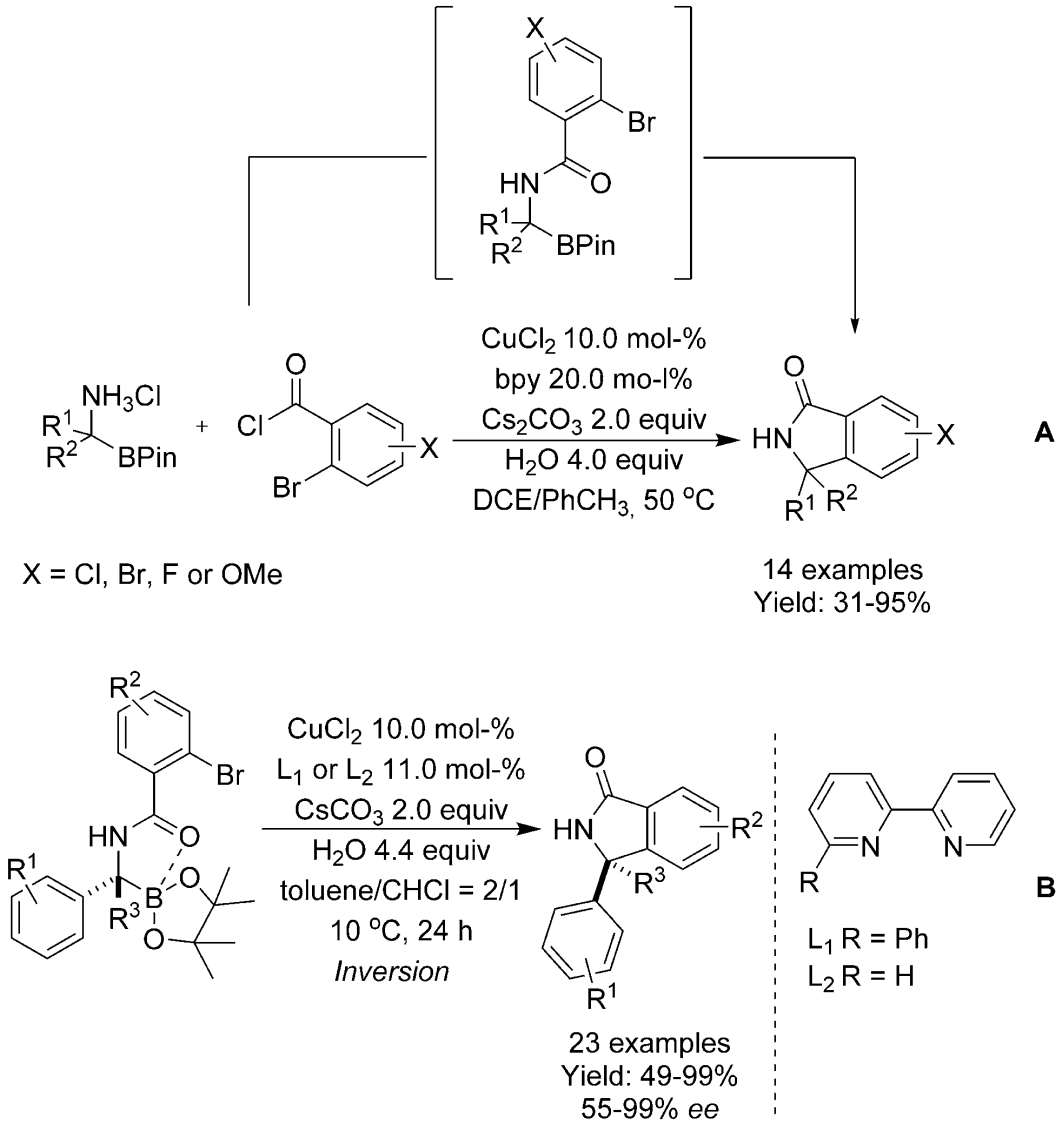
Copper catalyzed cross‐coupling alkylborons for synthesis of isoindolinones.

In 2016, a domino amidation/arylation reaction of quaternary and tertiary aminoboronates with 2‐halobenzoyl chlorides in the presence of 2,2'‐bipyrydine/copper(II) catalyst was reported by Dumas and co‐workers (Scheme 58 A).^[124]^ A variety of acid chlorides were examined by the authors, aryl chlorides at any position of the ring were well tolerated (X in Scheme 58 A), as well as fluoride and electron‐donating methoxide were found compatible with the reaction conditions. Also, aryl bromides underwent cyclization exclusively at the 2‐Br group without the formation of intermolecular coupling products. The reaction performed well starting from aminoboronates containing an adjacent aromatic ring and bearing halogen and electron‐donating groups. This reaction is the first reported example of arylation of fully substituted aminoboronates or Cu‐catalyzed couplings of quaternary boronates. The authors proposed that the reaction proceed via configurationally unstable Cu^I^‐intermediate as a racemic isoindolinone was observed when enantioenriched aminoboronates were utilized as starting materials. Based on the amidation/arylation sequence reported by Dumas, a stereospecific intramolecular Suzuki–Miyaura type cross‐coupling reaction of easily accessible enantioenriched α‐[(*o*‐bromobenzoyl)amino]benzylboronates using bipyridine‐copper catalyst was described by Yamamoto and Suginome (Scheme 58 B).^[125]^ This reaction proceeds with stereochemical inversion relying on the stereoretentive transmetalation step, which is favored by using a Brønsted acid additive. The highest enantiospecificities were achieved using substrates with electron‐donating substituents in the *para*‐position of the pendant benzyl moiety (R^1^ at Scheme 58 B). Notably, sterically hindered groups at the stereogenic carbon afforded high enantiospecificities. In case of fully substituted stereogenic boron‐bound carbon atom centers (R^3^ = Me at Scheme 58 B), less sterically demanding bipyridine ligands resulted in higher enantioselectivities.

**Scheme 59:**
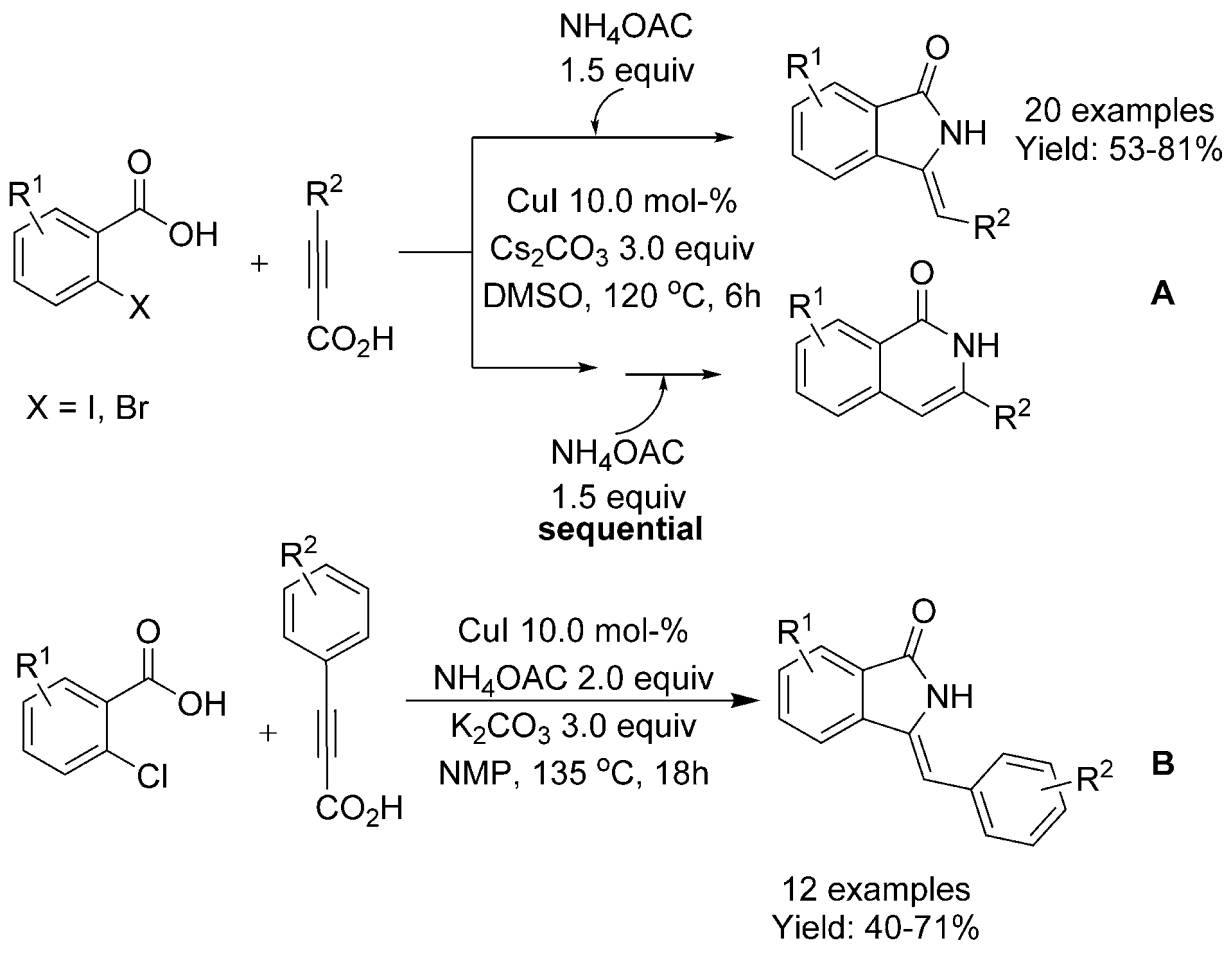
Synthesis of isoindolinone derivatives via decarboxylative coupling using iodo‐ or bromobenzoic acids by Lee.

While the majority of the reported procedures for the preparation of 3‐methyleneisoindolinones describe the use of 2‐halobenzamides, a Cu‐catalyzed one‐pot synthesis of targeted compounds via decarboxylative coupling of arylakynylcarboxylic acid with iodo‐ or bromobenzoic acid was reported by Lee in 2014.^[126]^ This ligand free structurally divergent reaction methodology affords isoindolinone or isoquinolinone derivatives selectively from the same substrates by switching from one‐pot reaction to sequential addition ammonium acetate (NH_4_OAc) (Scheme 59 A). A broad range of arylalkynylcarboxylic acid were well tolerated, however, in the presence of aliphatic analogues no isoindolinone derivatives were observed. The methodology was further extended to the chloro‐benzoic acid substrates by the same authors (Scheme 59 B).^[127]^

**Scheme 60:**
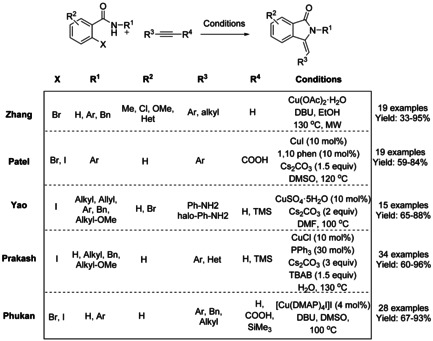
Synthesis of methyleneisoindolinone derivatized from 2‐halobenzamides.

A series of Cu‐catalyzed domino synthetic protocols for the synthesis of 3‐methyleneisoindolinones starting from 2‐halobenzamides and alkynes have been reported (Scheme 60).[^128^, ^129^, ^130^, ^131^, ^132^] In 2013, Zhang and co‐workers reported a stereoselective microwave assisted synthesis of (*Z*)‐3‐methyleneisoindolinone derivatives from N‐substituted‐2‐bromobenzamides and arylacetylenes using a Cu(OAc)_2_⋅H_2_O/DBU catalyst.^[128]^ Later, a Cu‐catalyzed decarboxylative cross‐coupling of N‐substituted‐2‐halobenzamides with aryl alkynyl carboxylic acids, followed by 5‐*exo*–*dig* heteroannulation, was reported by the Patel.^[129]^ The use of acid derivatives as alkyne surrogates has advantages, such as higher reactivity or lower susceptibility to homocoupling reaction. For 2‐bromo benzamides, a CuI/1,10‐phenantroline catalyst system is needed, however, when more reactive 2‐iodo benzamides were used as substrates the reaction proceeded smoothly in the absence of ligand without affecting the yield. In 2016, Yao^[130]^ reported the reaction of alkynylanilines with 2‐iodo‐*N*‐methylbenzamides in the presence of a copper catalyst to form 3‐(2‐aminobenzylidene)‐2‐substituted isoindolinone derivatives via C‐terminal attack of the 2‐alkynylaniline to 2‐iodobenzamide. The isoindolinone products were converted to spiro fused isoindolinone‐indolines utilizing halonium ion mediated strategy using NBS/TCC in the presence of acetic anhydride. In the same year, the Prakash^[131]^ described a triple domino desilylation, cross‐coupling and hydroamidation sequence under aqueous phase‐transfer conditions. Use of (silyl)alkynes as coupling partners afforded the products with exclusive *Z*‐configuration, with the exception of (silyl)alkynes containing strong electron‐withdrawing substituents. The scope of the (silyl)alkyne derivatives was found to be broad and allowed access to the target molecules with a varied range of functionalities in good yields. N‐Unprotected iodobenzamides were well tolerated, affording the substituted isoindolinones in high yield. In 2018, Phukan described a domino reaction of unprotected 2‐halobenzamide and terminal alkynes using a square pyramidal [Cu(DMAP)_4_I]I complex as a catalyst to yield the corresponding (*Z*)‐3‐methyleneisoindolinones with free NH group via cross‐coupling reaction followed by 5‐*exo*–*dig* cycloisomerization.^[132]^ This methodology allows the use of a broad range of terminal alkynes. The authors also described the extension of this methodology to phenyl propiolic acid derivatives and silylated alkynes with N‐protected‐bromobenzamides to afford the products in good yield.

### Palladium and copper catalyzed coupling reactions

#### Pd/Cu‐catalyzed cyclization via olefination

**Scheme 61:**
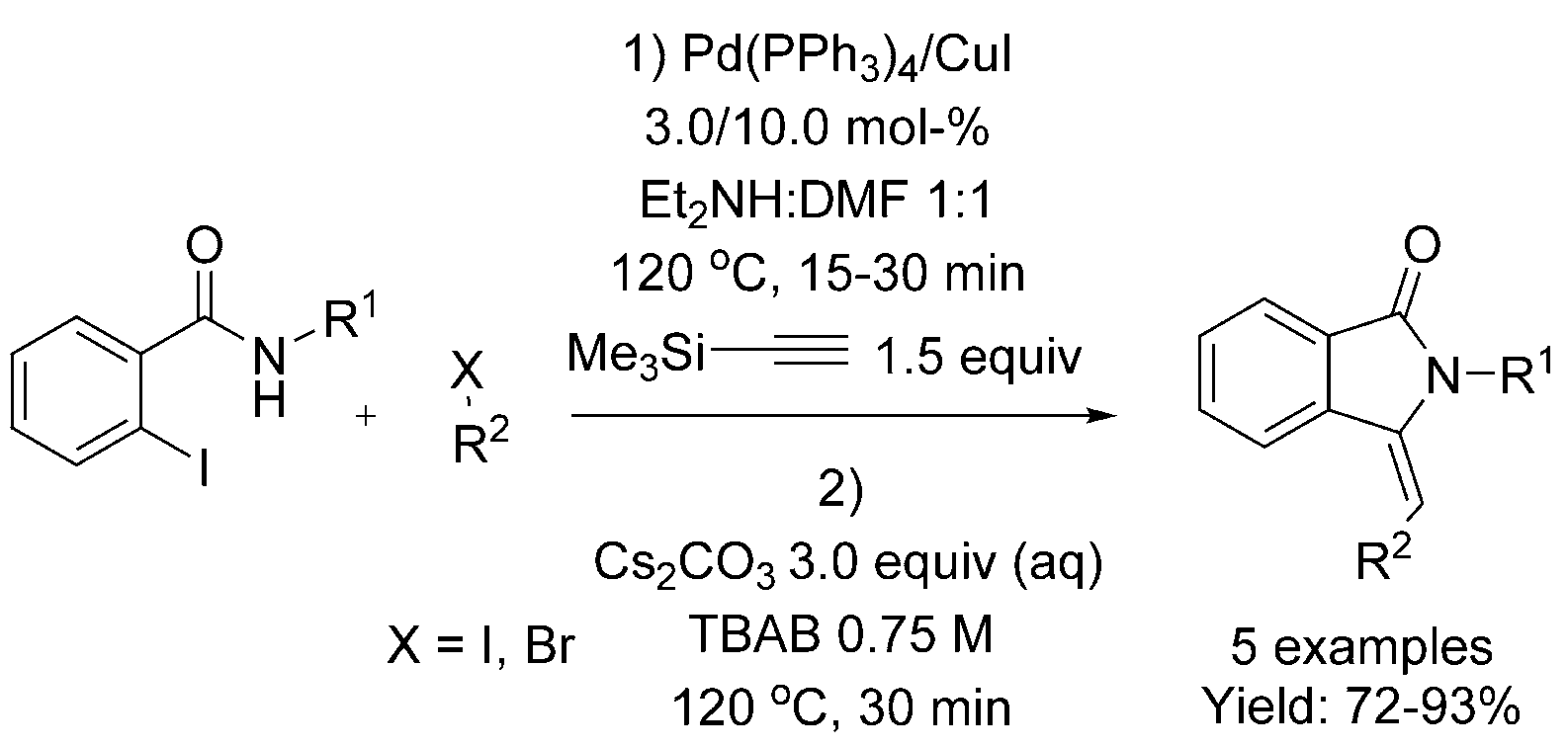
Pd/Cu‐catalyzed synthesis of isoindolinones.

Based on the advantages of utilizing alkynylsilanes as coupling partners to afford 3‐methylene‐isoindolinones reported by Prakash in 2016, a multicomponent one‐pot reaction using TMSA, 2‐iodo‐*N*‐methylbenzamide and aryl halides (bromides or iodides) under Pd/Cu catalysis was investigated and reported by the same author.^[131]^ This protocol affords the corresponding isoindolinones in high yields (Scheme 61) and emphasizes the importance of using alkylsilanes to facilitate consecutive cross‐coupling processes in one‐pot procedures from aryl electrophiles and TMSA as acetylene surrogate.

**Scheme 62:**
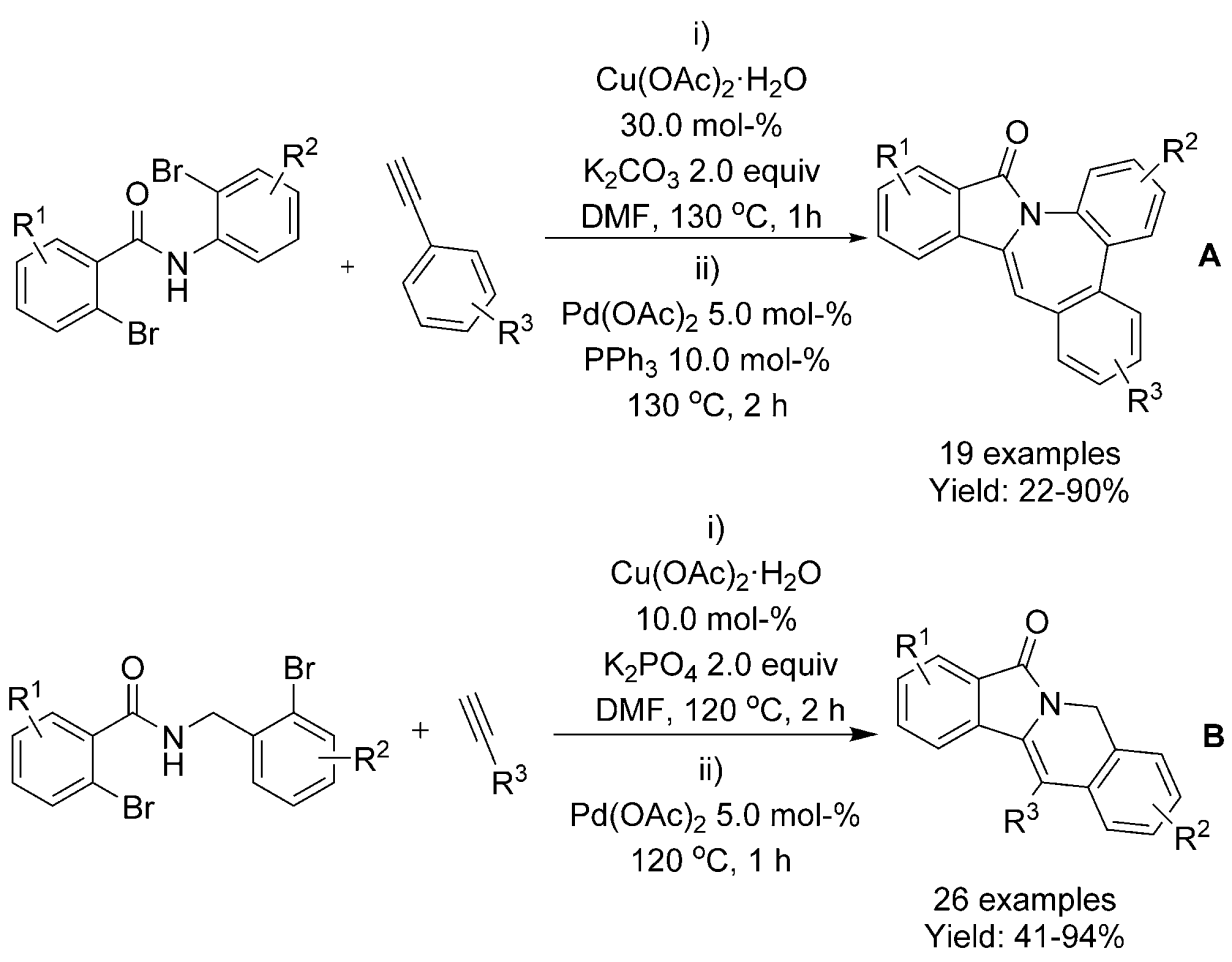
Synthesis of azepino‐fused isoindolinones and isoindolo[2,1‐*b*]isoquinolin‐7(5*H*)‐one reported by Kumar.

Kumar^[133]^ developed a one‐pot sequential strategy for the synthesis of azepino‐fused isoindolinones. A copper catalyzed Sonogashira coupling and intramolecular hydroamination was utilized to afford isoindolinones, followed by a Pd‐catalyzed intramolecular direct arylation reaction for the transformation of the isoindolinones formed to the targeted polyheterocycles (Scheme 62 A). This reaction starts from 2‐bromo‐*N‐*(2‐bromophenyl)benzamide and tolerates a broad range of phenylacetylene derivatives. Later, the synthesis of isoindolo[2,1‐*b*]isoquinolin‐7(5*H*)‐one from 2‐bromo‐*N‐*(2‐bromobenzyl)benzamides using a similar sequential strategy was described by the Kumar^[134]^ with broad substrate scope and functional group compatibility (Scheme 62 B).

### Cobalt‐catalyzed coupling reactions

#### Co‐catalyzed alkylation

**Scheme 63:**
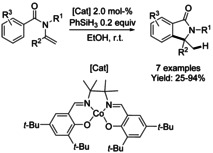
Co‐hydroarylative cyclization by Arai.

Co‐catalyzed hydroarylative cyclization of *N*‐(methylethenyl)‐*N*‐benzyl benzamides and PhSiH_3_ to afford C3‐tetrasubstituted *N*‐benzyl isoindolinones was reported by the Arai^[135]^ (Scheme 63). This method enables facile control of regiochemistry and discrimination of two inequivalent aromatic rings, tolerating a variety of substrates.

## Carbonylation: Annulation/Cyclization

Transition metal‐catalyzed carbonylation reactions, are widely used in synthetic chemistry. The simplicity and low cost of carbon monoxide (CO) as C‐1 unit as well as its application in green chemistry and wide use in industry settings are among the most significant advantages of carbonylation reactions. A number of reviews^[136]^ on the use of this type of transformations in heterocycle synthesis have appeared. Here, we intend to report a brief update on transition metal catalyzed carbonylation reactions applied into isoindolinone synthesis.

### Palladium catalyzed carbonylation reactions

#### Pd‐catalyzed carbonylation via C−H functionalization

**Scheme 64:**
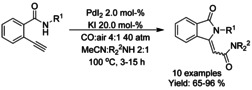
Aminocarbonylation‐heterocyclization of 2‐ethynylbenzamides.

3‐Alkylidene isonindolinones were prepared by Garbiele and Mancuso by aminocarbonylation‐heterocyclization of 2‐ethynylbenzamides (Scheme 64).^[137]^ A combination of palladium(II) iodide and potassium iodide was utilized as a catalyst to facilitate the oxidative monoaminocarbonylation of alkynes with nucleophilic secondary amines, followed by heterocyclization to 3‐alkylidene isoindolinone. The diastereoselectivity of the formed alkenes was mainly towards *Z*‐isomer but depending on the steric constraints formed by substrates a full diastereoselectivity towards *E*‐stereoisomer was also observed.

**Scheme 65:**
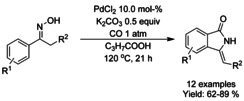
Carbonylative synthesis of 3‐alkylidene isoindolinones from ketimines and oximes.

Jiang^[138]^ reported a palladium‐catalyzed carbonylation of aromatic oximes via *ortho*‐C(sp^2^)−H bond functionalization (Scheme 65). While the reaction does take place under atmospheric carbon monoxide pressure, a significant amount of palladium catalyst is required. Nevertheless, in this manner a variety of *N*‐underivatized 3‐alkylidene isoindolinones can be generated in good yields.

**Scheme 66:**
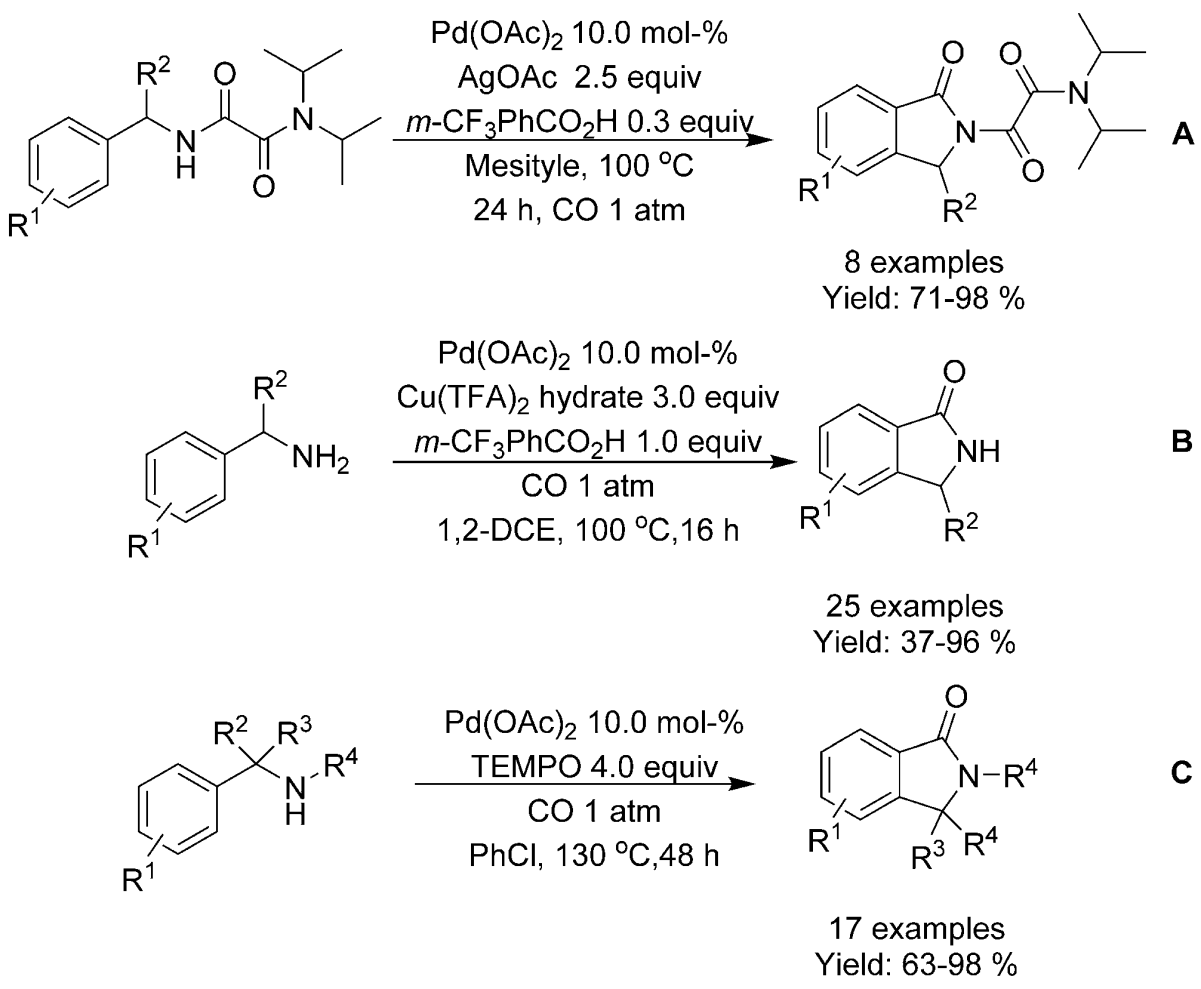
Palladium‐catalyzed carbonylation of benzyl amines via C−H functionalization.

Wang et al.^[139]^ presented an oxalyl amide assisted palladium‐catalyzed carbonylation via C−H functionalization for synthesis of pyrrolidones which was applied to a small scope of isoindolinones (Scheme 66 A). The combination of silver salt as an oxidant and *meta*‐(trifuoromethyl)benzoic acid (*m*‐CF_3_PhCO_2_H) as an additive was found to be vital to achieve high yields. Later, Zhang et al.^[140]^ applied a very similar carbonylation approach to unprotected benzyl amines utilizing copper(II) trifuoroacetate as the oxidant instead of silver (Scheme 66 B). As with many *ortho*‐C−H functionalization methods, substrates with *ortho*‐substituents resulted in significantly lower yields. Carbonylation of sterically hindered *N*‐alkyl or *N*‐aryl benzylamines using TEMPO as a stoichiometric oxidant was reported by Cheng et al. (Scheme 66 C).^[141]^ The method provided good to high yields for *N*‐methyl/ethyl derivatized substrates and lower yields for *N*‐aryl derivatized. Finally, the methodology was successfully applied to the synthesis of spiropachysin‐20‐one.

**Scheme 67:**
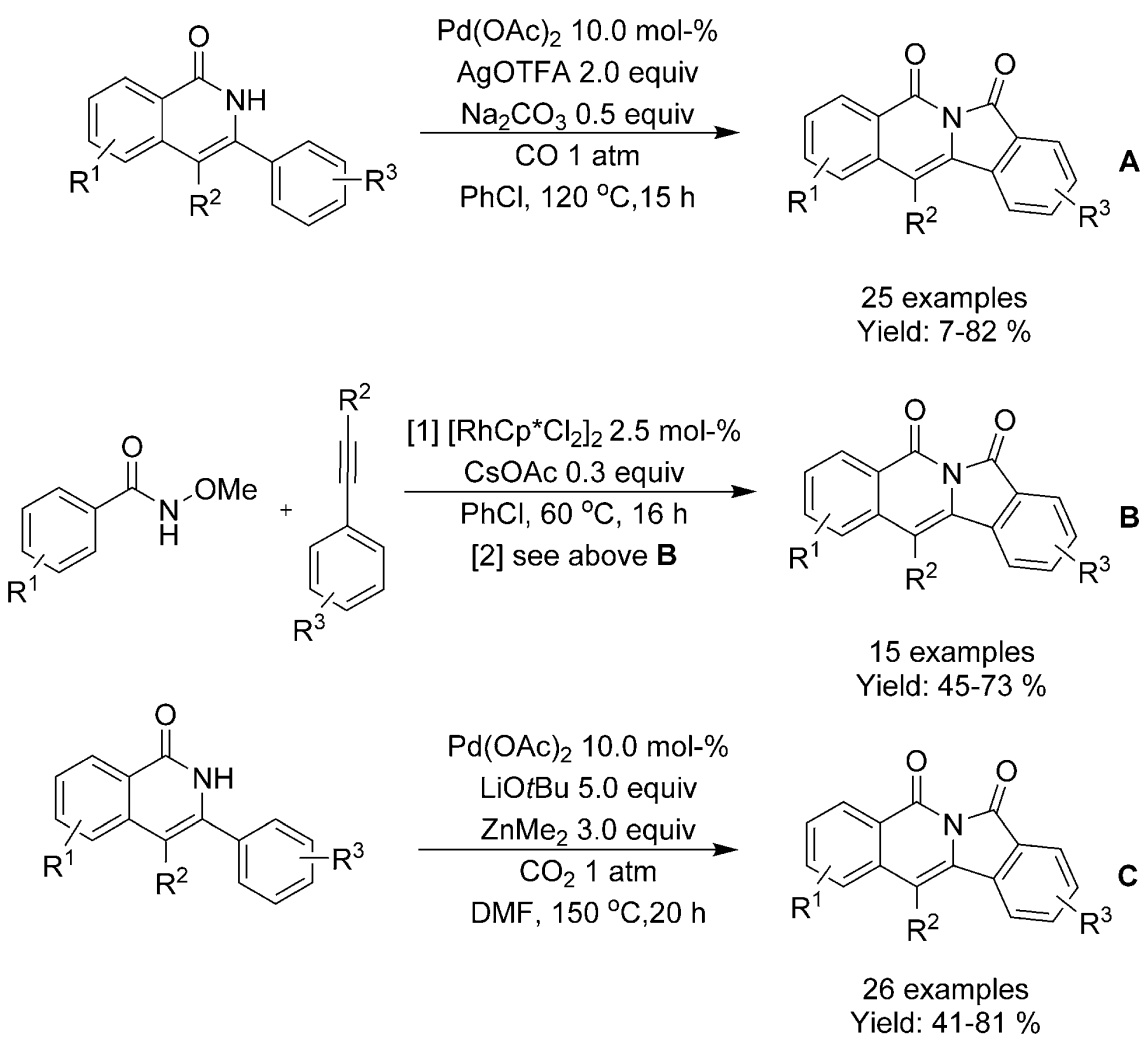
Synthesis fused polycyclic isoindolinone derivatives via palladium catalyzed carbonylation.

Palladium catalyzed cyclocarbonylation (Scheme 67 A) and rhodium/palladium relay catalysis (Scheme 67 B) were applied to the synthesis of isoindolo[2,1‐*b* ]isoquinoline‐5,7‐diones by Guo et al.^[142]^ In the case of the cyclocarbonylation, a wide range of substrates were tolerated with only drastic drop in the yield observed with *ortho*‐substituents (R^3^) in the pendant arene group. The arenes utilized in the cyclocarbonylation are commonly prepared via rhodium catalyzed annulation of suitable benzamide and aryl alkyne, and thus combined into one‐pot two‐step synthesis to achieve similar structures in facile manner. Instead of carbon monoxide, Wang^[143]^ utilized carbon dioxide in the synthesis of isoindolo[2,1‐*b*]isoquinoline‐5,7‐diones via cyclocarbonylation in moderate to good yields (Scheme 67 C). Similarly as with method described by Guo the *ortho*‐substituent (R^3^) in the pendant arene group did not allow the carbonylation to take place. It was proposed that the activation of carbon dioxide proceeds via formation of hemicarbonate ion with lithium *tert*‐butoxide followed by ligand exchange with palladium(II) acetate forming the starting active catalytic complex.

#### Pd‐catalyzed carbonylation via C−X functionalization

**Scheme 68:**
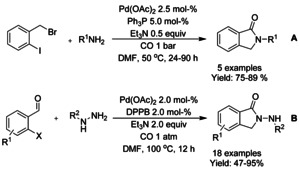
Carbonalytive multi‐component reactions for synthesis of isoindolinones.

A multi‐component palladium catalyzed carbonylation reaction was presented by Kollár^[144]^ and Han et al.^[145]^ Kollar used 2‐iodobenzyl bromide in the presence of primary amines under carbon monoxide atmosphere to successfully synthesize a small scope of *N*‐derivatized isoindolinones (Scheme 68 A). Han et al. described an in situ condensation of 2‐bromobenzaldehydes and phenylhydrazines followed by palladium catalyzed carbonylation to generated 2‐amino isoindolinones (Scheme 68 B). Here, both 2‐bromo and 2‐iodobenzladehydes resulted in good to excellent yields but 2‐triflate bearing analogs yielded in significantly inferior outcome. Also, strongly electron withdrawing substituents on the benzene ring of the hydrazine reactants resulted in lower yields.

**Scheme 69:**
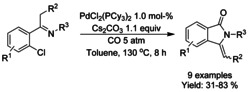
Carbonylative synthesis of 3‐alkylidene isoindolinones from ketimines and oximes.

Cyclocarbonylative coupling of *ortho*‐chloro arylketimines with carbon monoxide was described by Hua^[146]^ using phosphine ligated palladium catalyst at elevated temperatures (Scheme 69). In this manner, a number of 3‐methylidene isoindolinones were produced in good yields but switching to synthesis of 3‐ethylidene analog has proved challenging for the methodology.

**Scheme 70:**
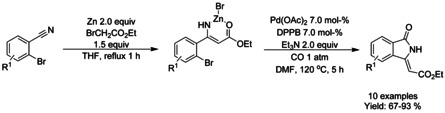
Synthesis of (*Z*)‐3‐methyleneisoindolinones via tandem sequential Blaise reaction carbonylation.

Tandem stereocontrolled one‐pot synthesis of (*Z*)‐3‐methyleneisoindolinones via tandem sequential Blaise reaction/aminocarbonylation was described by Lee.^[147]^ In situ generated Reformatsky reagent was reacted with 2‐bromobenzonitriles followed by palladium catalyzed carbonylation with carbon monoxide at atmospheric pressure (Scheme 70). In general, electron donating substituents (R^1^) in 2‐bromobenzonitriles provided very good yields of isoindolinone derivatives, whereas electron withdrawing halides resulted into lower isolated yields. In addition, a route to derivatized aristolactams from these (*Z*)‐3‐methyleneisoindolinone derivatives was described.

**Scheme 71:**
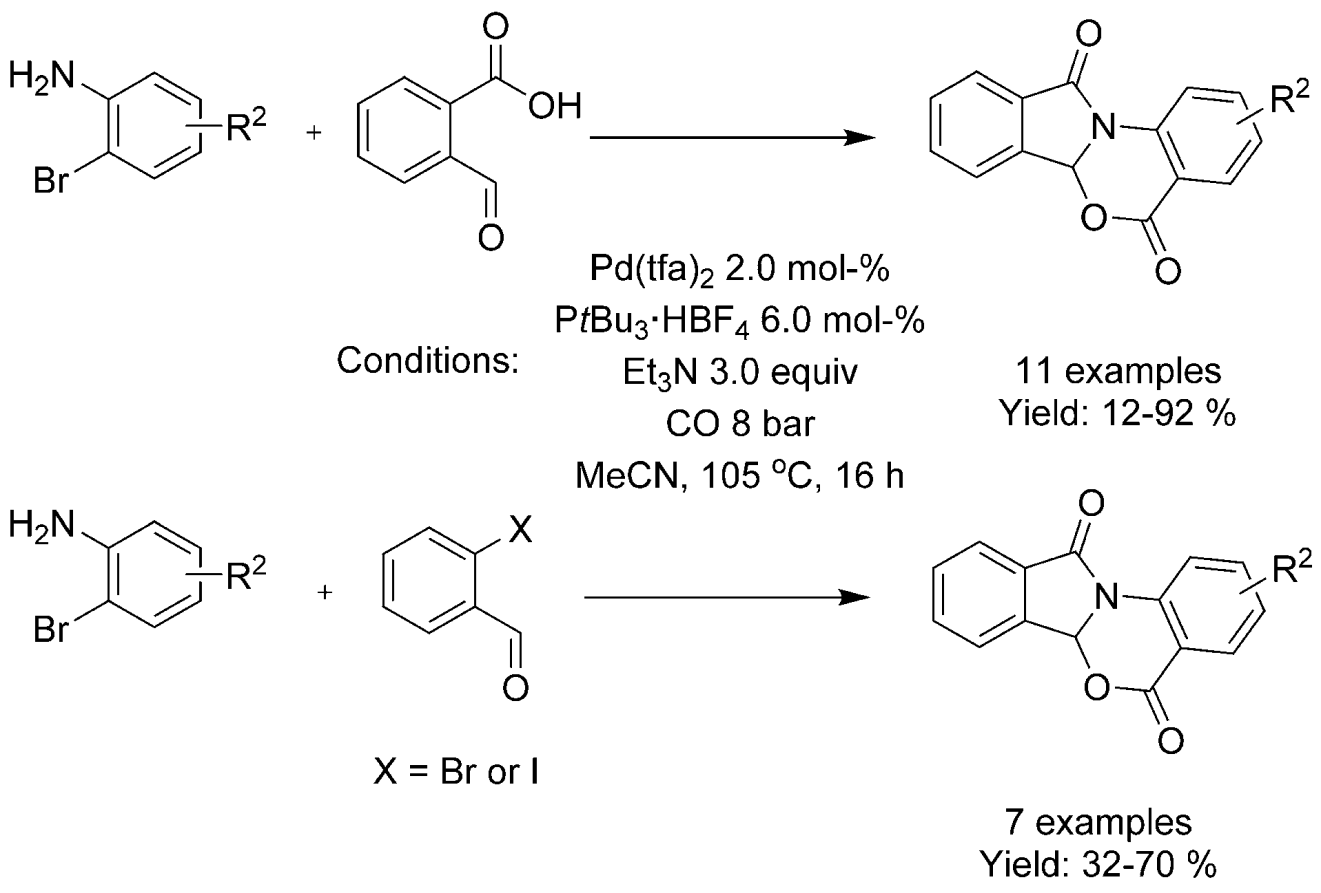
Synthesis of functionalized isoindolinones from 2‐bromoanilines with 2‐formylbenzoic acid or 2‐halobenzaldehydes.

A multi‐component cascade reaction utilizing either carbonylation or double‐carbonylation process of 2‐bromoanilines with 2‐formylbenzoic acid or 2‐halobenzaldehydes generating functionalized isoindolinones was reported by Wu (Scheme 71).^[148]^ In the case of 2‐formylbenzoic acids, the use of 3‐amino‐2‐chloropyridine instead of 2‐bromoanilines resulted in significant drop in the yield but otherwise halide, methyl and acetyl substituents were well tolerated. In the case of 2‐halobenzaldehydes, 2‐iodobenzaldehyde substrates resulted in the highest yields, but still lower than what was obtained with 2‐formylbenzoic acids.

**Scheme 72:**
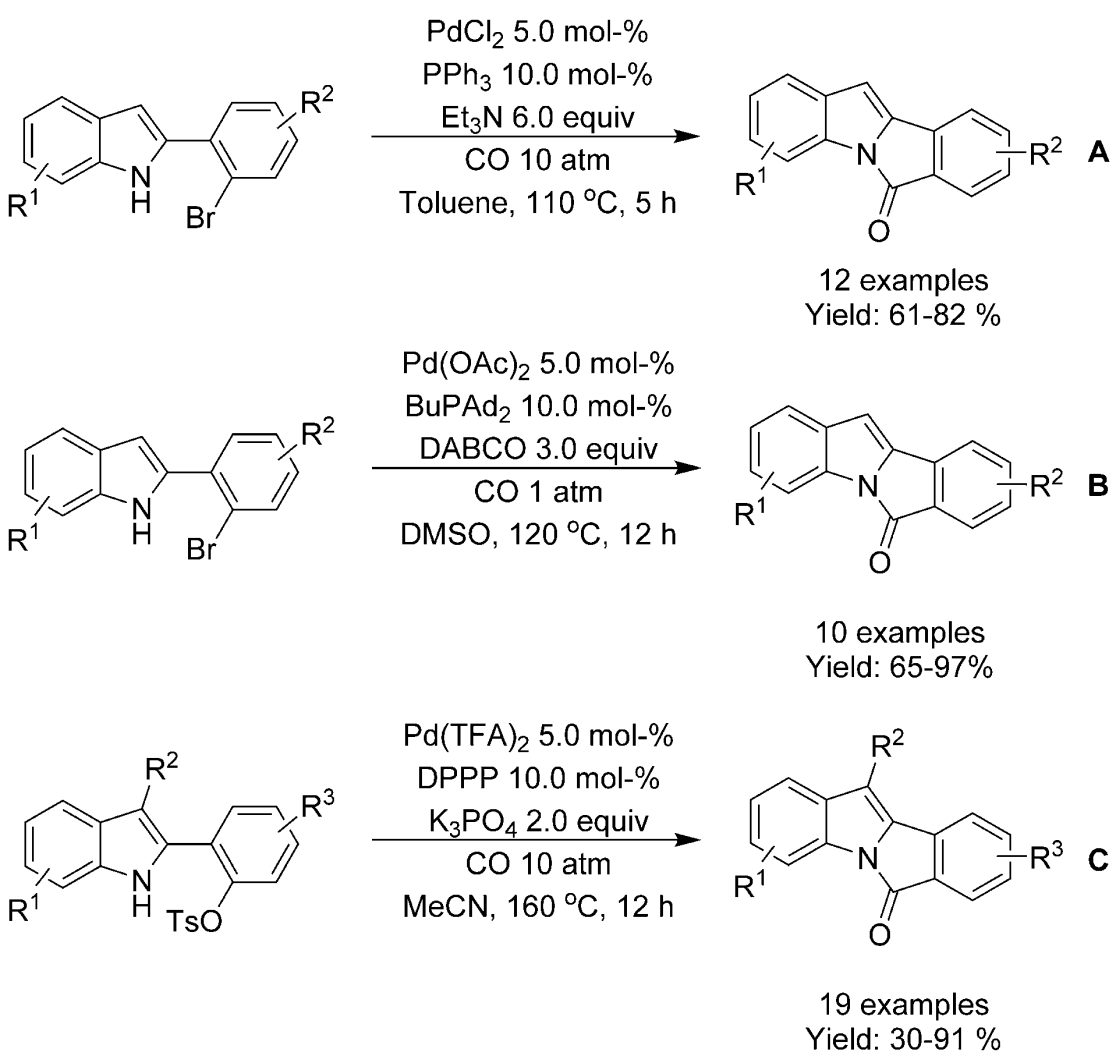
Synthesis of isoindolo[2,1‐*a*]indol‐6‐one derivatives via palladium catalyzed intramolecular carbonylation.

A variety of methods for intramolecular carbonylation of 2‐aryl indoles to isoindolo[2,1‐*a*]indol‐6‐ones have been reported. Cho^[149]^ described a combination of palladium(II) chloride with triphenyl phosphine as catalyst under 10 atm of CO and trimethylamine as base (Scheme 72 A), whereas Guo et al.^[150]^ utilized palladium(II) acetate with *n*‐butyl‐di(1‐adamantyl)phosphine (BuPAd_2_) and 1,4‐diazabicyclo[2.2.2]octane (DABCO) as the base at atmospheric carbon dioxide pressure (Scheme 72 B). Zhou^[151]^ expanded a similar methodology to 2‐(1*H*‐indol‐2‐yl)phenyl tosylates with palladium(II) trifuoroacetate as the catalyst, 1,3‐bis(diphenylphospine)propane (DPPP) as ligand and inorganic tripotassium phosphate as the base instead of commonly utilized organic bases (Scheme 72 C).

**Scheme 73:**
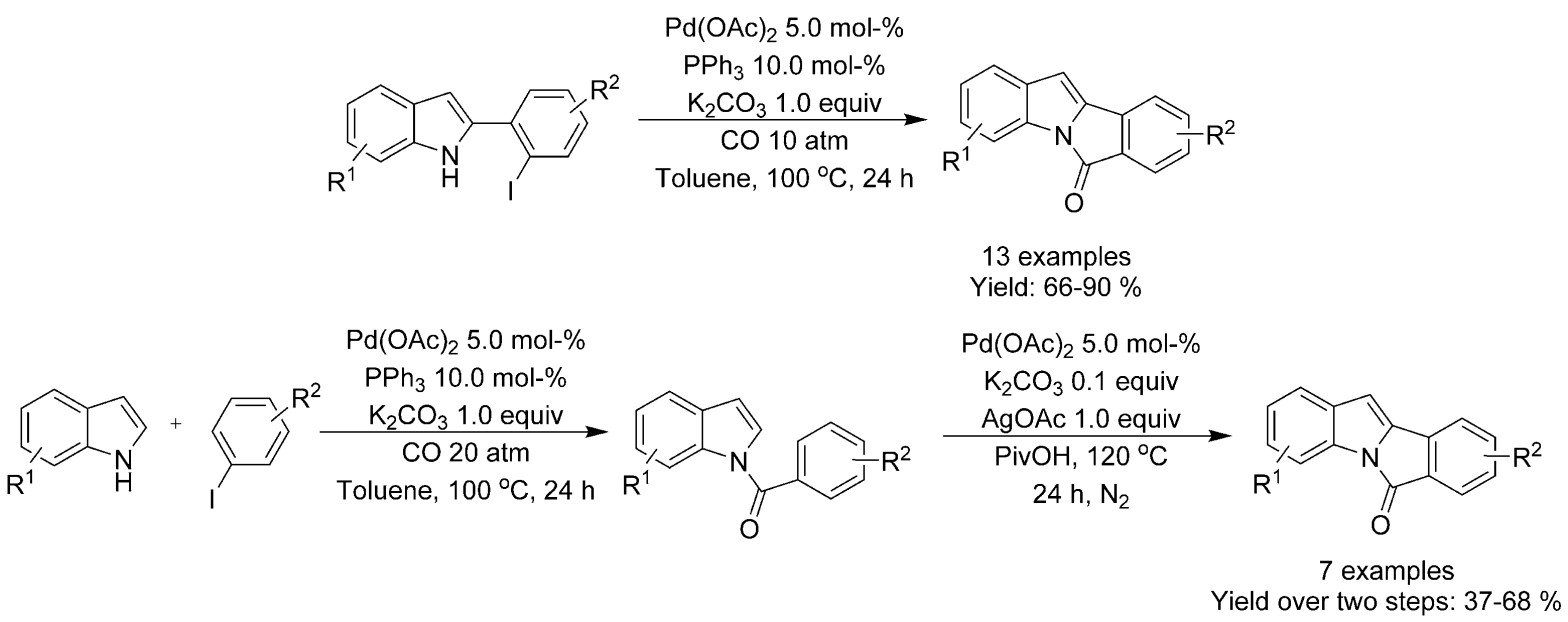
Synthesis of 6*H*‐isoindolo[2,1‐α]indol‐6‐ones by means of intramolecular carbonylation or by intermolecular carbonylation/intramolecular dehydrogenative‐coupling.

Huang^[152]^ described two methods for the preparation of 6*H*‐isoindolo[2,1‐α]indol‐6‐ones either by means of intramolecular carbonylation of 2‐(2‐iodophenyl)‐1*H*‐indoles or by intermolecular carbonylation of indoles with iodobenzenes followed by intramolecular dehydrogenative‐coupling (Scheme 73). For the intramolecular carbonylation, 4‐subtituted (R^1^) indoles resulted in lower yields with 2‐(2‐iodophenyl)‐4,5‐dichloro‐1*H*‐indole producing only trace amounts of the functionalized isoindolinone. While sequential two‐step intermolecular carbonylation of indoles with iodobenzenes/intramolecular dehydrogenative‐coupling was not utilized in one‐pot fashion, it was proven to be compatible with aldehyde and ester derivatized indoles.

**Scheme 74:**
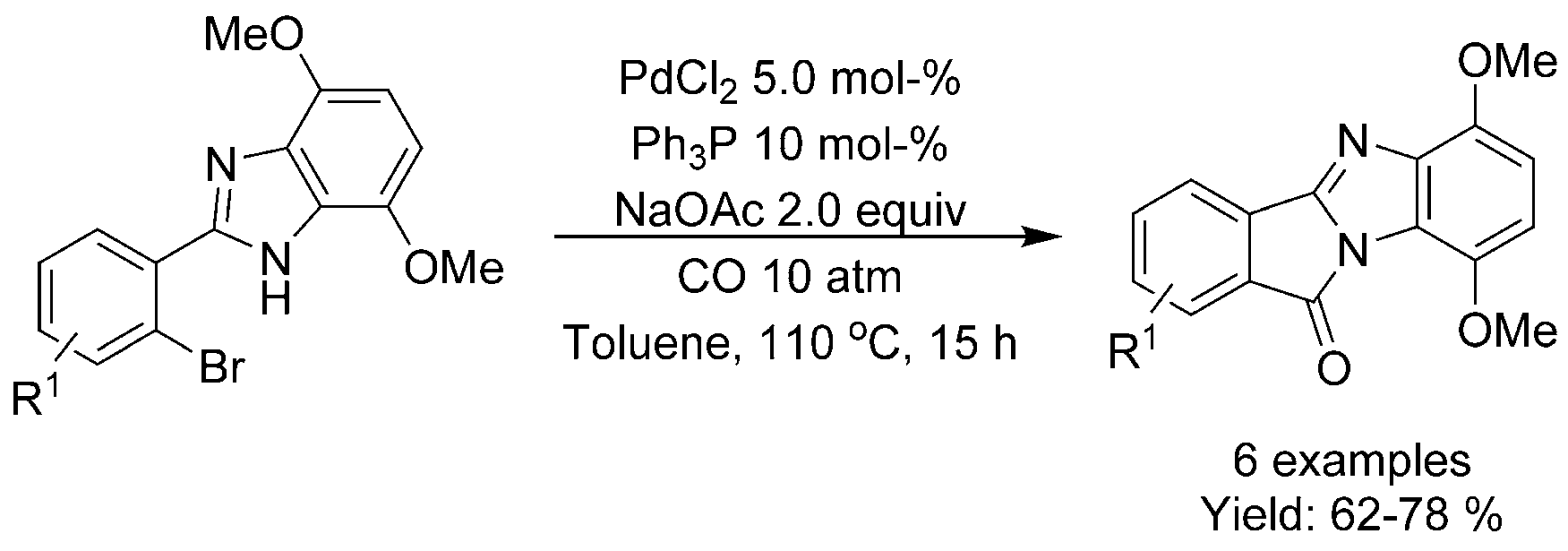
Palladium catalyzed intramolecular carbonylation 2‐(2‐bromoaryl)‐4,7‐dimethoxybenzimidazoles.

Cho extended their previous work of 2‐aryl indoles^[149]^ carbonylation (Scheme 64 A) to intramolecular carbonylation of 2‐(2‐bromovinyl)‐4,7‐dimethoxybenzimidazoles and 2‐(2‐bromoaryl)‐4,7‐dimethoxybenzimidazoles (Scheme 74).^[153]^ While the work was mainly focused on 2‐(2‐bromovinyl)‐4,7‐dimethoxybenzimidazole carbonylation, a few examples leading into polycyclic isoindolinone bearing compounds were described.

**Scheme 75:**
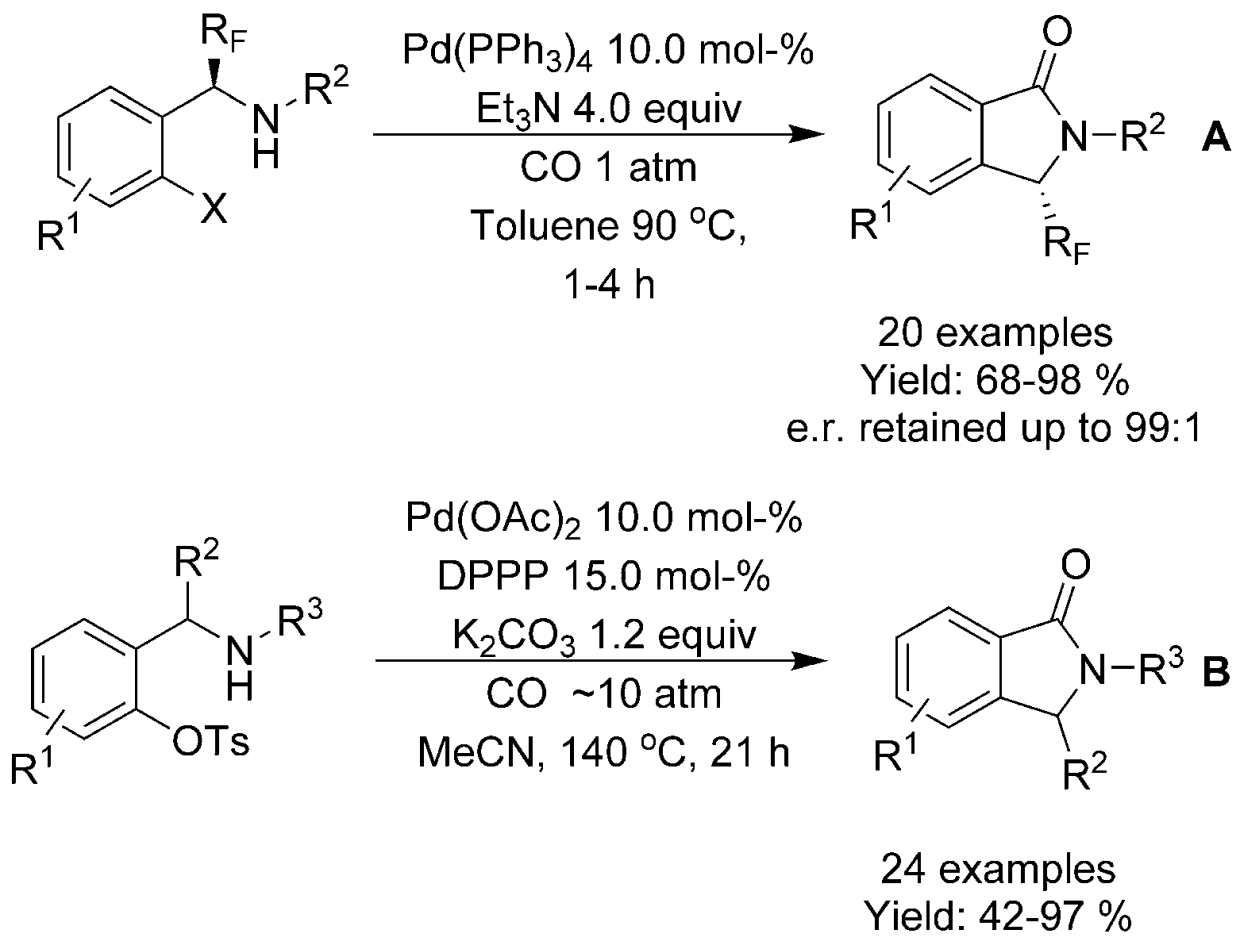
Palladium‐catalyzed aminocarbonylation of 2‐iodobenzylamines and 2‐(aminomethyl)aryl tosylates.

Asymmetric synthesis of fluorinated isoindolinones by palladium‐catalyzed intramolecular aminocarbonylation of the chiral‐*α*‐fluoroalkyl‐2‐iodobenzylamines was described by Bario et al. (Scheme 75 A).^[154]^ In general, the best yields were achieved with *tert*‐butoxycarbonyl or benzyloxycarbonyl derivatized amines and “free” secondary amines resulted in slightly lower yields. Especially fluoride and trifluoromethyl substituents (R^1^) resulted in significant erosion of the optical purity, probably due to a base‐mediated *anti* β‐hydride elimination process as suggested by the authors. The degradation of the optical purity was correlated with p*K*_a_ of the base and it was demonstrated that the racemization process could be minimized with weaker bases. Instead of 2‐halobenzylamines, Zhou^[155]^ utilized 2‐(aminomethyl)aryl tosylates as substrates for intramolecular aminocarbonylation. Palladium(II) acetate and DPPP were used as catalyst/ligand combination in the presence of potassium carbonate as a base at 10 atm of carbon monoxide and significantly elevated temperatures were required for the reaction to proceed (Scheme 75 B). In general, the halogenated and ester bearing substrates resulted in moderate yields, whereas other tested substrates resulted in good to high yields.

**Scheme 76:**
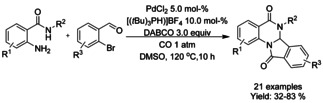
Synthesis fused polycyclic isoindolinone derivatives via palladium catalyzed carbonylation.

Palladium‐catalyzed one‐pot multicomponent cascade reaction of 2‐aminobenzamides with 2‐bromobenzaldehydes and carbon monoxide generating 6,6*a*‐dihydroisoindolo[2,1‐*a*]quinazoline‐5,11‐diones was described by Guo et al. (Scheme 76).^[156]^ The reaction proceeds via initial cyclocondensation of the benzamide and benzaldehyde derivatives, followed by cyclocarbonylation. In general, the reaction can be applied to a number of readily available substrates in high yields with only quinoline analog of benzamide having significant detrimental effect to the yield.

**Scheme 77:**
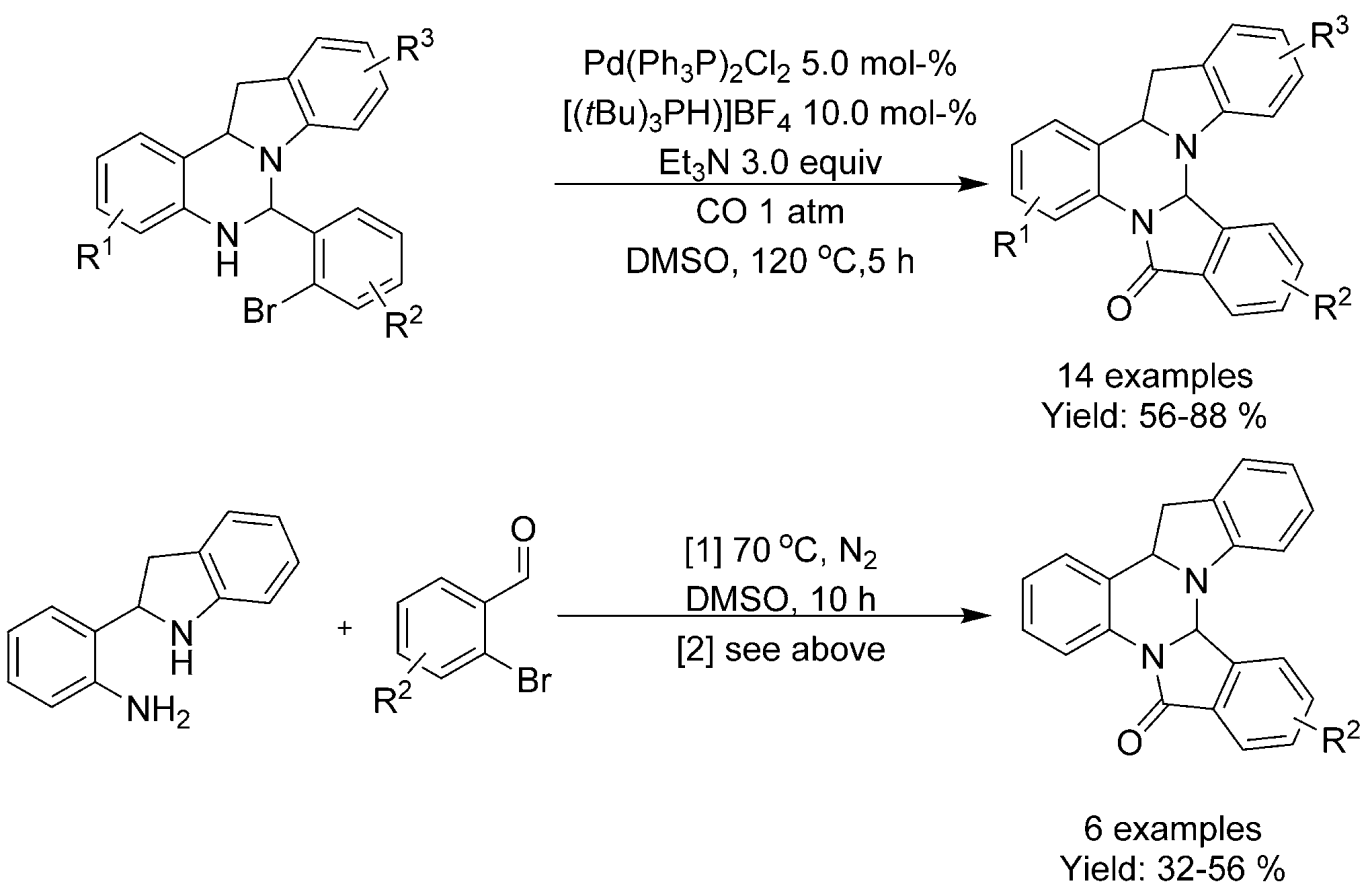
Synthesis of indolo[1,2‐*c*]isoindolo[2,1‐*a*]quinazolin‐5(16*aH*)‐one derivatives by means of palladium catalyzed cyclocarbonylation.

Based on their previous research,[^142^, ^156^] Guo et al.^[157]^ applied slightly modified conditions to the synthesis of indolo[1,2‐*c*]isoindolo[2,1‐*a*]quinazolin‐5(16*aH*)‐one derivatives in moderate to high yields via palladium catalyzed cyclocarbonylation of 6‐(2‐bromoaryl)‐5,6‐dihydroindolo[1,2‐*c*]quinazoline derivatives (Scheme 77). The main limitation, was found to be fluoro and nitro substituents (R^2^) in the pendant aryl moiety. The above‐mentioned products were also obtained directly by means of a one‐pot two‐step tandem reaction in modest yields. 2‐(1*H*‐Indol‐2‐yl)anilines and 2‐bromobenzaldehydes were subjected to initial cyclocondensation generating the 6‐(2‐bromoaryl)‐5,6‐dihydroindolo[1,2‐*c*]quinazoline precursors followed by the cyclocarbonylation.

**Scheme 78:**
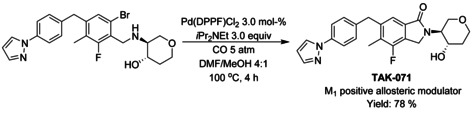
Late‐stage carbocyclization to isoindolinone ring system in scale‐up synthesis of **TAK‐071**.

As an example, **TAK‐071** is an M_1_ positive allosteric modulator pharmacophore developed by Takeda Pharmaceutical Company Limited, bearing an isoindolin‐1‐one ring system. In the scale‐up of the synthesis, a late stage carbocyclization was utilized. The benzyl amine derivative was carbonylated under 5 atm of carbon dioxide utilizing 1,1’‐ferrocenediyl‐bis(diphenylphosphine) (DPPF) ligated palladium(II) chloride as a catalyst in the presence of an organic base (Scheme 78).^[158]^

#### Pd‐catalyzed dearomative carbonylation

**Scheme 79:**
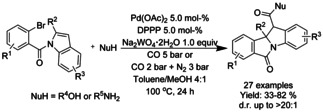
Synthesis of isoindolo[2,1‐*a*]indol‐6‐one derivatives via palladium catalyzed intramolecular carbonylation.

Wu^[159]^ described the first example of palladium‐catalyzed dearomative carbonylation of *N‐*(2‐bromobenzoyl)indoles leading to a variety of polycyclic spiro compounds containing the isoindolinone skeleton. Here, palladium(II) acetate and DPPP as the catalyst/ligand combination with sodium tungstate as a base yielded the best results. The optimized conditions were successfully applied to a variety of anilines and alcohols as nucleophiles (Scheme 79).

#### Pd‐catalyzed carbonylation with CO‐surrogates

**Scheme 80:**
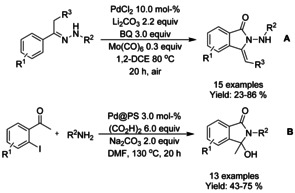
Molybdenum(0) hexacarbonyl and oxalic acid as CO‐surrogates for palladium catalyzed carbonylation.

Carbon monoxide is one of the cheapest C_1_ sources available, but due to its hazardous properties, such as high toxicity and flammability, a constant use of CO detector is required for safety. This can limit its use especially in smaller and academic research laboratories. Wu^[160]^ reported a palladium catalyzed C−H carbonylation protocol of ketimines utilizing nontoxic solid molybdenum(0) hexacarbonyl (Mo(CO)_6_) as an CO‐surrogate (Scheme 80 A). In this manner, 3‐methyleneisoindolin‐1‐ones were prepared in moderate to good yields. The best results were obtained by the use of benzohydrazine directing group but *O*‐methylhydroxylamine and acetohydrazide were also found to be viable directing groups albeit at best in moderate yields. Das^[161]^ described a polystyrene‐supported palladium (Pd@PS) nanoparticle catalyzed multicomponent synthesis of 3‐hydroxy‐3‐methyl isoindolinones from 2’‐iodoacetophenones and primary benzyl amines utilizing oxalic acid as CO‐surrogate (Scheme 80 B). The use of halogenated and trifuoromethoxy derivatized benzyl amines resulted in moderate yields.

**Scheme 81:**
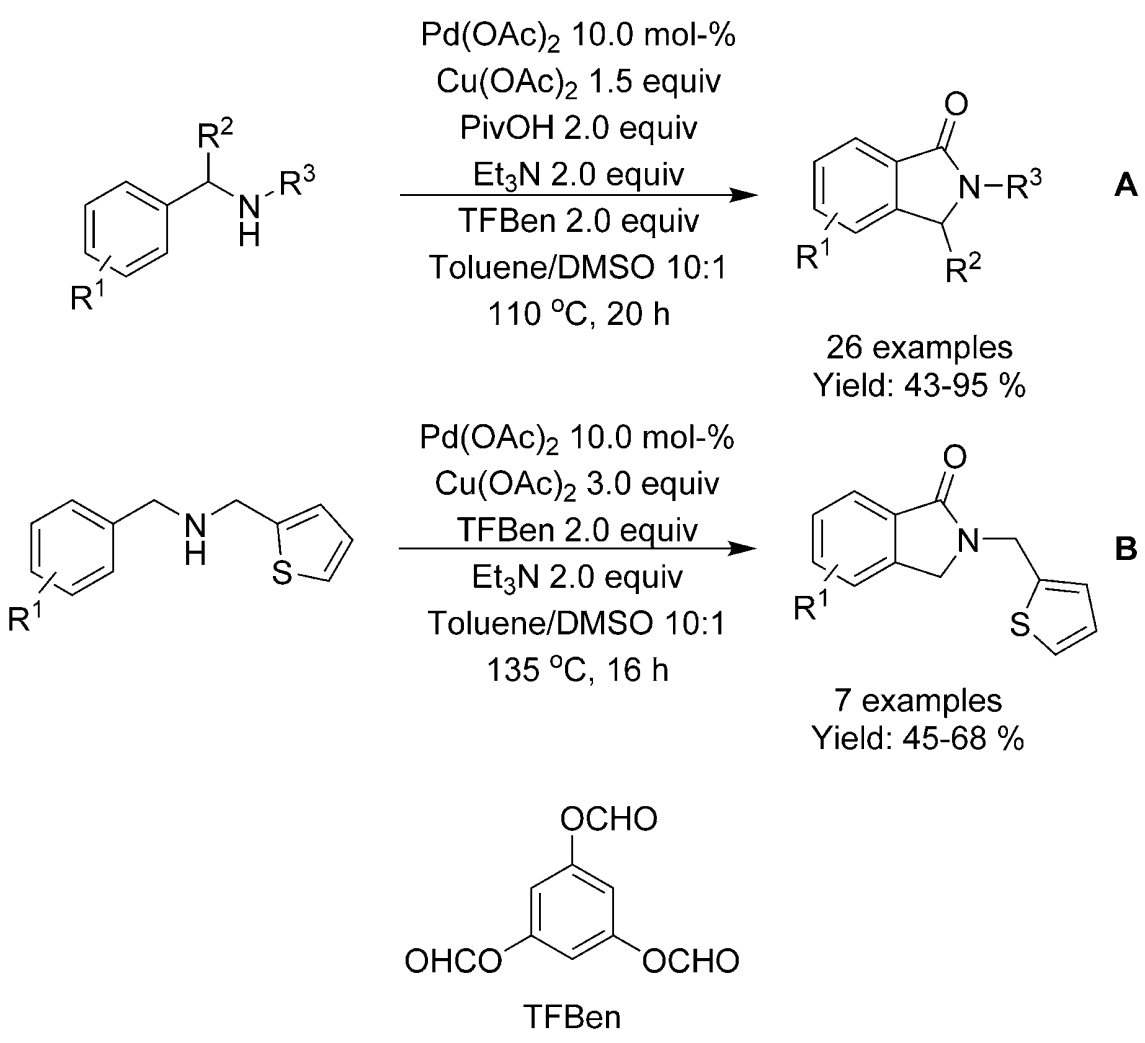
TFBen as CO‐surrogate in palladium catalyzed carbonylation.

Benzene‐1,3,5‐triyl triformate (TFBen) was applied as a CO‐surrogate by both Fu et al.^[162]^ and Wu^[163]^ to palladium catalyzed carbonylation of benzyl amines via oxidative C−H functionalization. Fu et al. applied the methodology to broad range of benzyl amines yielding the respective 3 and *N*‐substituted isoindolinones in moderate to good yields (Scheme 81 A). From the tested substrates, only cyano and bromo substituents resulted in lower yields and 3,4‐disubtituted benzyl amines lead into mixtures of isomeric structures. Wu described a route to N‐derivatized isoindolinones with a pendant 2‐methyl thiophene as a part of site‐selective carbonylative methodology (Scheme 81 B).

**Scheme 82:**
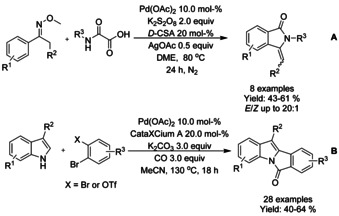
Synthesis of isoindolinones using *N*‐monosubstituted oxamic acids or CO generated from mixture of sulfuric and glyoxylic acid.

Wang^[164]^ reported a palladium‐catalyzed *ortho*‐amidation of ketoximes with oxamic acids as the carbonyl and nitrogen source. When the methodology is applied to N‐monosubstituted oxamic acids, the *ortho*‐amidation is followed by intramolecular cyclization generating 3‐alkylidene isoindolinones (Scheme 82 A). Čarný et al.^[165]^ described a multicomponent tandem palladium catalyzed carbonylation and C−C cross‐coupling via C−H activation between indoles, 1,2‐dibromobenzenes and carbon monoxide via CO‐surrogate (Scheme 82 B). Carbon monoxide utilized in the reaction was generated in a separate vial from glyoxylic acid and concentrated sulfuric acid. The reaction is described to proceed via in situ formed *N‐*(2’‐bromoaroyl)‐indole to 6*H*‐isoindolo[2,1‐*a*]indol‐6‐ones in moderate to good yields. No reaction was observed in the cases of 3‐acetylindole and 5‐nitroindole. Unsymmetric 1,2‐dibromobenze derivatives led into mixtures of isometric structures which could be avoided by the use 2‐bromo‐phenyl trifluoromethane‐sulfonates.

#### Pd‐catalyzed asymmetric carbonylation

**Scheme 83:**
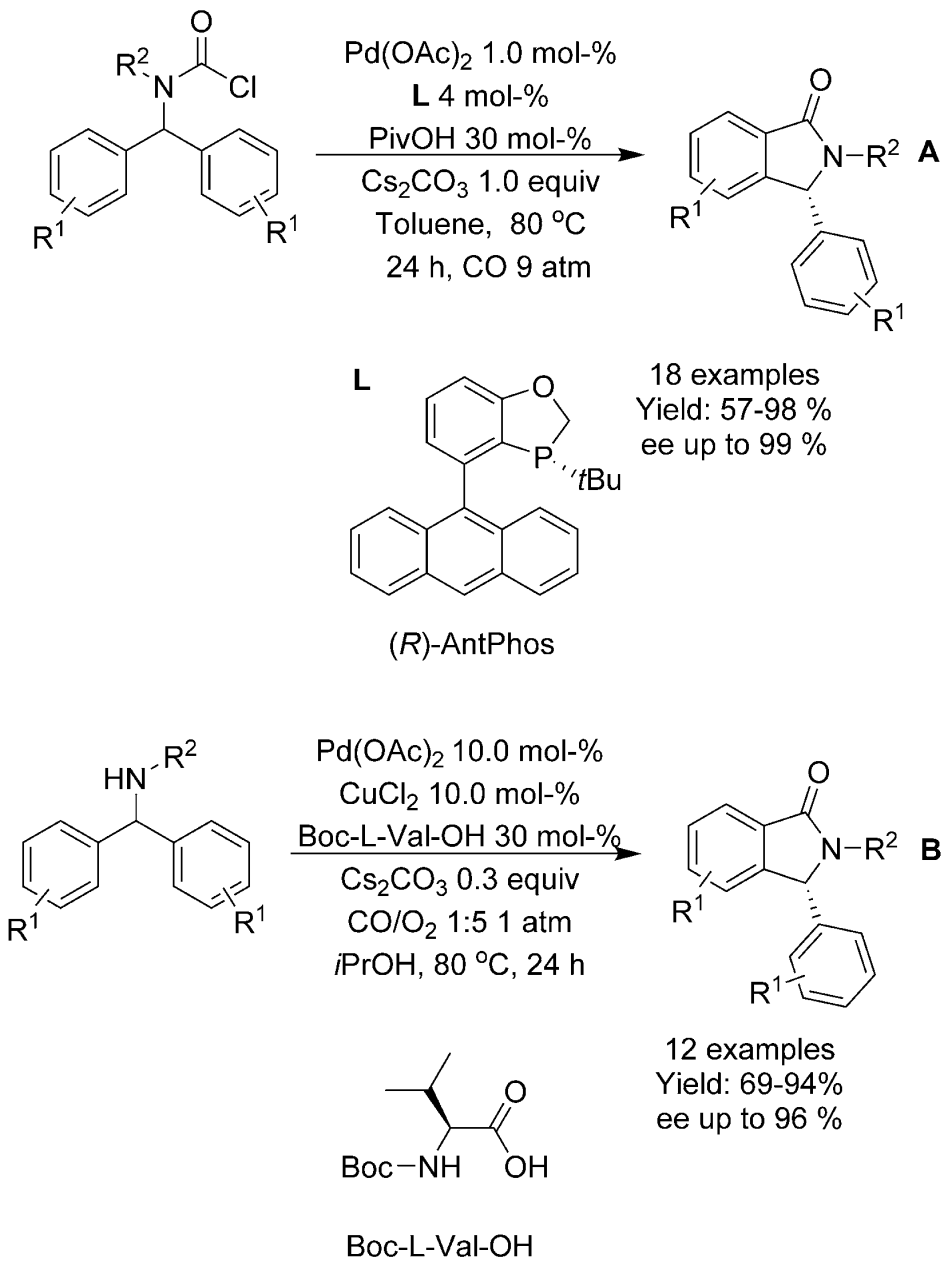
Asymmetric synthesis of isoindolinones via carbonylation based strategies.

Enantioselective preparation of chiral isoindolines via palladium‐catalyzed carbamoylation by means of C−H functionalization was reported by Tang (Scheme 83 A).^[166]^ A chiral monophosphorus ligand (*R*)‐AntPhos was utilized as the ligand under 9 atm of carbon monoxide yielding a variety of chiral isoindolines in high enantiomeric excess and moderate to high yields. Long alkyl chains and bulky substituents in the amine as well as trifuoromethyl substituents in the aryl groups resulted in lower yields. An enantioselective oxidative carbonylation process of activated benzylic amines was described by Wu (Scheme 83 B).^[167]^ A bimetallic Pd/Cu‐based catalyst system in the presence of mono‐N‐protected amino acid ligands catalyzed a C−H carbonylation of prochiral arylsulfonamides via desymmetrization process. The reaction provides a facile stereoselective construction of isoindoline‐1‐ones in good yields and enantioselectivities under atmospheric pressure of CO/O_2_ in the ratio of 1:5.

### Rhodium catalyzed carbonylation reactions

#### Rh‐catalyzed carbonylation via C−H functionalization

**Scheme 84:**
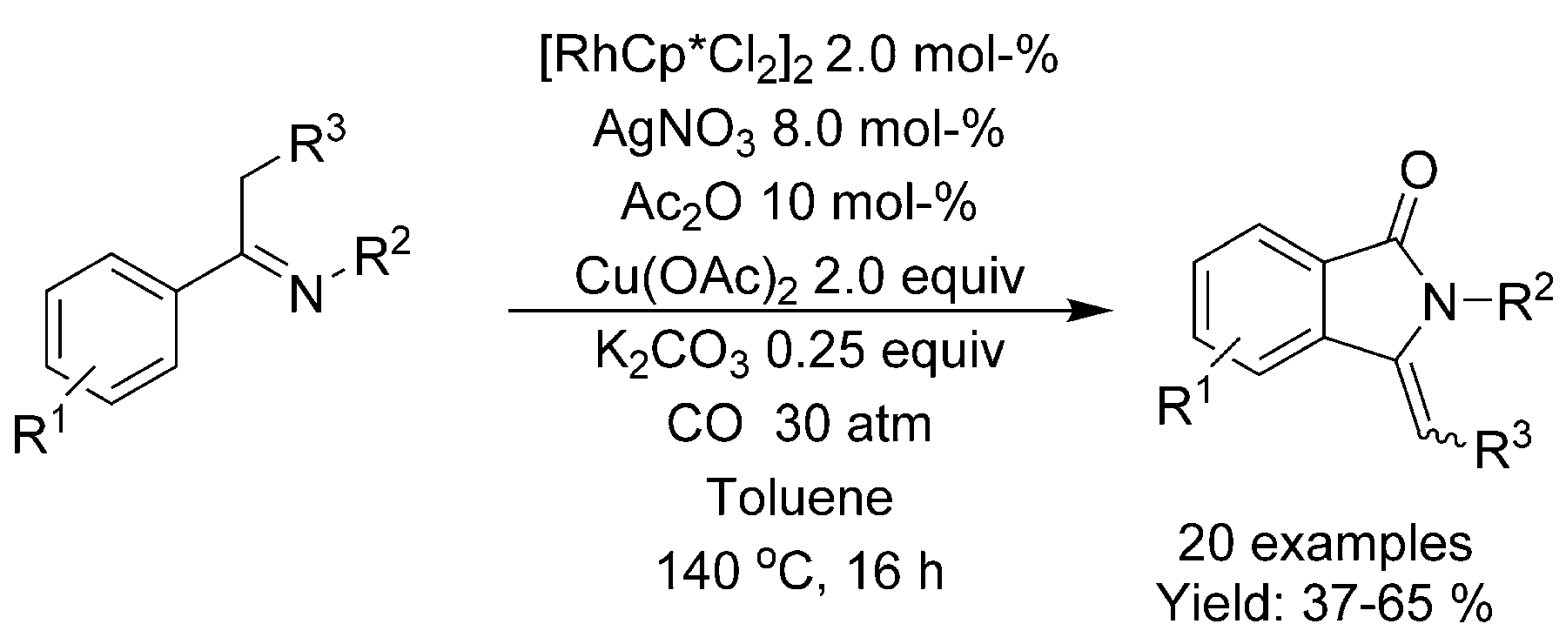
A rhodium catalyzed oxidative carbonylation of ketimines.

H. Huang^[168]^ described a rhodium catalyzed oxidative carbonylation of ketimines under 30 atm of carbon monoxide at significantly elevated temperatures, yielding 3‐alkylideneisoindolinones in moderate yields (Scheme 84).

**Scheme 85:**
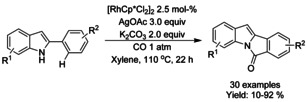
Rhodium‐catalyzed a synthesis of 6*H*‐isoindolo[2,1‐*a*]indol‐6‐ones via carbonylation.

Q. Huang et al.^[169]^ presented a rhodium‐catalyzed synthesis of 6*H*‐isoindolo[2,1‐*a*]indol‐6‐one derivatives via oxidative N−H/C−H carbonylation of 2‐arylindoles under atmospheric CO‐pressure (Scheme 85). The optimized conditions were applied to a broad substrate scope generating the title compounds in moderate to very high yields. Substrates with cyano or nitro groups at the indole (R^1^) or iodo substituent at the pendant aryl (R^2^) group resulted in lowest yields.

#### Rh‐catalyzed carbonylation via C−X functionalization

**Scheme 86:**
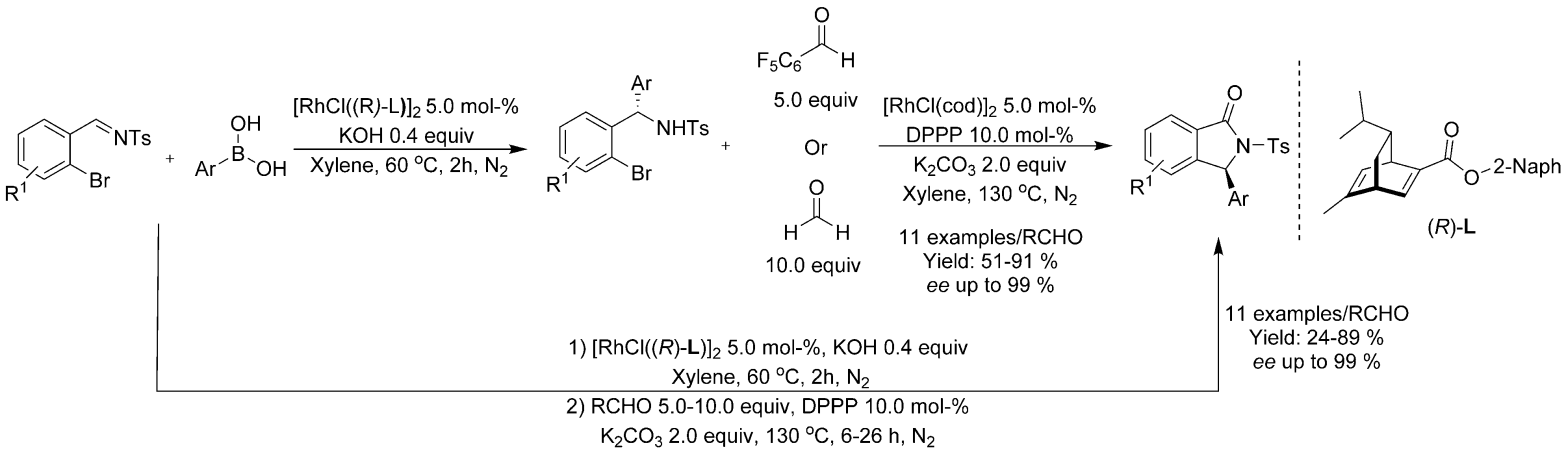
CO‐surrogate based Rh^I^‐catalyzed asymmetric synthesis of 3‐substituted isoindolinones.

Morimoto^[170]^ reported a CO‐surrogate based Rh^I^‐catalyzed asymmetric synthesis of 3‐substituted isoindolinones via aminocarbonylation reaction in moderate to good yield and very high *ee* (Scheme 86). First the rhodium catalyzed carbonylation of chiral *N*‐tosylated‐2‐halobenzylamines utilizing either pentafluorobenzaldehyde or formaldehyde as CO‐surrogate was optimized. In most cases the yield of the product was dependent on the CO‐surrogate utilized, with pentafluorobenzaldehyde achieving higher isolated yields in comparison to formaldehyde. Subsequently, an asymmetric one‐pot two‐step reaction with Rh^I^‐catalyst was described, starting from coupling of prochiral aldimines and arylboronic acids followed by subsequent carbonylation reaction, yielding the title compounds close to similar yields and enantiomeric excesses when compared to those of the single step carbonylation.

#### Rh‐catalyzed carbonylation with CO‐surrogates

**Scheme 87:**
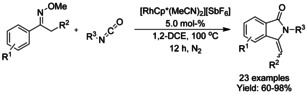
Rhodium catalyzed *ortho*‐amidation of ketoximes isocyanates.

Zhou et al.^[171]^ reported [RhCp*(MeCN)_2_][SbF_6_]‐complex as a catalyst for *ortho*‐amidation of ketoximes via C−H functionalization with isocyanates generating 3‐alkylideneisoindolinones (Scheme 87). The reaction exhibited a high regioselectivity and functional‐group tolerance allowing for a broad substrate scope. While the reaction proceeds without the use of additives and does not produce any significant environmentally hazardous waste, it is operated in 1,2‐dichloroethane. Main challenges for the reaction proved to be *ortho*‐halogenated ketoximes and bulkier isocyanates.

### Ruthenium catalyzed carbonylation reactions

#### Ru‐catalyzed carbonylation with CO‐surrogate

**Scheme 88:**
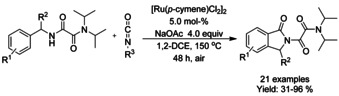
A ruthenium‐catalyzed intramolecular C−H carbonylation.

Han et al.^[172]^ developed a ruthenium‐catalyzed intramolecular C−H carbonylation for synthesis of isoindolin‐1‐one derivatives from oxalyl amide‐protected benzylamines, using isocyanate as a CO‐surrogate (Scheme 88). Variously substituted benzylamines were well tolerated, affording the isoindolin‐1‐ones in moderate to very high yields. Based on the substrate scope, 3‐ and/or 4‐substituted and unsubstituted benzyl amines resulted in lowest yields.

### Nickel catalyzed carbonylation reactions

#### Ni‐catalyzed carbonylation via C−X functionalization

**Scheme 89:**
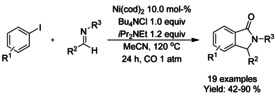
Nickel‐catalyzed carbonylative synthesis of isoindolinones.

A multicomponent nickel catalyzed carbonylative synthesis of isoindolinones from aryl iodides, aldimines and carbon monoxide in the presence of a chloride salt was reported by Tjutrins et al. (Scheme 89).^[173]^ The reaction was postulated to proceed via the initial formation of *N*‐acyl iminium chloride salts, followed by a spontaneous nickel‐catalyzed cyclization yielding the isoindolinone derivatives in moderate to high yields. Cyano‐substituted aryl iodides as well as with bulky N‐substituents (R^3^) in the aldimines were found to result in lower yields.

### Cobalt catalyzed carbonylation reactions

#### Co‐catalyzed carbonylation with CO‐surrogates

**Scheme 90:**
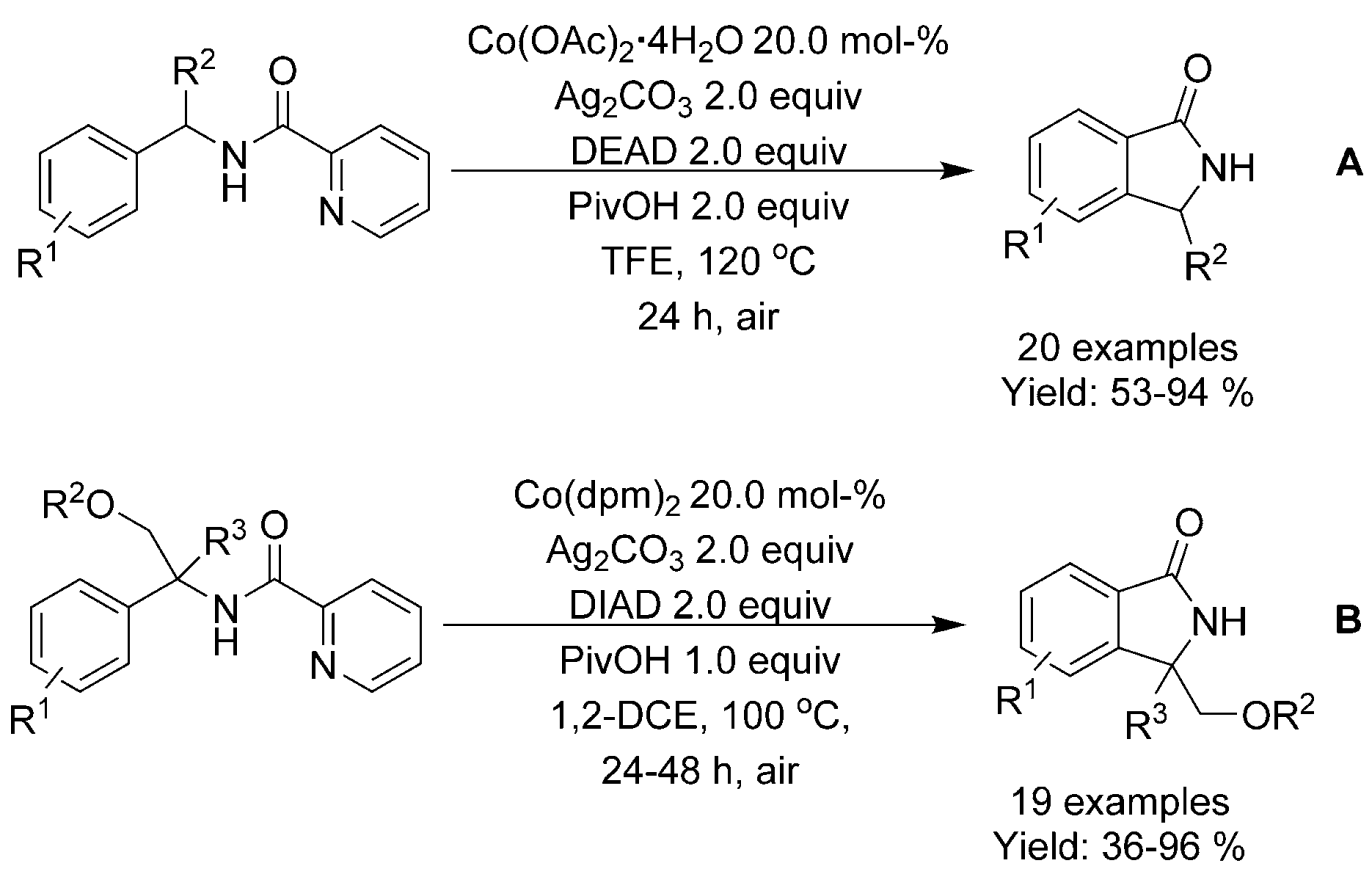
Cobalt‐catalyzed oxidative C−H carbonylation using CO‐surrogates.

Zhong^[174]^ and Grigorjeva^[175]^ both applied diethyl azodicarboxylate (DEAD) and diisopropyl azodicarboxylate (DIAD) as CO‐surrogates in the cobalt‐catalyzed oxidative C−H carbonylation of benzyl amines, using picolinamide as a traceless directing group (Scheme 90 A and B, respectively). In both cases, a variety of N‐underivatized isoindolinones were prepared in moderate to high yields, with marginal differences between electron withdrawing and donating substituents at the arene (R^1^). When chiral benzylic amines were used as substrates, the enantiopurity of the chiral center was maintained and Zhong demonstrated how the developed method could be applied to the synthesis of (+)‐garenoxacin.

## Lactamization via Transition Metal Catalyzed Condensation or Addition Reactions

### Zinc, scandium and indium catalyzed reactions

#### Zn, Sc and In‐catalyzed Mannich reaction‐lactamization

**Scheme 91:**
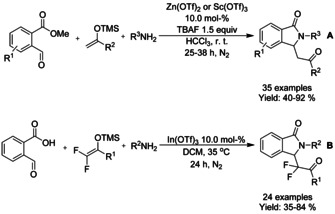
Lewis acid catalyzed three‐component synthesis of isoindolinones via Mannich/lactamization cascade.

Multicomponent Lewis acid catalyzed Mannich/lactamization cascade for synthesis of various isoindolinones was described by both Singh^[176]^ and Cai^[177]^ (Scheme 91 A and B, respectively). Singh applied *o*‐formyl methylbenzoates, trimethylsilyl enol ethers and aniline derivatives in the presence of zinc(II) or scandium(III) triflate catalysts to generate a broad scope of isoindolinones in moderate to high yields. Here the main difficulties arose from *ortho*‐substituted anilines and anilines bearing multiple electron donating groups. Cai applied indium(III) triflate as a catalyst for 2‐formylbenzoic acids, primary amines and difluoroenoxysilanes to afford N‐substituted 3‐oxoisoindoline‐1‐difluoroalkyl derivatives in good yields with exception of *ortho*‐substituted anilines.

#### Zn, Sc and In‐catalyzed Strecker reaction‐lactamization

**Scheme 92:**
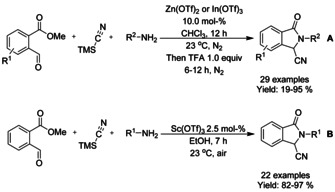
Metal triflate catalyzed Strecker reaction/lactamization cascades.

Singh^[178]^ applied Zn^II^ and In^III^ triflates (Scheme 92 A) and Cai^[179]^ Sc^III^ triflate (Scheme 92 B) to Strecker reaction/lactamization cascade, producing 3‐cyano derivatized isoindolinones in good to excellent yields. In the method reported by Singh, only very bulky *ortho*‐substituted anilines were reported to result in low isolated yields, whereas in the case of scandium(III) triflate catalyst only *para*‐nitro aniline and *tert*‐butyl amine were not tolerated.

#### Zn and In‐catalyzed allylation‐lactamization

**Scheme 93:**
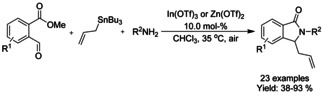
Catalytic homoallylation and allylation cascades for isoindolinone synthesis.

Lewis acid catalyzed one‐pot cascade was optimized for In^III^ and Zn^II^ triflate catalysts utilizing tin based allylation reagent (Scheme 93).^[180]^ In accordance with the Lewis acid catalyzed Mannich and Strecker reaction based methodologies (vida supra), the substituents at *o*‐formyl benzoates had minimal effect on the yield of the reaction while bulky O‐substituted anilines had a significantly detrimental effect on the yield.

### Ytterbium catalyzed reactions

#### Yb‐catalyzed addition‐lactamization

**Scheme 94:**
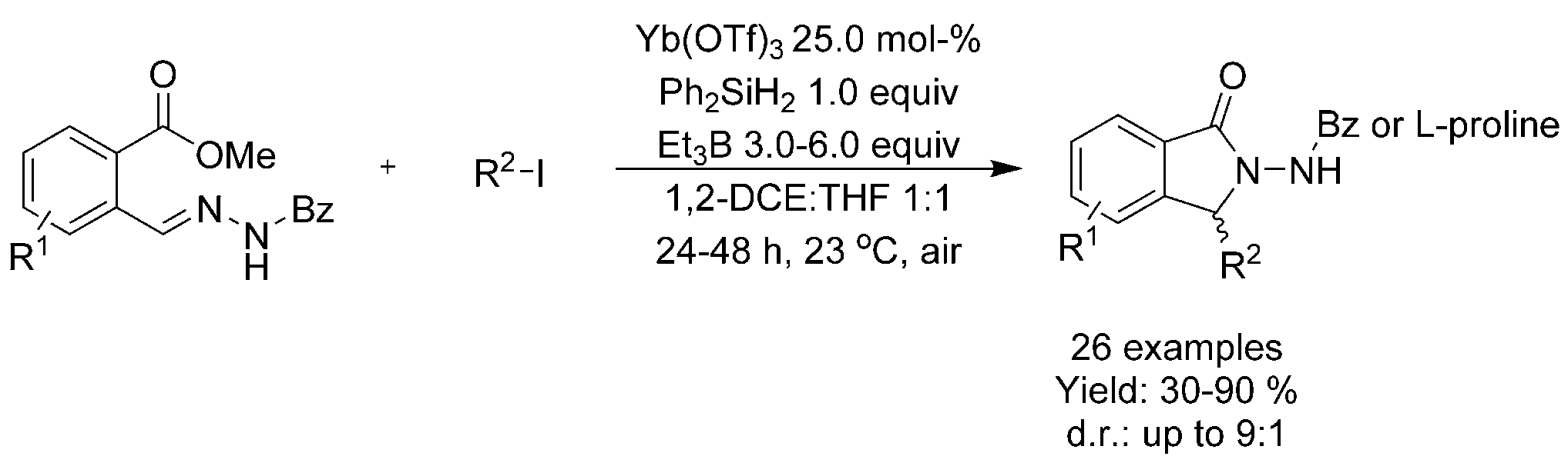
Ytterbium(III) triflate catalyzed radical addition forming 3‐substituted isoindolinones.

Jang^[181]^ described an ytterbium(III) triflate catalyzed radical addition involving 2‐formylbenzoate‐derived hydrazone, isopropyl iodide, diphenylsilane and triethylborane (Scheme 94). In this manner, a variety of 3‐substituted isoindolinones we formed in moderate to high yields, with only amine‐substituted or 3‐substituted 2‐formylbenzoates leading into diminished yields. Also, switching from benzoyl hydrazones to l‐proline analog the respective chiral 3‐subtituted isoindolinones were produced with diastereomeric ratios up to 9:1.

### Cobalt catalyzed reactions

#### Co‐catalyzed Pinner–Dimroth reaction

**Scheme 95:**
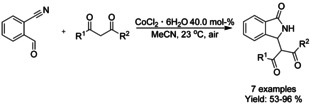
Cobalt(II) chloride catalyzed Pinner‐Dimroth tandem reaction.

Shi^[182]^ described a cobalt(II) chloride catalyzed Pinner–Dimroth tandem reaction between 2‐cyanobenzaldehyde and 1,3‐dicarbonyl compounds yielding 3‐subtituted isoindolinones in moderate to high yields, albeit at high catalyst loading and limited substrate scope (Scheme 95).

## Nickel Catalyzed Reactions

### Ni‐catalyzed homoallylation reaction

**Scheme 96:**
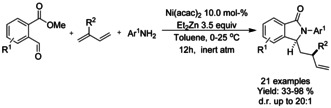
Catalytic homoallylation and allylation cascades for isoindolinone synthesis.

Singh presented a homoallylation and allylation based cascades for isoindolinone synthesis via in situ generated imines from primary amines and *o*‐formyl benzoates. The homoallylation was achieved utilizing a Ni(acac)_2_ catalyst and stoichiometric amount of diethylzinc under inert atmosphere forming the 3‐substituted diastereomeric isoindolinones in moderate to good yields. In general, steric hindrance near the C3‐position led to the best diastereomeric ratios albeit at the expense of yield (Scheme 96).^[183]^

### Copper catalyzed reactions

#### Cu‐catalyzed synthesis of isoindolinones using hypervalent iodine reagents

**Scheme 97:**
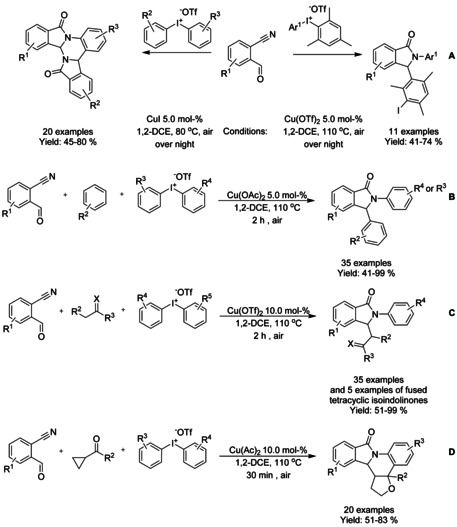
Synthesis of isoindolinones using hypervalent iodide compounds.

Li^[184]^ reported a number of copper catalyzed methods for isoindolinone scaffolds from 2‐formylbenzonitriles and diaryliodonium salts (Scheme 86, see later). The reactions proceed via copper catalyzed electrophilic activation of the cyano group and undergo C−N/C−C tandem cyclizations to afford the isoindolinone scaffolds. By selecting suitable catalyst and hypervalent iodine reagent, a divergent cyclization was initially developed forming either 2,3‐diarylisoindolinone derivatives or polycyclic compounds containing two isoindolinone scaffolds in moderate to good yields (Scheme 97 A).^[184a]^ The methodology for 2,3‐diaryl substituted isoindolinone synthesis was further extended to a multicomponent reaction allowing for more flexible synthesis of various isoindolinone derivatives (Scheme 97 B).^[184b]^ Unsymmetrical diaryliodonium salts could be replaced by combination of symmetric diaryliodonium salts with a suitable arene. The multicomponent methodology was further expanded to encompass alkyl aryl ketones and α‐methylstyrene instead of arenes to generate isoindolinone derivatives and dihydroisoindolo[2,1‐*a*]quinolin‐11(5*H*)‐ones in moderate to excellent yields (Scheme 97 C).^[184c]^ Finally, the three‐component cascade cyclization methodology was applied to cyclopropyl ketones constructing fused polycyclic isoindolinones. Here the final step involves a hetero‐[4+2]‐cycloaddition reaction between an *N*‐aryl nitrilium cation and dihydrofuran derivative formed from the cyclopropyl ketone (Scheme 97 D).^[184d]^

#### Cu‐catalyzed in situ imine formation/alkynylation/lactamization cascade

**Scheme 98:**
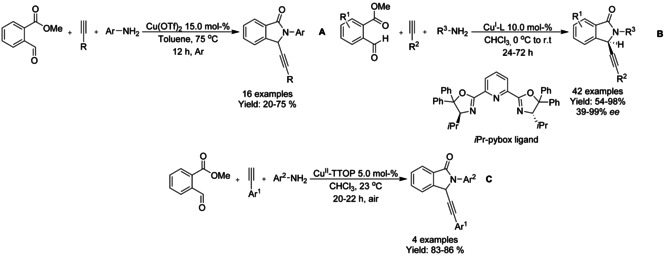
Copper catalyzed in situ imine formation/alkynylation/lactamization cascades.

Sun et al.^[185]^ reported a copper catalyzed multicomponent reaction for the synthesis of 2,3‐disubstituted isoindolinones utilizing in situ imine formation/alkynylation/lactamization cascade with methyl 2‐formylbenzoates, anilines and alkynes as the reactants (Scheme 98 A). While a number of isoindolinones can be formed in this manner in low to good yields, the isolated yields are highly dependent on the alkynes utilized as substrates. In addition, similarly above‐mentioned metal triflate catalyzed methods the *ortho*‐substituted anilines resulted at best in trace amounts of product. In 2014, Singh^[186]^ reported for the first time a highly enantioselective Cu‐catalyzed alkynylation/lactamization cascade of *o*‐formyl methyl benzoates with aromatic amines and terminal alkynes using a *i*Pr‐pybox ligand (Scheme 98 B). In the presence of aromatic amines bearing an *ortho* substituent and less nucleophilic amines, such as CbzNH_2_, the reaction furnished the product in low yield. Alkynes bearing aromatic rings or aliphatic side chains were successfully used. Methyl 2‐formyl benzoates containing electron‐withdrawing groups at the *para* position with respect to the aldehyde functional group did not afford the desired isoindolinone products. In general, lower reaction yields were observed using methyl 2‐formyl benzoates bearing electron withdrawing substituents by decreasing the nucleophilicity of the aromatic secondary amines. The absolute configuration of the products was assigned as *S* and the authors showed that steric hindrance adjacent to the aldehyde resulted in significant drop in enantiomeric purity. Later, the same group used the alkynylation/lactamization protocol as a key step in the synthesis of medicinally important isoindolinones.^[187]^ Maiti^[188]^ applied a copper(II) based heterogeneous nanocatalyst for similar transformation in good yields but only a few examples towards the synthesis of isoindolinones were explored (Scheme 98 C).

## Cycloaddition, Cyclotrimerization and Annulation Reactions

### Gold catalyzed reactions

#### Au‐catalyzed cycloaromatization

**Scheme 99:**
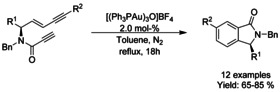
Synthesis of isoindolinones via gold‐catalyzed cascade cycloaromatization.

A gold‐catalyzed cascade cycloaromatization to a broad range of enantioenriched isoindolinones from unconjugated (*E*)‐enediynes was described by Zamani et al. (Scheme 99).^[189]^ It was shown that substituents at the enyne (R^2^) had most effect on the yield of the reaction but significant trends cannot be drawn based on the limited substrate scope. Based on experimental and computational evidence, the reaction was shown to proceed via a dual‐gold σ,π‐activation mode, involving a key gold‐vinylidene‐ and allenyl‐gold‐containing intermediate.

### Ruthenium and cobalt catalyzed reactions

#### Ru or Co‐catalyzed formal [2+2+2] cycloaddition

**Scheme 100:**
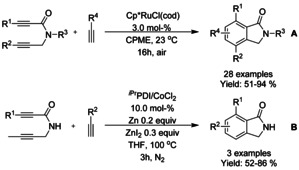
Synthesis of isoindolinones via formal [2+2+2]‐cycloaddition.

Sheppard^[190]^ reported a ruthenium catalyzed formal [2+2+2] cycloaddition between amide tethered diynes and alkynes, generating a broad scope aryl substituted isoindolinones in highly regioselective manner (Scheme 100 A). While in most cases the isolated yields were from moderate to excellent, depending on the diyne/alkyne combination applied, a significant amount of homocoupling of the diynes was observed. This could however be mitigated by dropwise addition of the diyne to the reaction mixture. Méndez‐Gálvez et al.^[191]^ also utilized a similar approach with cobalt catalyst to afford *N*‐unsubstituted isoindolinones, albeit only a few isoindolinone examples were reported (Scheme 100 B).

### Palladium and nickel catalyzed reactions

#### Pd‐catalyzed [4+2] cross‐benzannulation

**Scheme 101:**
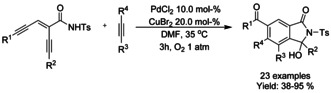
Palladium and copper co‐catalyzed (4+2) cross‐benzannulation between enediynes and alkynes.

Ma^[192]^ reported an oxidative palladium and copper co‐catalyzed [4+2] cross‐benzannulation leading to diversely substituted isoindolinones from enediynes and alkynes (Scheme 101). For the reaction to take place, aromatic substituents at R^1^ were required, whereas aromatic substituents at R^2^ would improve outcome of the reactions. Both diaryl and dialkyl alkynes were well tolerated with few exceptions, but the regioselectivity with unsymmetrical alkynes was highly dependent on the substrate. Based on experimental observations the reaction was suggested to proceed by initial 5‐*endo* cyclization followed by a *syn* carbopalladation. Subsequent nucleophilic attack of H_2_O and Cu^II^ mediated one‐electron oxidation followed by oxidation with O_2_ would then provide the target compounds.

#### Ni‐catalyzed annulation

**Scheme 102:**
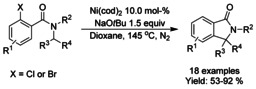
Nickel catalyzed synthesis of isoindolinones via annulation of benzamides.

Kalyani^[193]^ described a bis(cyclooctadiene)nickel(0) catalyzed arylation of *ortho*‐halo benzamides with sodium *tert*‐butoxide forming a variety of isoindolinones in moderate to high yields (Scheme 102). In general secondary and preferable tertiary carbon center was required adjacent to the amide as methyl substituted amides resulted in very low yields. It was shown that the reaction most likely proceed via radical intermediates which is align with observations on the increased efficiency of the arylation with more substituted alkyl C−H bonds adjacent to amide.

### Iridium catalyzed reactions

#### Ir‐catalyzed annulation

**Scheme 103:**
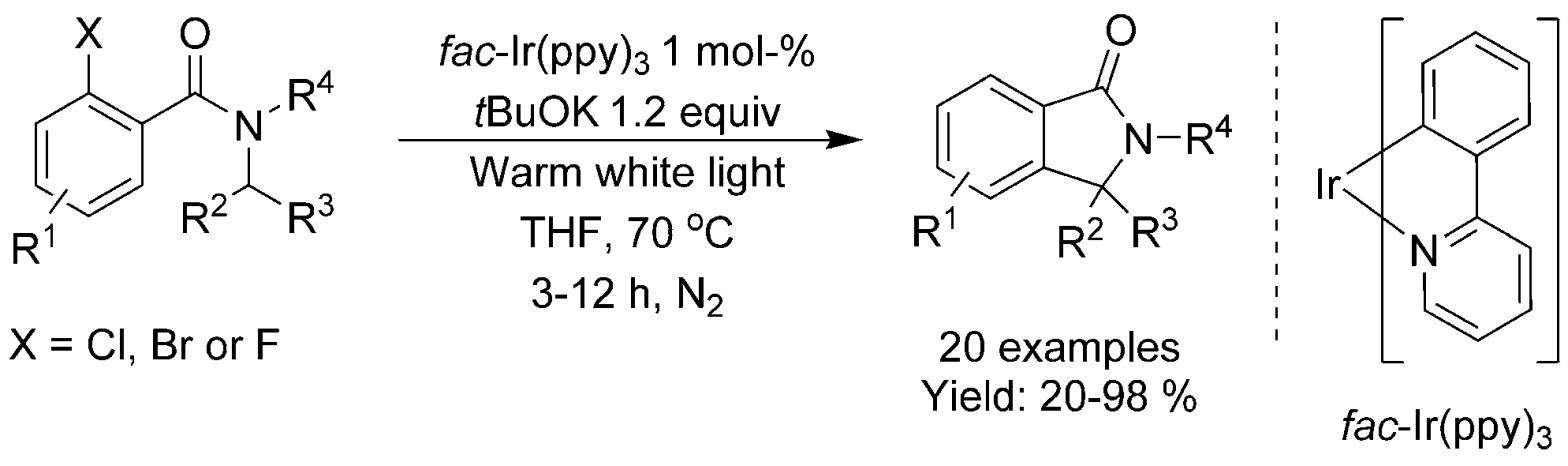
Visible‐light induced Iridium catalyzed synthesis of isoindolinones from benzamides.

A similar to method presented Kalyani,^[193]^ a light mediated quaternary annulation protocol was reported by Dai et al.^[194]^ Here, strongly reducing iridium species based on tris[2‐phenylpyridinato‐C^2^,*N*]iridium(III) (*fac*‐Ir(ppy)_3_) was used as catalyst and potassium *tert*‐butoxide as the terminal reductant (Scheme 103). Even at 50 ppm catalyst loadings the quaternary annulation was able to compete with the uncatalyzed nucleophilic aromatic substitution, leading into full retention of the enantiomeric information. Reactions using *ortho*‐fluorinated, chlorinated or brominated benzamides provided good to excellent yields with the exception of substrates containing multiple halogens in the aryl ring.

## Summary and Outlook

During the past ten years a significant amount of transition‐metal catalyzed methods applied to the synthesis of isoindolinones have been reported. By the methods presented above a vast number of isoindolinone derivatives can be accessed, without even taking into account the multitude of methods not employing transition metal catalysts. One of the most prominent fields of research relating to isoindolinone synthesis has been C−H functionalization reactions. While these methods often allow convenient routes from mostly commercially available compounds to isoindolinones, they are commonly prepared with super stoichiometric amounts of the oxidant or the additives. In both C−H functionalization and carbonylation based methodologies the use of temporary and sometimes‐permanent directing groups are a common feature. To date only a few examples of reactions with truly transient/traceless directing groups or methods with minimal need for additional reagents are presented.

In the future, increase in the amount of reports concerning enantioselective and multicomponent, directing group free methodologies can be expected. Interestingly, while the iron catalyzed methods have gained significant attention, to our knowledge, applications towards isoindolinone synthesis via C−C bond formation have not been described during the last decade. Similarly, the heterogeneous transition metal catalyzed methods for synthetic organic chemistry applications are limited when preparative approaches towards isoindolinones are considered.

## Conflict of interest

The authors declare no conflict of interest.

## Biographical Information

*Dr. Savela received his MSc from the University of Turku, followed by PhD from Åbo Akademi University, Laboratory of Organic Chemistry. During his PhD studies, he focused on applied Lewis acid catalysis for synthetic organic chemistry. Currently he is working as a postdoctoral researcher at Åbo Akademi University, Laboratory Molecular Science and Technology*.

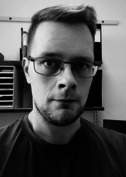

## Biographical Information

*Carolina Méndez‐Gálvez received her Ph.D. in 2013 from University of Santiago, in the group of Prof. Bruce Cassels. In 2011 she was a visiting Ph.D. student in Prof. Julio Seijas group at the University of Santiago de Compostela, Spain. From 2015 to 2017 she was a Postdoctoral Researcher at the University of Bristol, UK, under the supervision of Prof. Robin Bedford. In 2018, she moved to Finland with a fellowship from Otto A. Malm foundation to join the group of Prof. Reko Leino at Åbo Akademi University. Her research interests include synthetic organic chemistry and homogeneous catalysis*.